# Reconstruction of Cell Surface Densities of Ion Pumps, Exchangers, and Channels from mRNA Expression, Conductance Kinetics, Whole-Cell Calcium, and Current-Clamp Voltage Recordings, with an Application to Human Uterine Smooth Muscle Cells

**DOI:** 10.1371/journal.pcbi.1004828

**Published:** 2016-04-22

**Authors:** Jolene Atia, Conor McCloskey, Anatoly S. Shmygol, David A. Rand, Hugo A. van den Berg, Andrew M. Blanks

**Affiliations:** 1 Division of Reproductive Health, Warwick Medical School, University of Warwick, Coventry, United Kingdom; 2 University of Warwick, Coventry, United Kingdom; University of Michigan, UNITED STATES

## Abstract

Uterine smooth muscle cells remain quiescent throughout most of gestation, only generating spontaneous action potentials immediately prior to, and during, labor. This study presents a method that combines transcriptomics with biophysical recordings to characterise the conductance repertoire of these cells, the ‘conductance repertoire’ being the total complement of ion channels and transporters expressed by an electrically active cell. Transcriptomic analysis provides a set of potential electrogenic entities, of which the conductance repertoire is a subset. Each entity within the conductance repertoire was modeled independently and its gating parameter values were fixed using the available biophysical data. The only remaining free parameters were the surface densities for each entity. We characterise the space of combinations of surface densities (density vectors) consistent with experimentally observed membrane potential and calcium waveforms. This yields insights on the functional redundancy of the system as well as its behavioral versatility. Our approach couples high-throughput transcriptomic data with physiological behaviors in health and disease, and provides a formal method to link genotype to phenotype in excitable systems. We accurately predict current densities and chart functional redundancy. For example, we find that to evoke the observed voltage waveform, the BK channel is functionally redundant whereas hERG is essential. Furthermore, our analysis suggests that activation of calcium-activated chloride conductances by intracellular calcium release is the key factor underlying spontaneous depolarisations.

## Author Summary

A well-known problem in electrophysiologal modeling is that the parameters of the gating kinetics of the ion channels cannot be uniquely determined from observed behavior at the cellular level. One solution is to employ simplified “macroscopic” currents that mimic the behavior of aggregates of distinct entities at the protein level. The gating parameters of each channel or pump can be determined by studying it in isolation, leaving the general problem of finding the densities at which the channels occur in the plasma membrane. We propose an approach, which we apply to uterine smooth muscle cells, whereby we constrain the list of possible entities by means of transcriptomics and chart the indeterminacy of the problem in terms of the kernel of the corresponding linear transformation. A graphical representation of this kernel visualises the functional redundancy of the system. We show that the role of certain conductances can be fulfilled, or compensated for, by suitable combinations of other conductances; this is not always the case, and such “non-substitutable” conductances can be regarded as functionally non-redundant. Electrogenic entities belonging to the latter category are suitable putative clinical targets.

## Introduction

The human genome contains a large number of distinct ion channels, each of which may be assembled into multimeric complexes that modulate the electrical activity of the cell in a subtly different way [1]. Determination of the total ion channel repertoire of a given cell (its ‘conductance repertoire’) is cumbersome via conventional biophysical techniques. The latter rely on the specificity and availability of suitable pharmalogical agents or protocols, which may or may not be able to differentiate between combinations of conductances that can give rise to similar behaviors at the electrophysiological level. By contrast, transcriptomic analysis accurately surveys the complete complement of mRNA coding for all potential conductances. However, such data sets are semi-quantitative at best, absent a straightforward relationship between mRNA levels and surface expression/functionality of the electrogenic proteins.

The classic approach in electrophysiology has been to fit a suitable mathematical model to the data, where the parameters to be estimated represent not only the densities of the electrogenic entities but also their biophysical properties [2–4]. This approach is hampered by the large number of distinct electrogenic species. One option is to combine the contributions from several electrogenic species (which are often oligomeric complexes) into single entities, resulting in a ‘macroscopic current’ model [4–10]. Such models may not be sufficiently detailed for pharmacological purposes and accurate assessment of currents within native cells can be technically challenging.

The gold standard, therefore, is to determine the conductance repertoire at the level of individual molecular species. The present paper integrates transcriptomics with the information encoded by the action potential (AP) waveform. Electrophysiological data do not necessarily fix a unique solution; there are infinitely many alternative conductance repertoires, that are all equally compatible with the available electrophysiological data. We should consider all conductance repertoires that are consistent with the data, and focus on the properties that they share *as a class*. This motivates a formal characterisation of the functional redundancy of the system.

Our approach is based on two key ideas. First, we use two kinds of data to constrain the space of possibilities, namely mRNA sequencing to determine which species the cell is capable of expressing, combined with parameter estimation to determine the conductance repertoire from electrophysiological data. Second, we obtain the parameters associated with the gating kinetics from the analysis of published data, which means that the *only* remaining free parameters are the surface densities of the species on the list compiled from transcriptomics.

We focus on a cell type of considerable clinical interest, namely the myometrial smooth muscle cell (MSMC), which is the principal unit of electrical activity in the uterus, normally quiescent except in labor, when spontaneous action potentials are generated by the MSMC [11]. A detailed, comprehensive characterisation of these cells would aid the development of therapeutics to manage both preterm birth and perinatal complications associated with uterine contractility, such as postpartum haemorrhage [12]. From a pharmacological point of view, the most promising targets are electrogenic entities whose contribution to the overall electrophysiological behavior is essential in the sense that the role of such an entity cannot be fulfilled by some combination of other ion channels. We show that our analysis is capable of identifying such entities and verify these predictions by means of additional experiments.

## Results

We formulated a mathematical model based on individual currents carried by the electrogenic transmembrane proteins in the human MSMC. There are four steps: (i) characterise the potential repertoire of electrogenic proteins by means of expression studies; (ii) model each ion channel or transporter *independently* and determine its gating parameter values on the basis of data taken from the literature; (iii) characterise the space of possible solutions of conductance repertoires consistent with the experimentally observed calcium and membrane potential waveforms; (iv) select a putative conductance repertoire from this set and use this in subsequent ‘free-running’ simulations in order to explore various hypotheses.

**Fig 1:**
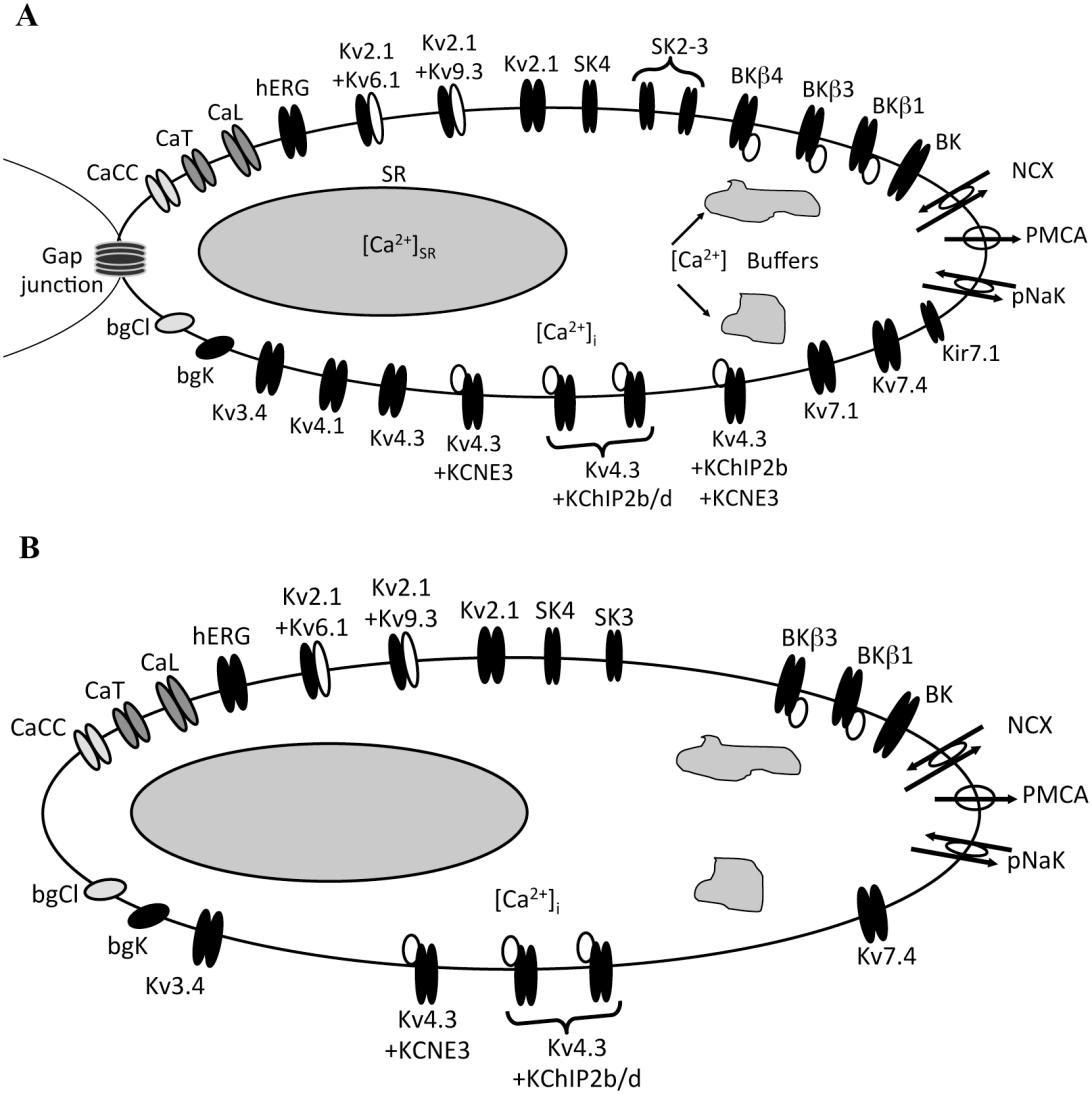
Model diagram. (A) Diagrammatic representation of all potential entities considered in the model repertoire according to mRNA expression data [13]. (B) Diagrammatic representation of the potential entities based on the most parsimonious solution (ℓ_1_-norm; see text for further details). SR: sarcoplasmic reticulum; CaCC: calcium-activated chloride channel; CaT: T-type calcium channel; CaL: L-type calcium channel; hERG: human *ether-à-go-go*-related gene potassium channel; Kvx.x: potassium channel; SK-x: “small unitary current” potassium channels; BK-x: “big unitary current” potassium channels; NCX: sodium-calcium exchanger; PMCA: plasma-membrane calcium exchanger; pNaK: sodium-potassium exchanger; bgK: background potassium current; bgCl: background chloride current.

We determined the repertoire of the electrogenic proteins that are potentially expressed in MSMC, as indicated by mRNA expression data [13]. These proteins are often subunits that contribute to functional channels in various combinations. The expression list therefore generates a potential repertoire of conducting entities that may be present in the plasma membrane of the MSMC; a list of all potential entities considered in the model is given in Table 1. This list is depicted diagrammatically in Fig 1A.

**Table 1:** Potential conductance species included in the model.

Name given	Gene combination	State variables	References
Kv2.1	KCNB1	$C_{0}^{\text{[Kv2.1]}}$–$C_{4}^{\text{[Kv2.1]}}$	[14]
		*O*^[Kv2.1]^	
		$I_{0}^{\text{[Kv2.1]}}$–$I_{5}^{\text{[Kv2.1]}}$	
Kv9.3	KCNB1+KCNS3	*g*_1_, *g*_2_fast__, *g*_2_slow__	[15]
Kv6.1	KCNB1+KCNG1	*l*_1_, *l*_2_	[15, 16]
BK	KCNMA1	*P*_*o*BK_*α*__	[17]
BK*β*1	KCNMA1+KCNMB1	*P*_*o*BK_*α*+*β*1__	[17]
BK*β*3	KCNMA1+KCNMB3	$C_{0}^{\left[\alpha +\beta 3\right]}$–$C_{4}^{\left[\alpha +\beta 3\right]}$	[18]
		$O_{0}^{\left[\alpha +\beta 3\right]}$–$O_{4}^{\left[\alpha +\beta 3\right]}$	
		$I_{0}^{\left[\alpha +\beta 3\right]}$–$I_{4}^{\left[\alpha +\beta 3\right]}$	
BK*β*4	KCNMA1+KCNMB3	$P_{\text{BK}{}_{\alpha +\beta 4}}$	[19, 20]
SK_2_	KCNN1	*P*_SK2_	[21, 22]
SK_3_	KCNN3	*P*_SK3_	[23]
SK_4_	KCNN4	*P*_SK4_	[24]
hERG	KCNH2	$C_{0}^{\text{[hERG]}}$–$C_{2}^{\text{[hERG]}}$	[25]
		$O_{1}^{\text{[hERG]}}$–$I_{0}^{\text{[hERG]}}$	
CaL (L-type)	CACNA1C	*d*, *f*	[3]
CaT (T-type)	CACNA1G	*a*, *c*	[26]
CaCC	ANNO1	*cc*	[27]
Kv3.4	KCNC4	*a*_1_, *a*_2_	[28]
Kv4.1	KCND1	*b*_1_, *b*_2_fast__, *b*_2_inter__, *b*_2_slow__	[29, 30]
Kv4.3	KCND3	$C_{0}^{\text{[Kv4.3]}}$–$C_{4}^{\text{[Kv4.3]}}$	[31]
		*O*^[Kv4.3]^	
		$I_{0}^{\text{[Kv4.3]}}$–$I_{6}^{\text{[Kv4.3]}}$	
Kv4.3+KCNE3	KCND3+KCNE3		
Kv4.3+KChIP2b	KCND3+KChIP2b	*k*_1_, *k*_2_fast__, *k*_2_slow__	[32]
Kv4.3+KChIP2d	KCND3+KChIP2d	*k*_1_, *k*_2_fast__, *k*_2_slow__	[32]
Kv4.3+KChIP2b+KCNE3			
Kv7.1	KCNQ1	$C_{1}^{\text{[Kv7.1]}}$, $C_{2}^{\text{[Kv7.1]}}$	[33]
		$O_{1}^{\text{[Kv7.1]}}$, $O_{2}^{\text{[Kv7.1]}}$	
		*I*^[Kv7.1]^	
Kv7.4	KCNQ4	*d*_1_, *d*_2_	[34]
Kir7.1	KCNJ13	*P*_Kir7.1_	[35]
bgK, bgCl			
Gap1, Gap2		*P*_GJ_*I*__, *P*_GJ_*II*__	[36]
NCX, PMCA, NaK			[3, 37]
P2X4	P2X4R	*P*_act_, *P*_des_	[38]

Various potential conductance species considered in the model based on mRNA expression data.

For every channel complex consistent with the subunits in the mRNA expression list and attested in the literature, a sub-model was compiled by taking a mathematical description of gating kinetics from the literature whenever available; otherwise novel equations were formulated using available data, and biophysical and kinetic parameter values were determined via least-squares fitting to experimental data on heterologous expression systems (see Materials and Methods and Supporting Information for more details).

The current summation model accomodates the contributions made by each of the conductances:

$$C\frac{d}{dt}V\left(t\right)=I\left(t\right)+\sum_{i=1}^{n}\varkappa_{i}\psi_{i}\left(t\right)$$ (1)

where *C* is the membrane capacitance, *V*(*t*) is the transmembrane voltage difference at time *t*, *I*(*t*) is the injected current, *ψ*_*i*_(*t*) is the current carried per ion channel or transporter complex, and *ϰ_i_* is the corresponding surface density of this entity. The densities can be estimated in terms of *κ_i_* = *ϰ_i_*/*C*, which expresses the number of pores of species *i* per unit of capacitance (clearly *κ*_*i*_ ≥ 0 for all *i*).

**Fig 2:**
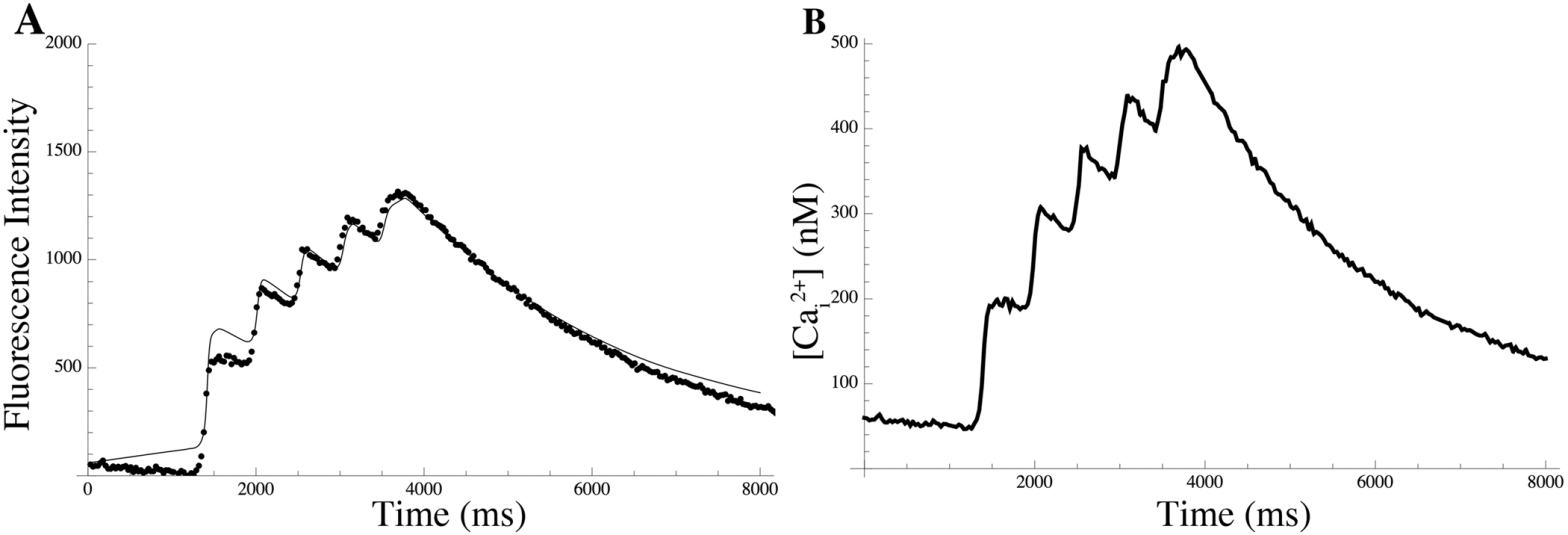
Calcium dynamics. (A) Calcium fluorescence signal (dots) together with the least-squares fit of the calcium excitation model (solid line); parameter estimates are given in Table 4. (B) Reconstructed calcium concentration time course obtained using the model with these estimates.

The parameters of the gating kinetics of each species having been determined independently, the only remaining unknowns are the cell-surface densities (Fig 1A). Native cell behavior combining two simultaneously acquired data sets (voltage *V* and intracellular calcium [Ca^2+^]*_i_* as a function of time; see Materials and Methods for detailed experimental procedures) was used to constrain the parameters. Briefly, the observed voltage and Ca^2+^ time series (Fig 2), detailed in S1 Data, were used to drive the gating kinetics of each entity (‘data-driven’ simulations, as opposed to free-running simulations that were done subsequently). The gating time series thus obtained were used to calculate the individual current densities. The observed membrane potential time series is linear in the channel densities, which implies that the kernel of a linear transformation characterises the space of all possible combinations of ion channel densities that is consistent with the data. This kernel represents the functional redundancy of the system, as it is essentially the space of all conductance repertoires that are compatible with the data. In particular, unless the transformation is of full rank, no unique conductance repertoire can be inferred from the data and the parameter vector *κ* is formally unidentifiable. This is the typical situation encountered in practice.

To overcome this problem, an additional criterion is required to select one particular vector in this space. Different criteria lead to different choices. We chose to calculate the vector with smallest ℓ_1_-norm, subject to non-negativity. This is parsimonious in the sense that it represents the most economical way for the cell to exhibit the observed physiological behavior, although we do not necessarily believe that this is the dominant operative evolutionary criterion.

An estimate of the channel densities up to the indeterminacy of the kernel is obtained. The scaled surface density vector *κ* is given as the sum of a particular solution *κ*_*p*_ plus an arbitrary linear combination of the kernel’s basis vectors. For *κ*_*p*_ we can take the pseudo-inverse *κ*^+^, which has the property that the discrepancy between the observed *V*(*t*) and the model prediction is minimised in the least-squares sense (*κ*^+^ has the smallest Euclidian norm out of all vectors that achieve this best fit). However, the affine space formed of *κ*^+^ plus the basis vectors is not guaranteed to intersect with the non-negative cone. Only this intersection is relevant because channel densities cannot be negative. Constrained non-linear least-squares fitting was used to find a vector in the non-negative cone that was minimal in the ℓ_1_-norm. This vector is listed in Table 2 and Fig 1B. Figure D2 in McCloskey et al [39] shows the observed voltage trace together with the free-running simulation using *κ*^+^ and the free-running simulation using the non-negativity-constrained, minimal-ℓ_1_ vector.

**Table 2:** Parameter estimation.

**Potential conductance species**	**number of channels/pF**
Kv2.1	15.7
Kv9.3	6
Kv6.1	0.74
BK	0.064
BK*β*1	0.3
BK*β*3	0.037
BK*β*4	0
SK_2_	0
SK_3_	0.78
SK_4_	0.015
hERG	2.4
CaL (L-type)	10.5
CaT (T-type)	19.8
CaCC	0.45
Kv4.1	0
Kv4.3	0
Kv4.3+KCNE3	1.3
Kv4.3+KChIP2b	2.7
Kv4.3+KChIP2d	0.3
Kv4.3+KChIP2b+KCNE3	0
Kv3.4	5.2
Kv7.1	0
Kv7.4	0.84
Kir7.1	0
bgK, bgCl	0.5, 0.46
Gap1	0
Gap2	0
**Potential conductance species**	**channel density factor**
NCX	0.03
PMCA	3.9
NaK	1.1

### Validation of the estimated conductance repertoire

Physiological predictions were made, using the simulation model in its free-running mode, on the basis of the conductance repertoire as per the minimal ℓ_1_-norm criterion. The predictions were validated against experimental data obtained from voltage-clamp experiments for currents isolated by antagonist application. Sets of currents were measured for control and antagonist-inhibited current, and specific current was obtained by subtracting the current recorded in the presence of antagonist from the total current; measurements were reported as mean current at 450 ms ± SEM. The simulations were entirely independent of these additional data.

To inhibit and thereby isolate the Kv2.1 currents, we applied stromatoxin (ScTx), an inhibitor of Kv2.1 homomeric channels, Kv2.1/Kv6.1, Kv2.1/Kv9.3, as well as the A-type channel Kv4.2. The late outward current was measured to separate Kv2.1 from A-type currents. The measured ScTx-sensitive late outward current (at *t* = 450 ms), resulting from voltage-clamp experiment at 40 mV after application of 100 nM ScTx, was 3.71±0.41 pA/pF (*n* = 19). The total peak Kv2.1 current elicited by the simulation for the same voltage step (40 mV) was 3.77 pA/pF (the channels Kv2.1, Kv6.1, and Kv9.3 contribute to this current).

Dofetilide is a selective inhibitor of hERG channels. The isolated dofetilide-sensitive current at 450 ms observed in voltage-clamp experiments at 40 mV after the application of 1 *μ*M dofetilide was 1.24±0.61 pA/pF (*n* = 7), as compared to 1.544 pA/pF for the simulated current elicited by hERG at the same voltage.

**Fig 3:**
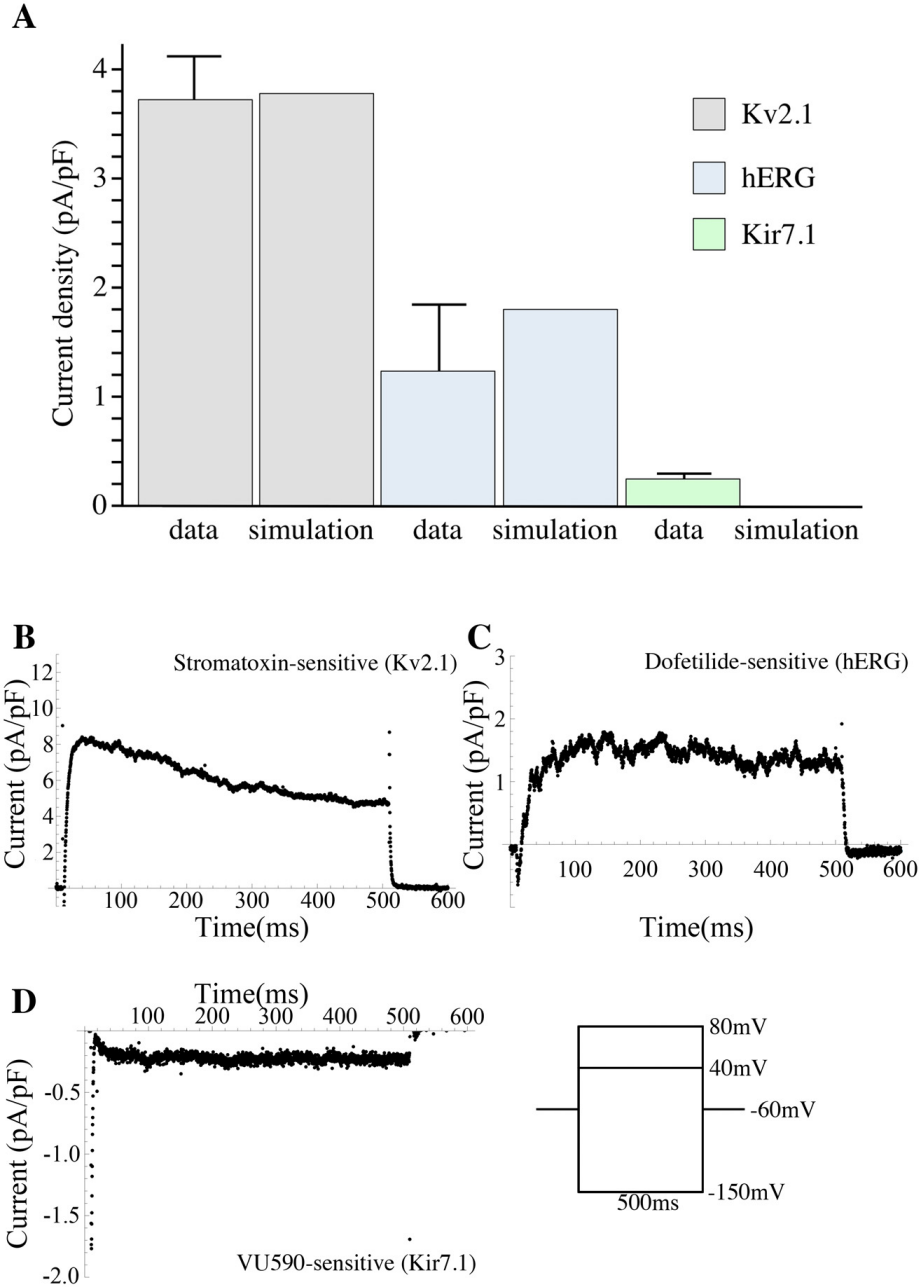
Validation of the model. (A) Bar chart comparing experimental and simulated Kv2.1, hERG, and Kir7.1 current densities (means±SEM). (B, C, D) ScTx-, dofetilide-, and VU590-sensitive currents in day-15 (D15) mice samples, the asterisk indicating at which point in time the current was sampled for the data reported in A; experimental traces under voltage-clamp conditions with 40 mV voltage step for ScTx- and dofetilide-sensitive currents and −150 mV for VU590-sensitive current from holding potential of −60 mV (ScTx: *n* = 19; dofetilide: *n* = 7; VU590: *n* = 9). A detailed comparison of measured and simulated currents is reported in S1 Fig. Voltage time series data are provided in S2 Data.

Fig 3 shows the experimental current densities under the voltage-clamp conditions. The simulated values of peak currents at selected voltage steps agree, to within the margins of experimental error, with the currents measured for Kv2.1 channels and hERG channel.

### Representation of the functional redundancy of the MSMC

**Fig 4:**
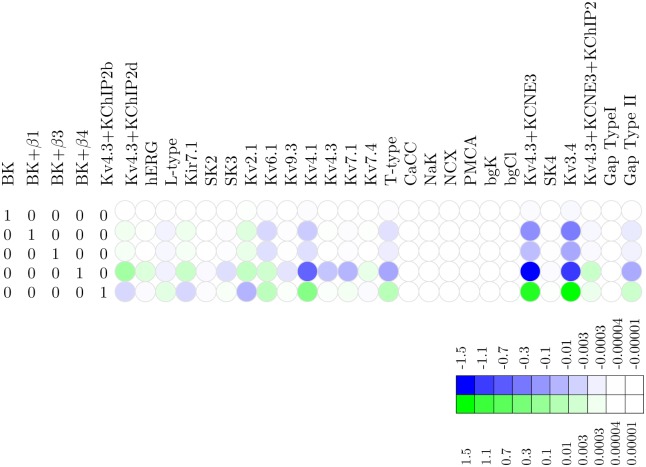
Redundancy map for the BK potassium channel. Heat map of the null space matrix in echelon form with BK as a leading variable, showing which linear combinations of other channels can compensate for shifts in BK density. Whereas shifts in the BK_*α*_ density require virtually no compensation to maintain the voltage waveform, shifts in e.g. BK_*α*_ + *β*4 require large compensatory shifts in several other channels, both upward and downward. The color key relates hue to the *log*_10_ of the fold change.

**Fig 5:**
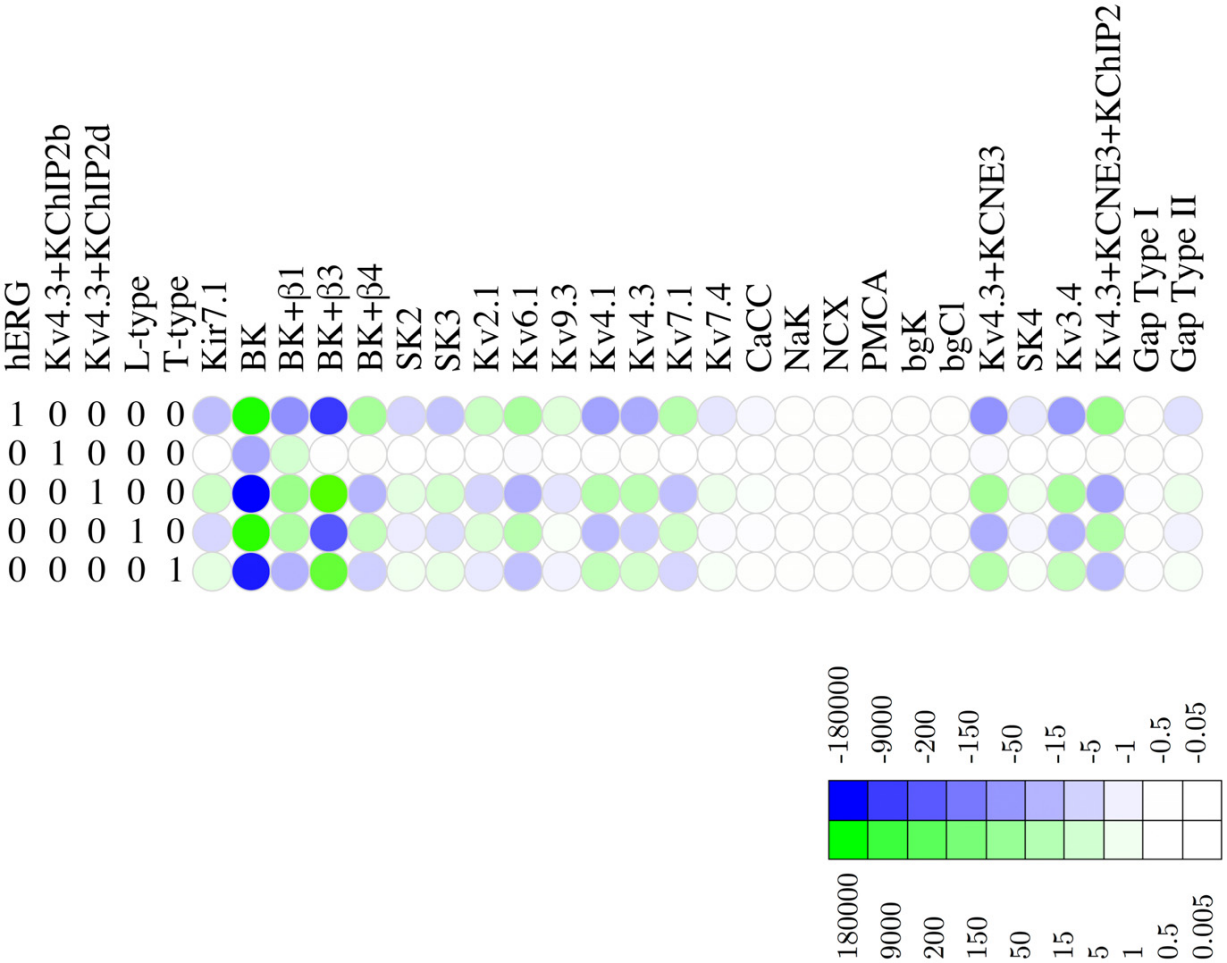
Redundancy map for the hERG potassium channel. Heat map of the null space matrix in echelon form with hERG as a leading variable, showing which linear combinations of other channels can compensate for shifts in BK density. Shifts in e.g. hERG require large compensatory shifts in several other channels. The color key relates hue to the *log*_10_ of the fold change.

The functional redundancy is encoded by the kernel of a linear transformation. An informative graphical representation of this functional redundancy can be obtained by transforming the basis of the kernel to a suitable echelon form, which is then visualised as a heat map. These *redundancy maps*, shown in Figs 4 and 5, are to be read one row at a time. A unit change in the density of the channel appearing in the leading (left-most) position of each row can be functionally compensated, by upward or downward shifts represented by the trailing elements (i.e., those to the right). The redundancy map thus represents a compensation signature, charting the cell’s ability of exhibiting the same electrophysiological behavior despite altered channel densities. This compensation is computed on the basis of the voltage waveform that was used to derive the basis vectors, but it is not unreasonable to expect that the compensatory adjustments prescribed by the redundancy map are applicable for a wider range of behaviors. An additional caveat is that these adjustments are exact only for infinitesimal changes in the leading channel density; for finite perturbations in the leading variable, the corresponding shifts dictated by the trailing elements are only a first-order approximation. This means that there is a finite operating range within which the approximation can be expected to be reasonably accurate. Moreover, the operating range is further constrained by considerations of physiologic feasibility: channel densities cannot be negative and, similarly, there will be a upper bound to the current density that can be physically sustained by the cell membrane (there will also be a physiological upper bound to the quantities of channel proteins that can be synthesised and inserted into the membrane). To estimate such upper bounds we used the procedure detailed in Materials and Methods, “Physiological upper bounds on channel density values.”

#### BK

**Fig 6:**
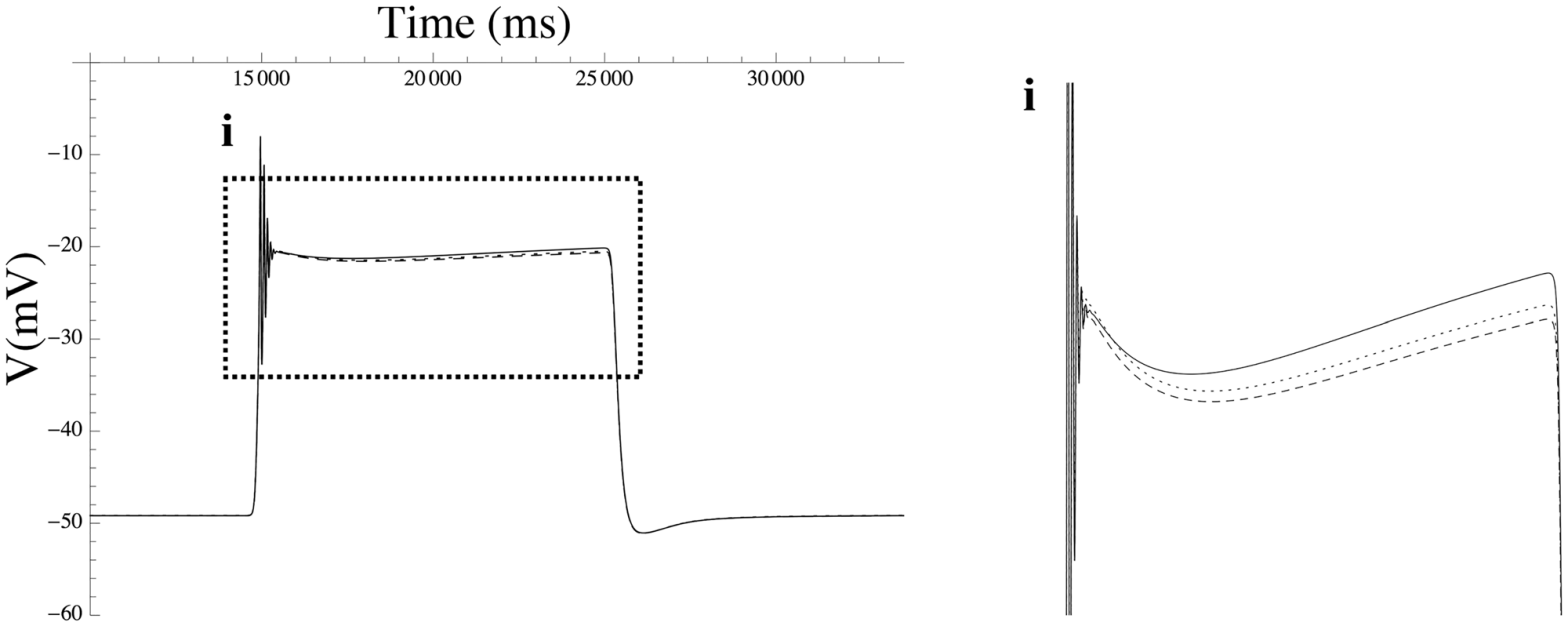
The effect of a shift in the BK channel density on the action potential. The effect of varying the BK channel density on the action potential triggered by a 10-sec extracellular ATP step function, compared to compensatory corrections of the densities of the various other channels according to the redundancy map shown in Fig 4. The solid line shows the reference waveform, which is produced by running the simulation model in free-running mode with parameter values constrained by the ℓ_1_-norm. The dashed line shows the distortion caused by a small change in BK density, while the dotted line shows the waveform when compensatory shifts in other channels are applied in addition to the BK perturbation, partially restoring the waveform back to the reference waveform. (i) enlarged detail.

To investigate the functional redundancy of the BK channel and its isoforms, we took these channels as leading variables in the echelon form, resulting in the redundancy map shown in Fig 4. This map indicates that minute adjustments suffice, i.e., the effect of BK can be compensated by extremely small variations in the densities of other channels. Fig 6 depicts the effect of increasing the BK channel density by a single-channel per pF on the membrane potential waveform. The changes in the densities of the various channels, according to the values shown in the echelon map of Fig 4, compensate for the effect of shifting the BK channel density. This points to a substantial degree of functional redundancy: apparently, the biological role of BK channels is readily fulfilled by other channels (at least for the waveform under consideration here). For instance, shifts for BK+*β*1 can be compensated by A-type and Kv2.1 (Fig 4). This redundancy is consistent with the finding by Aaronson et al that inhibition of BK channels has no effect on uterine contractions *in vitro* [40].

#### hERG

The adjustments prescribed by the redundancy map translate into extremely large compensatory shifts that are physiologically implausible; the channel is effectively pinned because even a minute change in its density cannot be compensated by a combination of adjustments in other channels. A point in case is the channel hERG, where a slight decrease of 1 channel/pF in the hERG channel density would require ∼200,000 channels/pF increase in BK, ∼10,000 channels/pF decrease in BK_*α*+*β*_3__, and an increase of ∼150 channels/pF in the Kv4.3/KCNE3/KChIP2, as shown in Fig 5. The redundancy map thus suggests that hERG is non-compensatable (relative to the observed voltage waveform). In biological terms, this suggests that hERG is functionally non-redundant, i.e., *essential*. This conclusion agrees with the experimental finding that application of the hERG antagonist dofetilide initiates prolonged depolarisations [41].

#### SK

**Fig 7:**
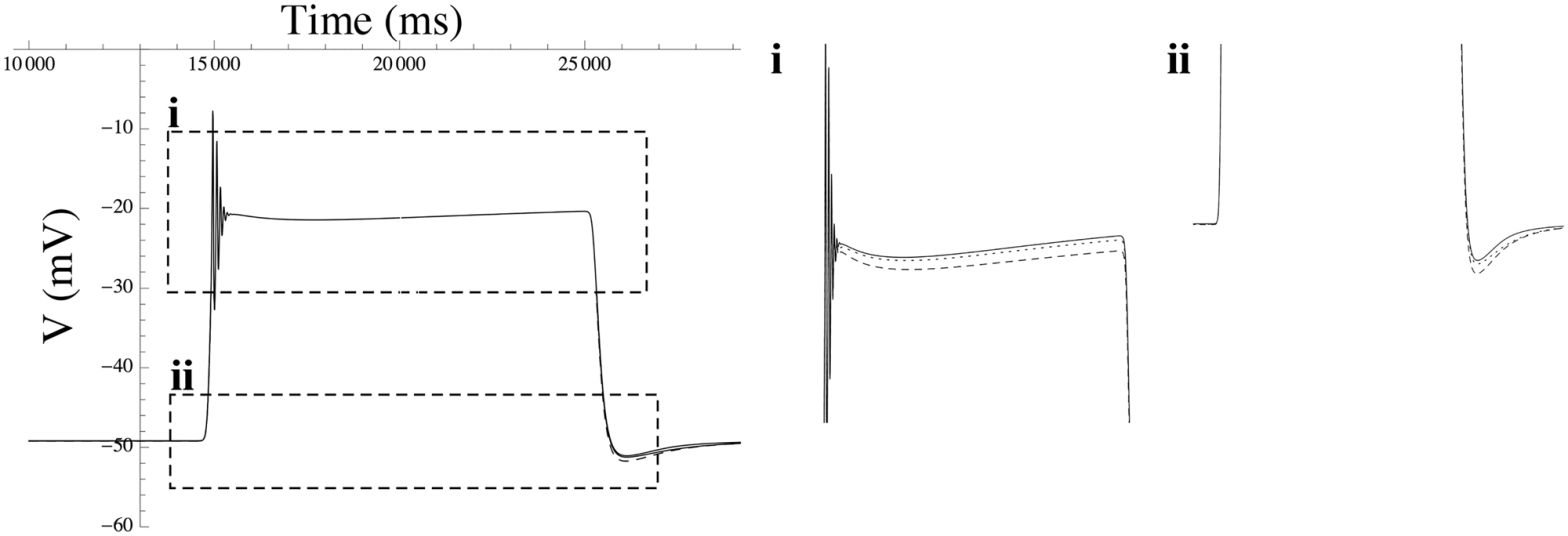
Effect of a shift in the SK_2_ channel density on the action potential. The effect of varying the SK_2_ channel density on the action potential triggered by a 10-sec extracellular ATP step function, compared to compensatory corrections of the densities of the various other channels according to the redundancy map. The solid line shows the reference waveform, which is produced by running the simulation model in free-running mode with parameter values constrained by the ℓ_1_-norm. The dashed line shows the distortion caused by a small change in SK_2_ density, while the dotted line shows the waveform when compensatory shifts in other channels are applied in addition to the SK_2_ perturbation, partially restoring the waveform back to the reference waveform. (i, ii) enlarged details.

The genes encoding the family of SK channels exhibit substantial structural homology, with little similarity to members of other potassium-channel subfamilies [23]. In MSMCs the expressed channels are SK_3_ and SK_4_ [13, 42]. The high degree of homology suggests members of the SK family should be readily interchangeable. To verify this, we selected the SK_2_ channel as a leading variable for the echelon form and found that a unit change in the latter can be compensated by an 0.49 units negative shift in the SK_3_ channel density, and 0.45 units negative shift in the SK_4_ channels density. Using the model in free-running mode, we tested the effect of increasing the SK_2_ channel density by 0.4 channels/pF on the membrane waveform. As shown in Fig 7, this perturbation can be compensated by decreasing the SK_3_ density by 0.15 channels/pF and decreasing the SK_4_ density by 0.14 channels/pF.

### Simulations to explore hypotheses on periodic triggering of depolarisation

Uterine contraction during labor is characterised by its automaticity [43]. The myogenic mechanisms underlying uterine activity are not fully understood, although it is generally accepted that a major contribution is made by the increase in the concentration of intracellular calcium of MSMCs via L-type calcium channels [43]. Here we explore three mechanisms that could result in the initiation of periodic spontaneous contractions. The first mechanism acts through the activation of the purinergic membrane receptor (P2RX4), which is an adenosine triphosphate (ATP)-gated ion channel. The second mechanism depends on the periodic depletion of phosphatidylinositol-4,5 bisphosphate (PIP_2_), which modulates the activation of a number of plasma membrane potassium ion channels. Closure of these channels would promote depolarisation and subsequent contractions. The third mechanism is based on periodic intracellular Ca^2+^ release from sarcoplasmic reticular stores, leading to activation of the Ca^2+^-activated Cl^−^ conductance ANO1.

#### Hypothesis I: Availability of ATP

We investigated the role of ATP in the depolarisation that initiates spontaneous electrical activity and hypothesised that the initiation of the depolarisation is via activation of the P2RX4 receptor by a periodic release of ATP via hemichannels [44–46]. Several lines of evidence point to the existence of a purine-gated non-specific cationic conductance in MSMCs [47–49]: the mRNA expression levels of P2RX4 and P2RX7 increase in rat myometrial samples as pregnancy progresses [47], suggesting that these receptors are associated with uterine contractions at term. Moreover, mRNA for these proteins is increased upon stimulation with lipopolysaccharide, which is a known stimulant of preterm labor [47]. In freshly dissociated rat myometrial cells, a non-specific cationic conductance consistent with the biophysical properties of P2RX7 was measured upon stimulation with ATP [48, 49]. In human MSMCs both isoforms are expressed, but P2RX4 dominates [13].

**Fig 8:**
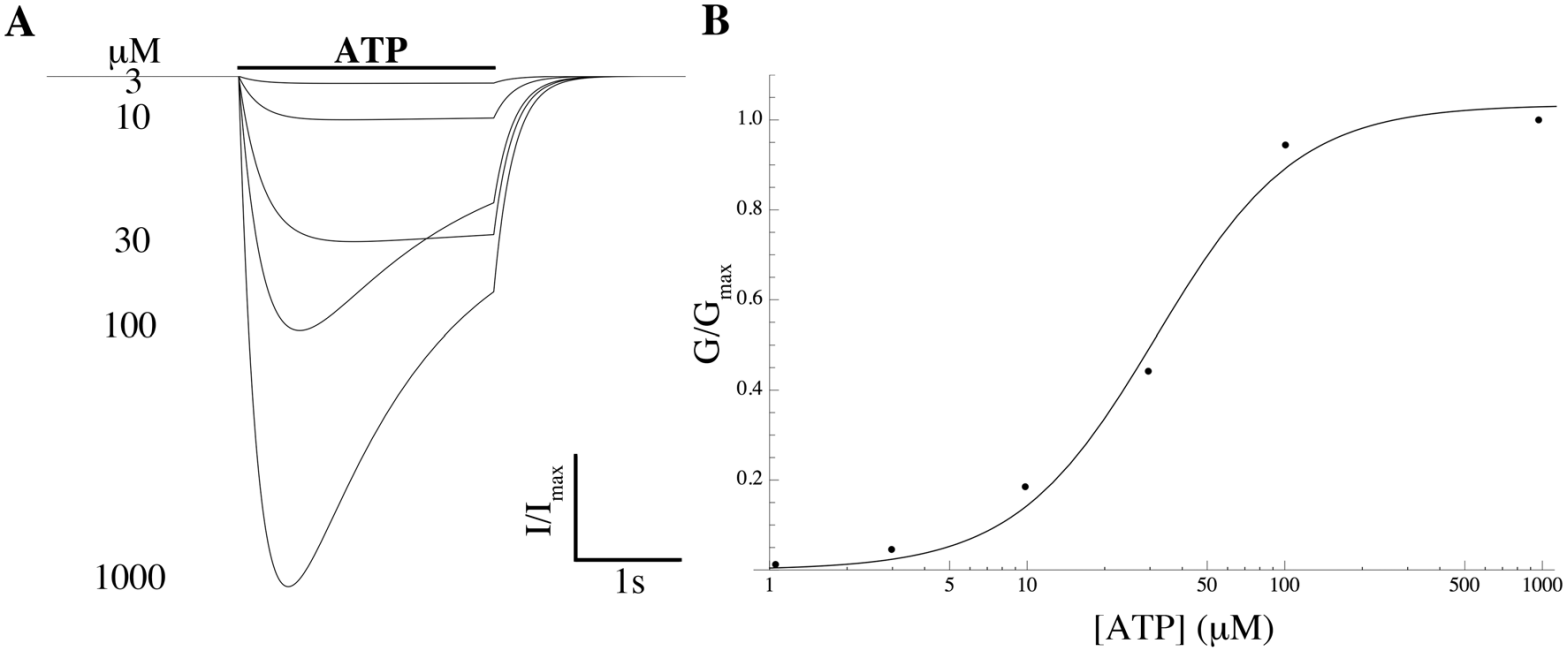
Properties of *I*_P2X4_. (A) Normalised *I*_P2X4_ theoretical current traces for various ATP concentrations at a holding potential of −60 mV. (B) Simulated ATP-concentration effect curve (solid line) and experimental data by Toulme et al [38] (solid circles). Values are normalised to the peak current values.

We modeled the current conducted by P2RX4 and added it to the model. The time course of the extracellular ATP concentration ([ATP]_o_) was assumed to be constant over a time interval of finite duration of 10 s and zero outside this interval, and this step function was used as a forcing function to drive the ATP-gated kinetics of P2RX4, in free-running simulations using the conductance repertoire found for the minimal ℓ_1_-norm criterion. The P2RX4 channel is activated by [ATP]_o_, evoking an inward current that activates and deactivates rapidly and desensitises relatively slowly (within seconds of continuous agonist application) [50]. The rates of activation and desensitisation are dependent on [ATP]_o_, whereas the activation and desensitisation time constants are inversely correlated with [ATP]_o_ [50]. The ATP-dependence of the steady-state of the P2RX4 activation variable was modeled by the Hill equation which was fitted to experimental data [38], giving an EC_50_ of 32 *μ*M and a Hill coefficient of 1.6 (Fig 8B). The time constants of activation and desensitisation *τ*_act_ and *τ*_des_, were determined by fitting a single exponential to current data [38]. The simulated current trace is shown in Fig 8A.

**Fig 9:**
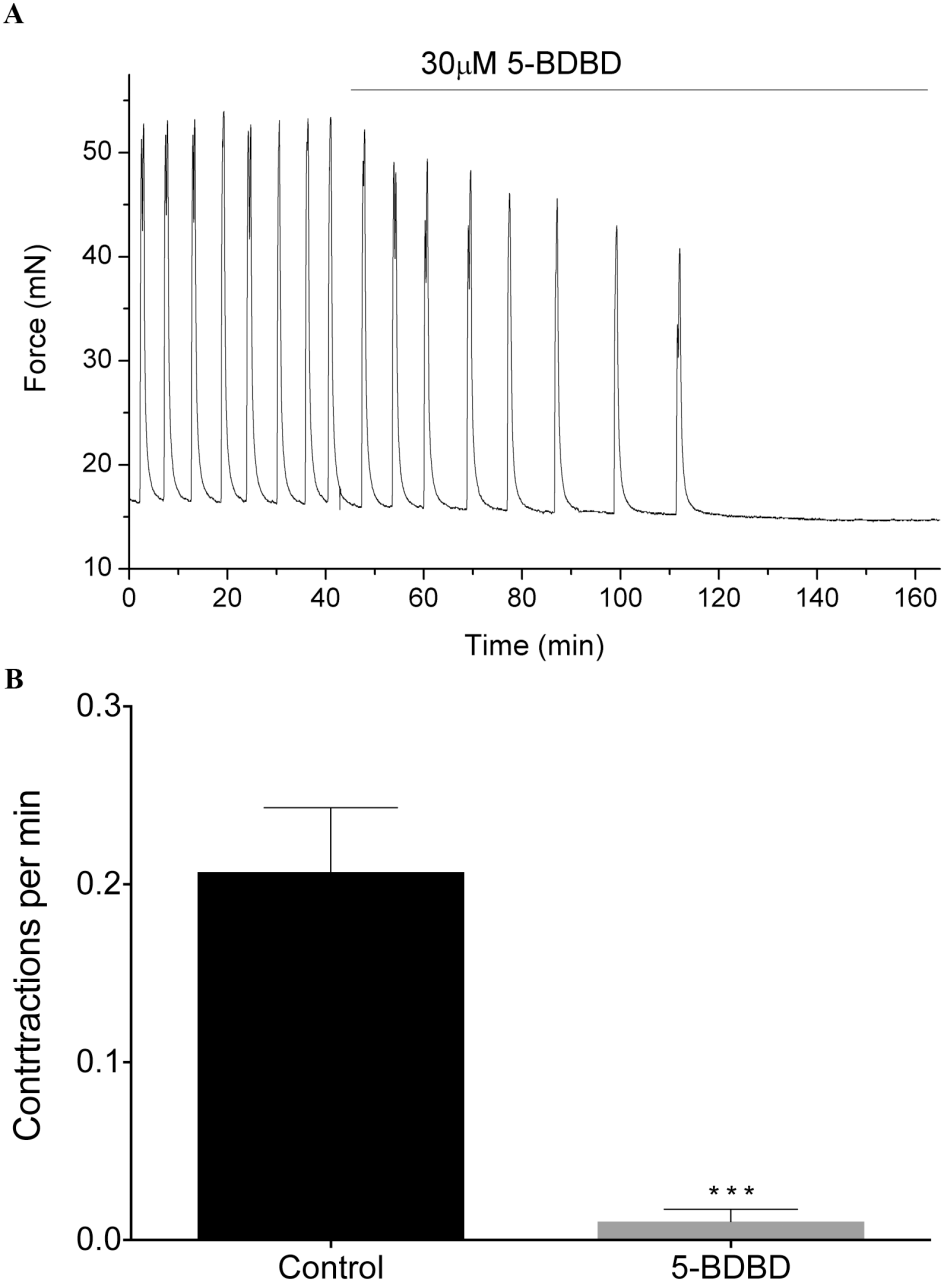
Effect of increasing the extracellular ATP concentration. Simulation of the mathematical model in free-running mode, showing the effect of increasing the extracellular ATP concentration on the myometrium AP waveform. Key to ATP concentrations during 10-sec application: light blue: nil; blue: 10 *μ*M; black: 100 *μ*M; green: 1 mM; and red: 10 mM.

The channel P2RX4 has a unitary conductance of ∼36 pS; it is impermeable to Cl^−^, highly permeable to Ca^2+^, and equally permeable to Na^+^ and K^+^ with a ratio of calcium to monovalent ion permeability of 4.2 [51]. To calculate *E*_P2RX4_ (the reversal potential of the P2RX4), we used the Goldman-Hodgkin-Katz voltage equation [1, 52, 53]. The current carried by P2RX4 is given by the following equation:

$$I_{\text{P2RX4}}=\kappa_{\text{P2RX4}}G_{\text{P2RX4}}P_{\text{act}}P_{\text{des}}\left(V-E_{\text{P2RX4}}\right)\;,$$ (2)

where *κ*_P2RX4_ is the P2RX4 channel density, *G*_P2RX4_ is the unitary conductance and *P*_act_ and *P*_des_ are the activation and desensitisation gating variables, respectively. We set *κ*_P2RX4_ = 90 channels/pF; further details can be found in the Supplementary Material. Fig 9 confirms that the 10-second temporary elevation of [ATP]_o_ leads to depolarizations.

**Fig 10:**
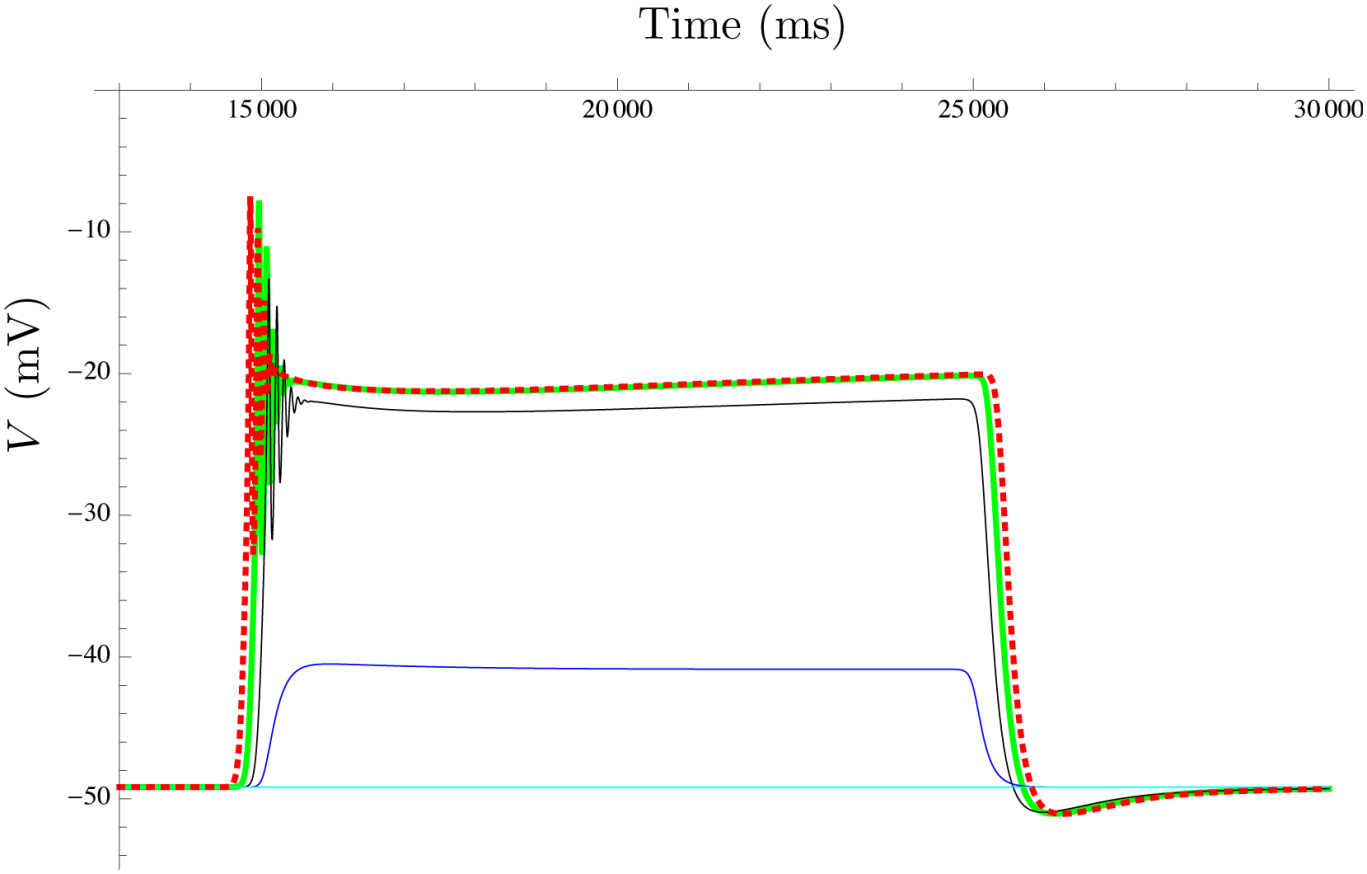
Effect of 5-BDBD on mechanical activity. (A) Representative trace of force generated by human myometrium at term, registered before and during application of 5-BDBD. (B) Reduction of contraction frequency associated with application of 5-BDBD (*n* = 11, means±SEM, *p* < 0.001).

To assess experimentally how P2RX4 might initiate spontaneous contractions, we recorded isometric tension from ∼5 × 10 mm muscle strips of the human myometrium, examining the effects of the P2RX4-antagonist 5-(3-Bromophenyl)-1,3-dihydro-2H-Benzofuro[3,2-e]-1,4-diazepin-2-one (5-BDBD) on contractile activity (see Materials and Methods section for details). Fig 10A shows a representative trace of the mechanical activity observed in term human myometrium, where application of 30 *μ*M 5-BDBD reduces contraction frequency to zero. The contraction frequency was significantly reduced from 0.2±0.09 to 0.01±0.01 contractions per minute, as shown in Fig 10B (*n* = 11, *p* ≤0.001).

#### Hypothesis II: Periodic depletion of PIP_2_

An alternative hypothesis to explain periodic depolarisation in MSMCs is that depletion of PIP_2_ affects several potassium channels. The second messenger precursor PIP_2_ is located in the inner leaflet of the cell plasma membrane where it participates in several signalling pathways, one of which produces the second messengers diacylglycerol and inositol-1,4,5-triphosphate [1]. Numerous plasma membrane ion channels require PIP_2_ to function and can be inhibited by signalling pathways that deplete PIP_2_ [54]. In particular, PIP_2_ regulates myometrial potassium channels such as Kir7.1, Kv7.1, Kv7.4, and hERG, and PIP_2_ could therefore well be the means by which the cell coordinates intracellular processes with gating of electrogenic proteins. In this way, periodic depletion of PIP_2_ could lead to depolarisation.

Lacking dose-response data on PIP_2_, we assumed a “two-level” mechanism: a release of PIP_2_ followed by a temporary depletion and then a replenishment of PIP_2_. We examined in detail the effect of PIP_2_ concentration on the kinetics of each of the ion channels affected. Two models, with different kinetics, were constructed for each of those channels, both with and without PIP_2_ application. We investigated the effect of PIP_2_ on each of the susceptible channels.

*Effect of PIP_2_ on hERG.* PIP_2_ modulates hERG by increasing its maximal current, concomitantly slowing deactivation, and changing activation and inactivation gating; at low PIP_2_ levels, a marginal hERG current persists [55]. A Markov model, adopted from previously described hERG models (see Materials and Methods) was used to simulate the effect of PIP_2_ on hERG [55]. Inactivation was modeled as a late activation-dependent transition. All transition rates were voltage-dependent. Model equations and parameters are given in the S1 Equations.

**Fig 11:**
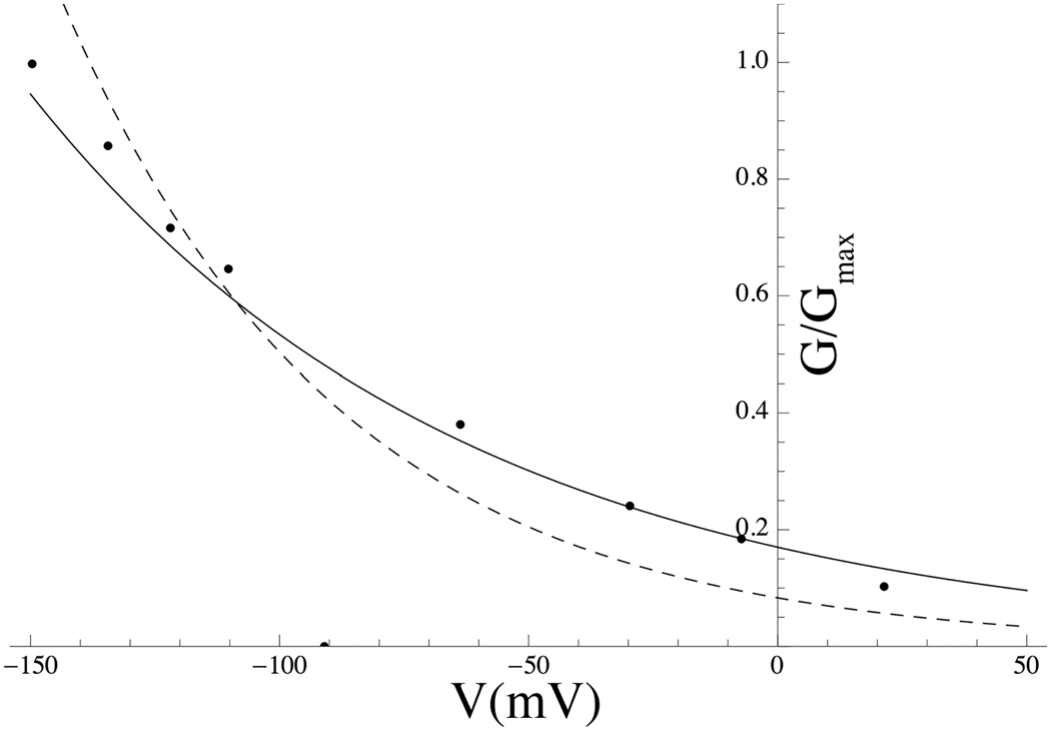
Effect of PIP_2_ on the Kir7.1 *G*-*V* curve. Simulated PIP_2_ effect on *G*-*V* curve (solid line) with data extracted from experiments by Pattnaik et al [56] (solid circles). The *G*-*V* curve in the absence of PIP_2_ (dashed line) is shown for comparison.

*Effect of PIP_2_ on Kir7.1* Members of Kir channel family were among the first channels demonstrated to be gated by PIP_2_ [54]. In general, Kir channels run down if PIP_2_ is depleted and reactivate if PIP_2_ is applied. Pattnaik et al [56] recorded whole-cell currents in solitary bovine RPE cells and isolated the Kir7.1 current as the Cs^+^-sensitive component. We extracted the *G*-*V* curve from the Cs^+^-sensitive *I*–*V* curve in a cell dialysed with solution containing 50 *μ*M PIP_2_ from Pattnaik et al [56] (Fig 11; equations are given in S1 Equations).

*Effect of PIP_2_ on Kv7.1 and Kv7.4.* PIP_2_ controls the activity of the KCNQ1/KCNE1 potassium channel complex; the current is suppressed by depletion of PIP_2_ [57]. However, we were unable to locate experimental data in the literature to support an effect of PIP_2_ on Kv7.1 and Kv7.4.

*No support for hypothesis II.* In free-running simulations using the conductance repertoire consistent with the smallest ℓ_1_-norm, the switch was applied for 30 seconds. Corresponding changes in the kinetics of the above ion channels were induced but failed to trigger an action potential.

#### Hypothesis III: Effect of calcium on chloride conductance

Intermittent release of calcium ions from intracellular stores gives rise to short-lasting local elevations of [Ca^2+^]*_i_*, known as ‘puffs’ or ‘sparks’ [58]. We surmised that these fluctuations drive a spontaneous transient inward current (STIC) which is carried by calcium-activated chloride channels (CaCCs). The activation of CaCCs depolarises the membrane slightly, increasing excitability and leading to the opening of L-type calcium channels, which in turn depolarises the membrane further. Further evidence for the involvement of CaCCs was provided by Wray et al [59], who showed that the pharmacological inhibition of CaCCs reduced the frequency of spontaneous contractions, although only ∼30% of MSMCs expressed these channels, which suggests that only cells within this subgroup have the potential to be pacemakers [59]. The CaCCs ANO1 and ANO2 are both present in human and murine myometrium and application of the antagonist tannic acid inhibits both STICs and MSMC contraction [60].

Local calcium sparks have been demonstrated in cardiac [61], skeletal [62, 63], and smooth muscle [64]. A single calcium spark is capable of producing a substantial local Ca^2+^ increase (10–100 *μ*M) [58], which barely affects the overall cellular calcium concentration (≤2 nM increase) [58]. The duration of a single spark is ∼100–450 ms [58, 65] and the same is true for STICs since the decay of the STIC mirrors the decay of the spark. The time interval between sparks varies from 200 ms to 1200 ms [58, 60, 66] and the frequency is between one to three sparks per second when cAMP or cGMP are elevated [66]. Each spark will activate ∼600 CaCCs, and the activation of ∼5% of the CaCC channels produces STIC [58]. Thus, the duration, amplitude, and frequency of the sparks, as well as the density of the CaCCs, could be central factors governing spontaneous contractions in MSMCs.

**Fig 12:**
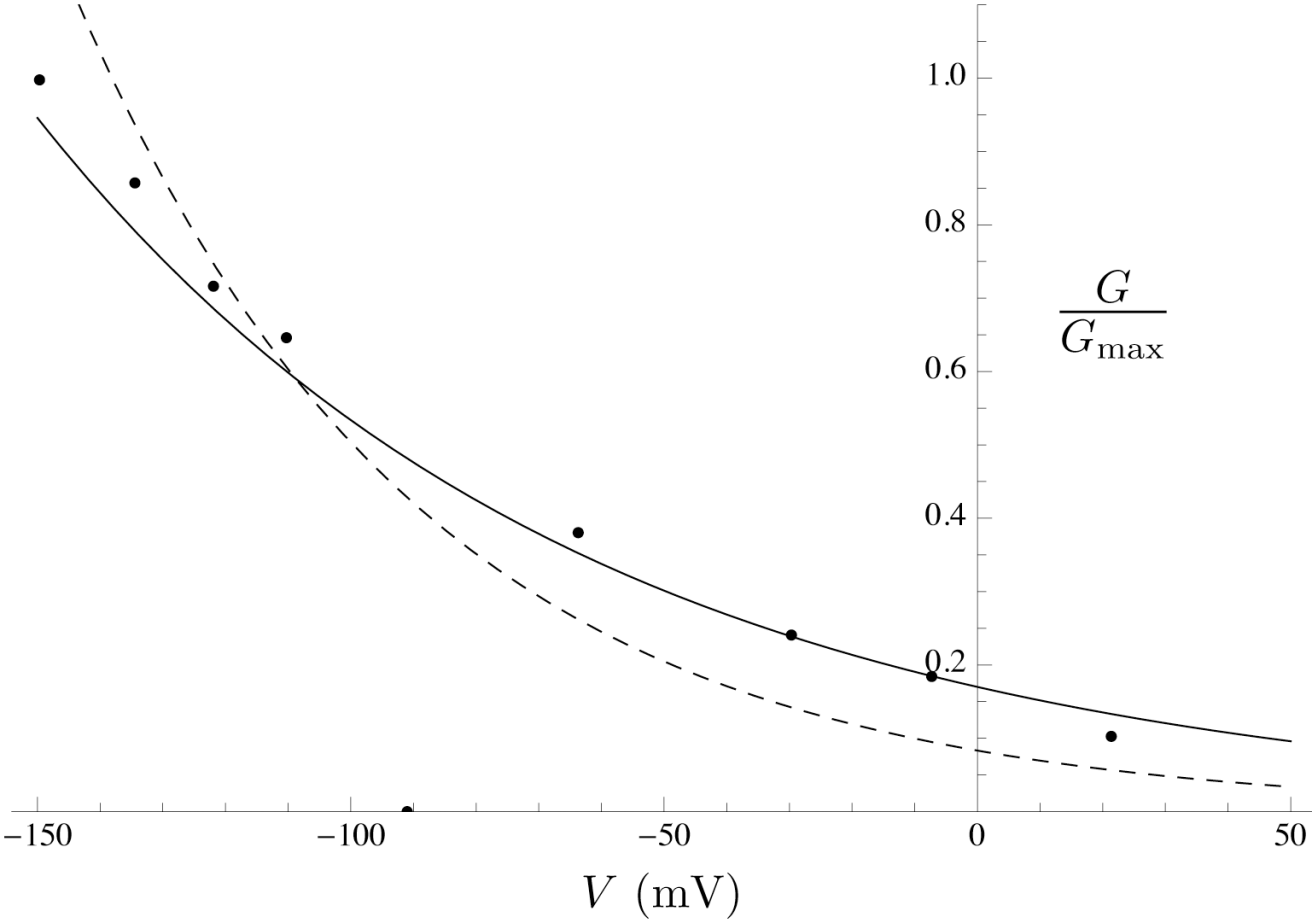
Simulations in support of Hypothesis III. (A) Simulated local calcium sparks; the panel to the right is an expanded time-axis representation of the boxed section (i). (B) Train of action potentials in response to driving the gating kinetics of a small proportion (5%) of the CaCC population with the simulated calcium sparks on top of the global cytosolic calcium concentration.

We investigated the putative role of CaCCs in MSMCs by representing the opening events of IP_3_-operated channels as a stochastic process [59] (as described in Materials and Methods). This process was used to drive the gating kinetics of a sub-population of CaCCs. Simulations in free-running mode revealed that a train of APs could be elicited when we imposed a succession of random sparks with duration between 200 ms to 500 ms, an amplitude of 10 to 80 *μ*M, and an average frequency around one spark per second (Fig 12). Spark amplitudes lower than 10 *μ*M did not trigger a full AP, while increasing the amplitude of the sparks beyond 10 *μ*M did not result in a significant variation in AP shapes. A density of at least 800 CaCCs per cell was required to elicit excitation. The duration and frequency of the AP train mirrored that of the generated sparks.

### Model simulations supporting experimental design

**Fig 13:**
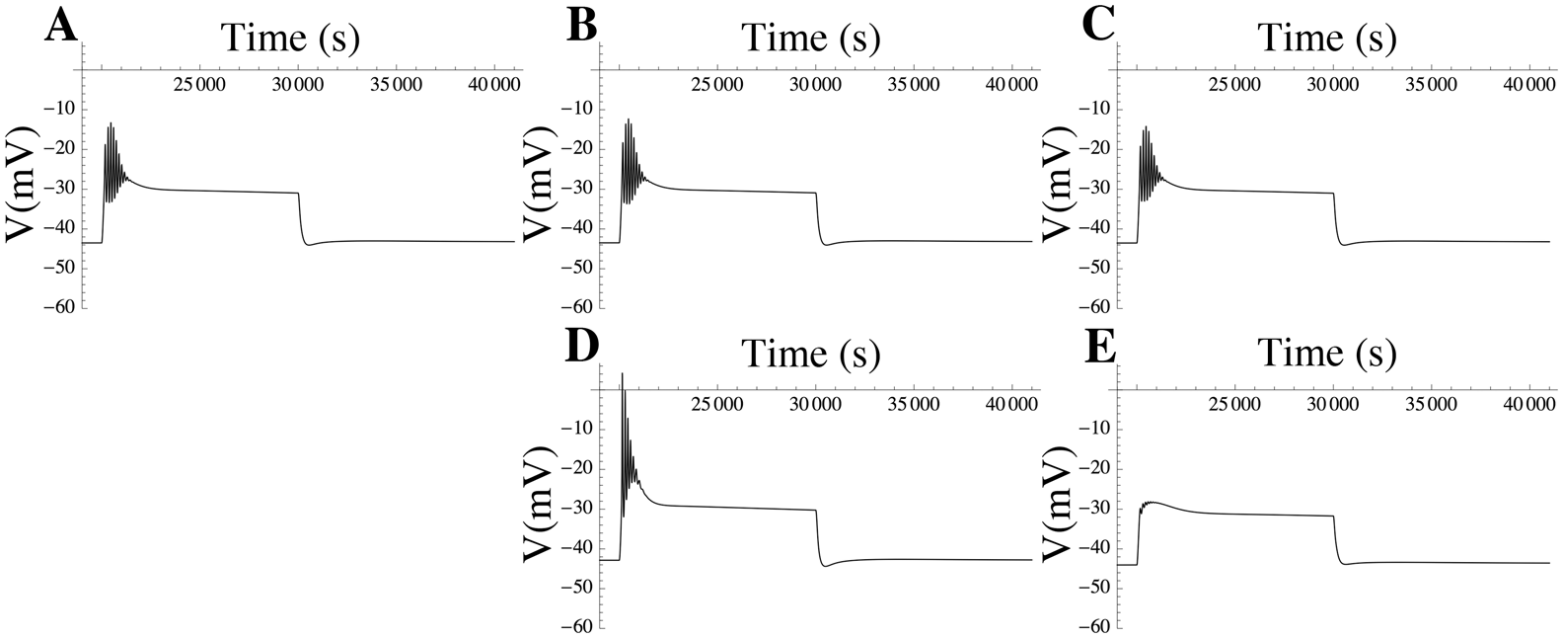
Model simulations in free-running mode. (A) Action potential triggered by a 10-sec extracellular ATP step function from 0 to 15 *μ*M with parameter values constrained by the ℓ_1_-norm. (B) Effect of setting the channel densities of BK and its isoforms to zero. (C) Effect of doubling the channel densities of BK and its isoforms. (D) Effect of setting the channel density of the Kv2.1 channel to zero. (E) Effect of doubling the channel density of Kv2.1.

Using free-running mode simulations in which the cell was activated through ATP variation (as outlined above), we evaluated the effect of increasing or decreasing various channel densities. The results are shown in Fig 13. When the BK channel density was set to zero, no significant effect was observed on the waveform, in accordance with our earlier characterisation of BK as functionally redundant. On the other hand, variation of Kv2.1 density, from around 30 channels/pF (double the estimated channel density of Kv2.1) to 0 channels/pF (complete block of Kv2.1), had a substantial effect on the waveform (Fig 13). When Kv2.1 was completely blocked, there was a more marked overshoot of the AP that rapidly depolarised to a plateau level with increased AP frequency. The slight change in the resting membrane potential (RMP) might imply a minor role for Kv2.1 in maintaining the RMP.

**Fig 14:**
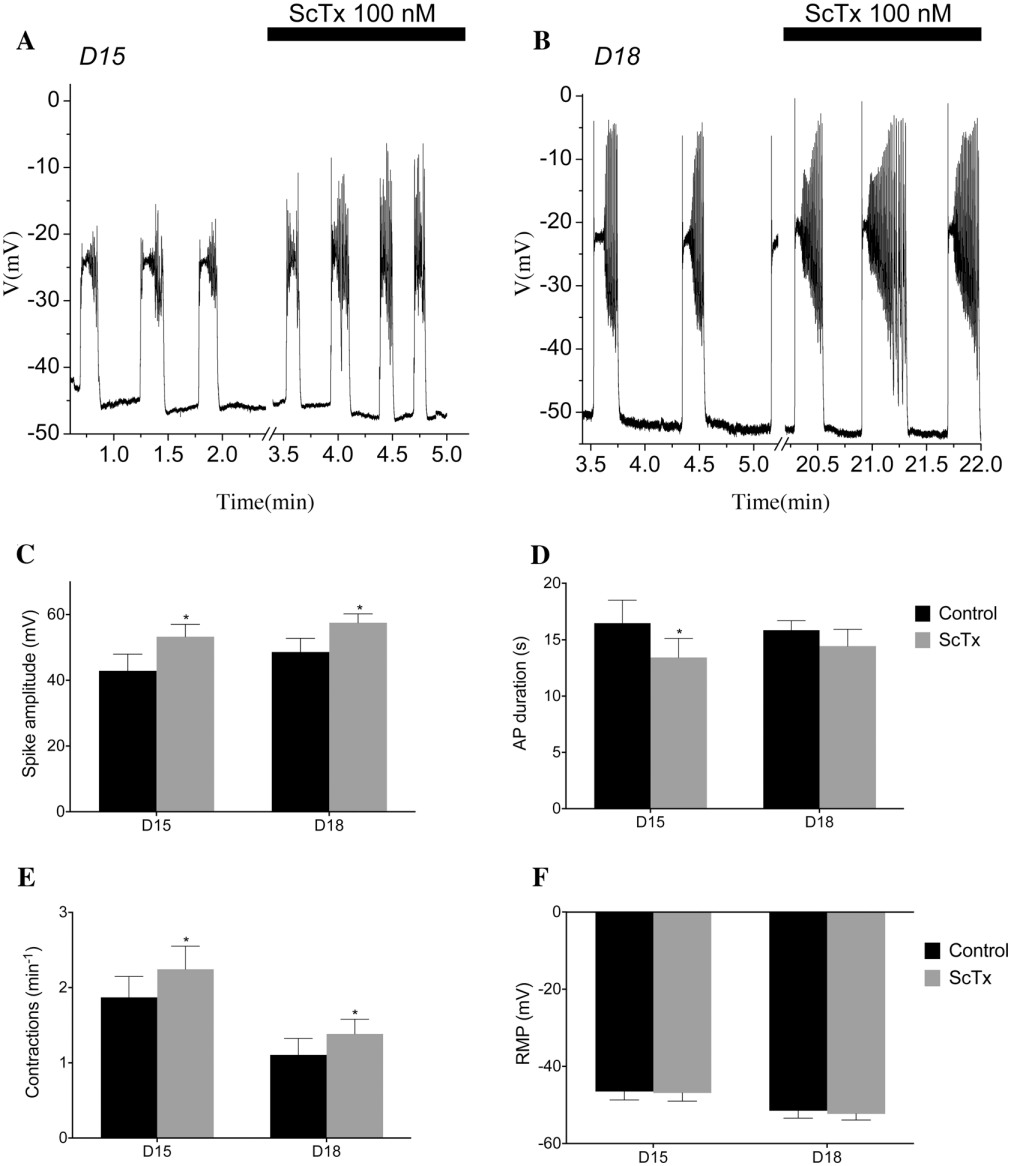
ScTx modulates AP frequency, duration, and spike amplitude. Spontaneous electrical activity recorded from the longitudinal layer of D15 & D18 murine myometrium (A, B). Activity consisted of slow depolarisation to threshold, followed by an action potential composed of a plateau upon which a number of spikes were superimposed. The bar represents the maximum effect in the presence of 100 nM ScTx. (C) Mean AP spike amplitude increase from 42.87±5.07 mV to 53.25±3.78 mV (*p* ≤ 0.05) in D15 tissue (*n* = 6) and from 45.5±4.96 mV to 56.21±3.37 mV in D18 tissue (*n* = 6). (D) Decrease in mean AP duration from 16.77±2.14 s to 11.87±0.76 s (*p* ≤ 0.05) in D15 tissue but no significant difference in D18 tissue. (E) Application of 100 nM of ScTx increased AP frequency in the D15 samples from a mean of 1.69±0.18 to 2.31±0.34 AP min^−1^ and from 1.11±0.22 to 1.38±0.19 AP min^−1^ in D18 tissue. (F) ScTx did not alter the resting membrane potential of either D15 or D18 (*p* ≤ 0.01).

**Fig 15:**
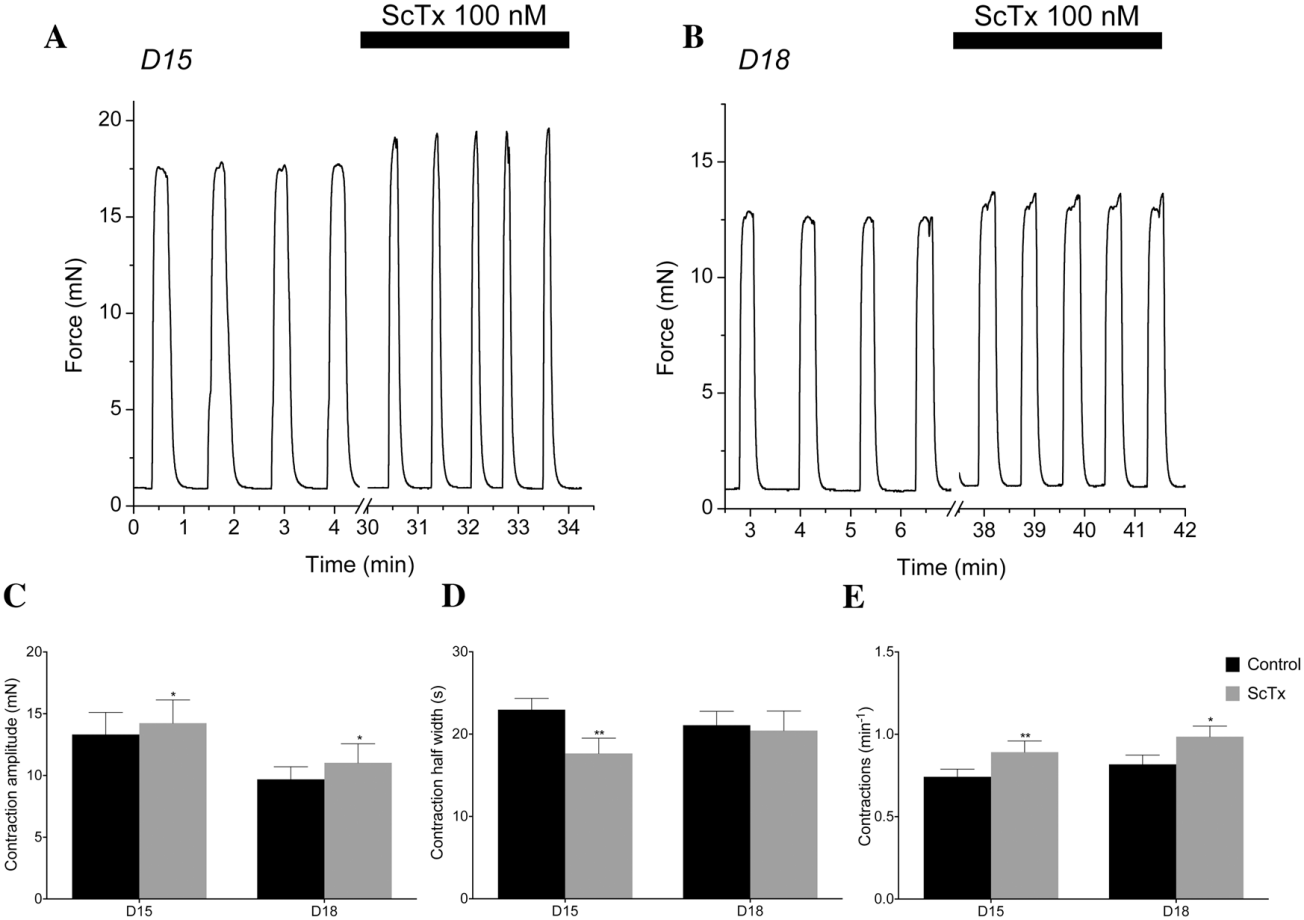
Kv2.1 contribution to spontaneous contractile activity. (A, B) Representative isometric force recordings in the presence of 100 nM ScTx in D15 (A) and D18 (B). (C) ScTx significantly increases contraction amplitude in both D15 murine myometrium from 13.32±1.79 mN to 14.24±1.87 mN and in D18 from 9.69±1.79 mN to 11.02 ± 1.87 mN. (D) ScTx significantly decreases contraction half width in D15 from 22.97±1.38s to 17.65±1.86s, while contraction half width in D18 tissue remained unchanged. (E) Effect of ScTx on contraction frequency for D15 & D18 murine myometrium. ScTx significantly increased contraction frequency for D15 & D18 murine myometrium from 0.74±0.05 to 0.89±0.07 contractions per minute and in D18 from 0.82±0.06 to 0.99±0.07 contractions per minute (*p* ≤0.01, *n* = 7).

In order to determine what role Kv2.1 currents might play in the whole tissue and to test the predictions of our model, we examined ∼3 × 10 mm muscle strips from the longitudinal layer of murine myometrium under current-clamp conditions using a sharp microelectrode to record fluctuations in membrane potential over time. Typical examples of the activity observed in the myometrial muscle strips are shown in Fig 14A and 14B, where we observe a spontaneous AP generated from a RMPs of −45 mV to −50 mV under control conditions for both Day 15 (D15) and Day-18 (D18), respectively. The application of 100 nM ScTx elicits an increase in AP spike amplitude in the D15 sample and also in the D18 sample (albeit to a lesser extent). The summary data (*n* = 4 and *n* = 6 for D15 and D18, respectively, p ≤ 0.05) for these experiments are presented in the graphs in Fig 14C, 14D, 14E and 14F, where mean AP spike amplitude was significantly increased from 42.87±5.07 mV to 53.25±3.78 mV in D15 and from 45.5±4.96 mV to 56.21±3.37 mV (*p* ≤ 0.05) in D18 tissue. Application of 100 nM ScTx also significantly increased AP frequency in D15 samples from a mean of 1.69±0.18 AP min^−1^ to 2.31±0.34 AP min^−1^ and 1.11±0.22 AP min^−1^ to 1.38±0.19 AP min^−1^. A modest yet consistent reduction in the duration of AP was apparent in APs recorded from D15 tissue. The number of experiments carried out in the current study was insufficient to detect a significant change in RMP with ScTx application in either D15 or D18 cells (Fig 14F). To assess how Kv2.1 regulates spontaneous contractions, we recorded isometric tension from ∼3 × 10 mm muscle strips of the longitudinal layer of the mouse myometrium, examining the effects of 100 nM ScTx on the activity. Fig 15A and 15B show representative traces of the mechanical activity of D15 and D18 myometrium. In the presence of 100 nM ScTx, contraction amplitude was increased (13.32±1.79 to 14.24±1.87 mN, D15; 9.69±1.79 to 11.02±1.87 mN, D18; Fig 15C), as was frequency (0.74±0.05 to 0.89±0.07 contractions per minute, D15; 0.82±0.06 to 0.99±0.07 contractions per minute, D18; Fig 15E), whereas contraction half-width was reduced (22.97±1.38 to 17.65±1.86 s, D15; not significant in D18; Fig 15D).

## Discussion

Laser-capture microdissection [67] and mRNA-sequencing have led to significant advances in gene expression analysis in both single cells and tissues. Application of these techniques provided a complete repertoire of the mRNA population in MSMCs, which we used to construct a list of possible electrogenic entities by combining all possible combinations of ion channel subunits that are potentially expressed in the MSMC. Since many channels exist as various combinations of subunits, we obtain a repertoire of over thirty electrogenic entities that could be present in the plasma membrane of the MSMC. A mathematical model for every individual potential oligomeric channel complex was formulated on the basis of biophysical data in the literature (typically obtained via heterologous expression).

We used native cell behavior to drive the individual conductance currents (viz. voltage and calcium time series as detailed in Materials and Methods). The remaining unknowns were the densities in the plasma membrane of the various species. The observed membrane potential time series is linear in these densities, allowing us to calculate the space of all possible solutions of ion channel densities that are consistent with the observed data; the functional redundancy of the system is represented by the basis vectors of this space. To fix the channel densities we considered the most parsimonious solution defined as having the smallest ℓ_1_-norm subject to non-negativity. Alternative criteria are possible, such as optimising for agreement with mRNA expression levels for the conductances. However, this approach is conditional on accurate quantitative measurements of expression levels, and on the availability of a reliable mapping from transcriptomics to functional proteomics; neither condition is satisfactorily met at the present state of the art (life times and translation efficiencies of mRNA species vary; moreover, the protein may be stored or degraded instead of being translocated to the membrane).

Predictions were made using our model based on the conductance repertoire imposed by the criterion of the smallest ℓ_1_-norm. These predictions were tested against experimental data, obtained in voltage-clamp experiments for isolated currents that were sensitive to inhibition by antagonist application. The simulated values of peak currents in response to voltage steps closely matched the values that were observed experimentally. In the case of certain channels, for example Kir7.1, the simulated data failed to predict the observed data accurately, which can be attributed to the fact the particular voltage waveform that was used to drive the model does not distinguish well between currents (e.g. between the Kir7.1 current and the background potassium current).

The myometrium has been modeled at the levels of both tissue and the whole organ [5–7]. Bursztyn et al developed an excitation-contraction model of the uterine muscle cells, comprising voltage-gated calcium channels, calcium transporters, and Na^+^/Ca^2+^ exchangers [8]. This model explicitly accounts for the processes of myosin light chain phosphorylation and stress production as per the cross-bridge model of Hai and Murphy [9], but did not include the inactivation process of the calcium current and several other ionic currents. By contrast, Rihana et al [10] modeled the *macroscopic* currents for each of the relevant ionic species based on voltage-clamp experiments; the dimension of the state space of this model was an order of magnitude lower than ours. Nevertheless, they were able to reproduce various phenomena, such as a single AP, or a train of APs with an RMP of −35 mV to −40 mV and a duration of about 133–200 ms. Another macroscopic ionic current model, accounting for fourteen types of current, was presented by Tong et al [4]. Gating kinetics in the present study were largely derived from equations and parameter estimates taken from the literature and as such representative of the state of the art in the field; accordingly, not all models may have been evaluated to the same standard of rigour as advocated by Fink and Noble [68].

Huys et al [69] proposed an approach similar to ours. However, they illustrated their method on simulated data generated by a macroscopic current model, as opposed to the present work in which real-life data have been confronted with a microscopically detailed account of all relevant currents. One distinguishing feature of our approach is that we can chart the functional redundancy of the system at the level of individual species of electrogenic entities. The redundancy maps show which combinations of channels can be substituted for one another. We investigated the potential scope for redundancy of the system of some of the most-studied potassium channels in MSMCs (e.g. BK, Kv2.1, SK3, hERG).

The use of singular-value decomposition (SVD) to determine the directions of substantial variation in parameter space is well-established [70]. A key advantage of SVD over a naive one-factor-at-a-time approach [71], is that the spectrum of singular values expresses directly which linear combinations of parameters strongly affect the read-out of interest. Sher et al. [72] applied the technique to ionic channel modelling and used the SVD of the sensitivity matrix collecting the partial derivatives of the read-out quantities of interest with respect to the model parameters to probe identifiability and sensitivity, whereas the present method takes a single quantity of interest, the voltage, but treats its *entire time course* as the output and is accordingly time-global. A regression-based method to perform parameter sensitivity analysis in electrophysiological models was proposed by Sobie [73], who derived quasi-equivalent linear models by treating simulation outputs obtained under randomised parameter variation as statistical input data. Saltelli and Annoni [71] emphasize the essential locality of “one-at-a-time exploration” given the lack of information that would be provided by the higher-order derivatives (including cross terms), but this caveat is not applicable here since the model is linear in the vector of parameters of interest (our model is non-linear in the parameters that were fixed from extraneous data but this is not germane to the point made by these latter authors).

The role of BK channels in modulating uterine excitability remains controversial: whilst the presence of the protein and an active conductance are not disputed [74–76], the impact of the BK current is not fully understood [40]. Our functional redundancy analysis indicates that the effect of BK can be compensated by small changes in the channel densities of the other channels in the model. This suggests that the effect which BK exerts on the voltage waveform (as observed in the present study) is readily replaceable by the currents carried by other electrogenic entities. This observation is consistent with the findings of Aaronson et al, who demonstrated that application of BK inhibitors has no effect on spontaneously contracting rodent myometrial strips [40]. If BK can be readily substituted by suitable combinations of other channels, one might ask why it should be part of the conductance repertoire at all. It may be relevant that the unitary conductance of the BK channel is unusually large [77], which means that the cell can be silenced by expressing even a minor number of these molecules. Perhaps BK serves the function of an “emergency shut-down.”

We also investigated the potential involvement of Kv2.1 in shaping the action potential (AP). We investigated the effect of changes in Kv2.1 channel density on the AP waveform by running free-running simulations. Decreasing the Kv2.1 channel density from the estimated value to zero increased the amplitude of the initial AP as well as the rate at which steady state is reached during the plateau phase of the AP. The predicted effects on AP amplitude and frequency were confirmed by experiments although the effect on the resting membrane potential (RMP) could not be verified with certitude. It appears that Kv2.1 channels do play a role in modulating the excitability of the myometrium by inhibiting both contraction frequency and amplitude, while contributing little to the RMP.

As regards the hERG channel, our functional redundancy analysis indicated that small changes in the hERG channel density could not be compensated by physiologically realistic adjustments in the densities of the other channels. In addition, the simulations carried out free-running mode suggest that variations in the hERG channel density substantially affect the AP waveform (Fig 13). Taken together, these findings indicate that hERG is a major suppressor of bursting activity in MSMCs, in keeping with Greenwood et al [41], who observed that the ERG-specific blocker dofetilide-induced contractions in quiescent tissues, while ERG-channel blockers were able to induce contractions in tissue strips that failed to develop spontaneous rhythmic activity.

In contrast to hERG’s essential role, the SK channels were found to be readily susceptible to compensation. The redundancy analysis indicates that SK_2_ can be functionally replaced by adjustments in SK_3_ and SK_4_ densities. This is in accordance with the close physiological similarities between the members of the SK family.

We also demonstrated the utility of the model in exploring various mechanistic hypotheses, addressing three mechanisms that could trigger spontaneous contractions. Simulations provided supporting evidence for the involvement of extracellular ATP and/or calcium ‘sparks’ affecting CaCCs, whereas we found no support for a role of PIP_2_ acting on potassium channels. Although far from conclusive in their own right, such simulations can help experimentalists to pinpoint promising research avenues.

The model can be improved in several ways. In its present form, the dynamics of intracellular calcium are represented in a minimalistic way and a more physiologically faithful model would be desirable. However, this is subject to the quality of the temporospatial resolution of the cytosolic and sarcoplastic-reticular levels of Ca^2+^. Another mechanism we have not considered here is the feedback from force-generation on the dynamics of membrane potential, which acts via stretch-sensitive ion channels. Furthermore, our model describes a single cell, whereas the *in vivo* activity of MSMCs is critically dependent on their interconnectivity. Thus, a multi-cell model with realistic network properties would constitute another future improvement.

In the present study a spontaneous voltage signal was imposed. It is also possible to impose artificial voltage waveforms (AVWs), for instance composed as a linear combination of B-splines, and compare the current response to such an AVW to the current response of the model. The advantage of this approach would be that the AVW can be optimised to discriminate maximally between two given conductances, for instance by minimising the inner product between the current responses of the respective conductances, where the two are considered as functions of time. A tighter upper bound on the functional redundancy of the system can be obtained in this manner.

The functional redundancy we map is strictly relative to the waveform that is imposed. As more behaviors are explored, more “essential roles” are revealed and the dimensionality of the kernel can generically be expected to diminish; in fact, it could come down all the way to zero, which would imply that there is no intrinsic redundancy in the conductance repertoire. Alternatively, the kernel dimensionality settles on a non-zero value “in the limit of arbitrarily many explored behaviors”—this value would then denote the irreducible functional redundancy of the system. It stands to reason that sundry physiological limitations will impose further constraints on the system, but the considerations in the present paper concern only the functional redundancy vis-à-vis the membrane conductance repertoire.

Two key advantages of our approach are that it is *modular* and *expandable*. The model can readily accommodate new information regarding additional conductances or novel kinetic properties. Moreover, being expressed in terms of microscopic conductances that correspond to particular molecular entities, the model can in principle be related directly to transcriptomics. This has important practical advantages, because transcriptomics data can be acquired in different tissues, and at different time points, accurately surveying all potential molecular entities. Another facet of the expandability of our approach is that the characterisation of functional redundancy, becomes more accurate, that is, comes closer to the irreducible functional redundancy, as a wider range of physiological behavior of a given cell (both spontaneous and evoked) is observed.

Our analysis of functional redundancy provides a means to address the well-known difficulty in electrophysiological modelling that electrophysiology at the whole-cell level is insufficient to constrain the densities of all channels. Traditionally, this problem has been solved by postulating macroscopic currents that represent the combined action of several similar conductances, but this approach has the drawback that a direct correspondence to genomic or transcriptomic data is lost. We would even argue that the approach based on macroscopic currents is a false economy since, from an experimental point of view, the acquisition of the macroscopic currents is itself underdetermined, whereas microscopic currents can be unequivocally isolated and thereby fully characterised.

## Materials and Methods

### Ethics statement

All procedures were conducted within the guidelines of *The Declaration of Helsinki* and were subject to local ethical approval (REC-05/Q2802/107). Prior to surgery, informed written consent for sample collection was obtained.

### Experimental methods

#### Solutions

Fresh samples were stored in ice-cold modified Krebs-Henseleit (m-KHS) solution containing (mmol l^−1^): NaCl 133; KCl 4.7; Tes 10; glucose 11.1; MgSO_4_ 1.2; KH_2_ PO_4_ 1.2; CaCl_2_ 2.5; adjusted to pH 7.4 at 25°C with 5 NaOH. Hank’s Balanced Salt Solution (HBSS) containing no calcium or magnesium was purchased from Invitrogen (Paisley, UK). The electrode (internal) solution for voltage clamp studies contained (mmol l^−1^): KCL 140; EGTA 1.1; CaCl_2_ 0.06; Hepes 10; MgCl_2_ 2; adjusted to pH 7.2 at 25°C with 5 NaOH. 5-(3-Bromophenyl)-1,3-dihydro-2H-Benzofuro[3,2-e]-1,4-diazepin-2-one (5-BDBD) stock solution was prepared in DMSO (Tocris Bioscience, Bristol, UK). ScTx stock solution was prepared in HBSS (Alomone Labs, Jerusalem, Israel).

#### Subject criteria and sample collection

*Subject criteria.* Samples were taken at caesarean section at term (>37 and <40 completed weeks gestation).

*Sample collection.* At caesarean section, human tissue samples were collected before syntocin administration by knife biopsy from the lower uterine segment incision. Samples for cell isolation were placed in ice-cold modified Krebs-Henseleit solution and utilised the same day. Experiments were also carried out on murine whole tissue or single cells taken from day of gestation 15 (D15) and 18 (D18) pregnant mice (B6 CB F1), culled by CO_2_ asphyxiation. The uterine horn was dissected to remove the pups and the myometrium was stored in ice-cold m-KHS.

*Cell isolation.* Strips of myometrium from the longitudinal layer (2 × 2 × 20 mm) were isolated and washed in Ca^2+^- and Mg^2+^-free HBSS at 37°C for 10, 20, and 30 minutes, respectively, followed by 45 min incubation in digestion solution containing Liberase^™^ (Roche, Welwyn Garden City, UK) dissolved in HBSS to a final concentration of 0.13 WU/ml at 37°C. Digestion was terminated by several dilutions with fresh HBSS. Cells were dispersed by slow trituration through a wide-bore fire-polished glass pipette in HBSS Solution. Single myometrial cells were filtered through a 200 *μ*M gauze and stored in HBSS for use within 6 hours

#### Electrophysiology and imaging

*Voltage clamp.* A drop of myometrial cell suspension was placed in a glass-bottomed petri dish and mounted on the stage of an inverted microscope (IX51, Olympus). After settling (approx. 10 min), cells were perfused with bath solution at a rate of 1–2 ml min^−1^ at 37°C. Patch pipettes were fabricated (Model P-87; Sutter Instruments, Novato, CA, USA) from 1.5 mm glass capillaries with a resistance of 2.0–4.0 MΩ when filled with pipette solution. Liquid junction potential was zeroed prior to seal formation. Transmembrane currents were recorded with an amplifier (Axopatch 700b; Axon Instruments) using the perforated-patch configuration of the whole-cell patch-clamp technique [78]. The cell membrane was perforated using the antibiotic amphotericin B (720 *μ*g/ml). Series resistance was compensated after membrane perforation. Currents were elicited by stepping to a range of potentials between −150 and +80 mV from a holding potential of −60 mV. To isolate currents that were sensitive to inhibition by drug application, difference currents were obtained by electronic subtraction of traces. Currents were filtered at 10 kHz and sampled at 5 kHz. Voltage protocols were delivered via a Digidata 1440a computer interface using *pCLAMP 9.0* software (Molecular Devices, Sunnyvale, CA, USA).

*Ca^2+^ imaging and current clamp recordings of isolated cells* (*recordings in* S1 Data) Smooth muscle cells were impaled with glass microelectrodes filled with 2M KCl of resistance 80–120 MΩ. Transmembrane potentials were recorded at an acquisition rate of 1 KHz with an amplifier (Axopatch 700b; Axon Instruments) and a Digidata 1440a computer interface using *pCLAMP 9.0* software (Molecular Devices, Sunnyvale, CA, USA).

Single myometrial smooth muscle cells were plated onto glass-bottomed, collagen-coated dishes (Matek, Massachusetts, USA) and left to adhere for 10 min on an Olympus IX51 microscope stage. Cells were incubated in the dark for 60 min at room temperature with the Ca^2+^-sensitive fluorescent dye Fluo4-AM (5 *μ*M, Invitrogen, Paisley, UK), Pluronic F127 (0.025%, w/v) was included to aid dye loading. Cells were washed then maintained at 37°C in m-KHS. Fluo-4 was excited by an X-cite 120 UV lamp (EXFO, Eastleigh, UK) and emitted light (520 nm) was captured with a Luca^EM^ EMCCD camera, which was controlled by the *IQ* software package (Andor Technology, Belfast, UK). Images were acquired at 30 Hz.

Mean camera background was subtracted and images were normalised to obtain f/f_0_ by dividing the entire image by the mean intensity of the cell during a quiescent period, defined as the minimum fluorescence obtained between periods of excitation.

*Current clamp recordings of myometrial strips.* Strips (5 × 10 mm) of murine myometrium from the longitudinal layer were pinned out on a sylguard base and perfused with m-KHS solution at 37°C on an upright microscope (MVX10, Olympus). Tissue was incubated with 5 *μ*M wortmannin (Sigma) to prevent spontaneous contractions from dislodging impalements. Smooth muscle cells were impaled with glass microelectrodes filled with 2M KCl, of resistance 80–120 MΩ. Transmembrane potentials were recorded with an amplifier (Axopatch 700b; Axon Instruments) and a Digidata 1440a computer interface running *pCLAMP 9.0* software (Molecular Devices, Sunnyvale, CA, USA).

#### Isometric tension recordings

*Murine myometrium.* Strips of myometrium measuring approximately 2 × 2 × 10 mm were cut the longitudinal layer of D15 and D18 uterine horns. Each strip was mounted horizontally on a muscle strip myograph system (800MS, Danish MyoTechnology, Denmark) placed under 2 mN tension. Strips were bathed in 4 ml of m-KHS at 37° which was replaced every 30 minutes. After an initial equilibration period, isometric force was recorded using *LABCHART* software (ADI Instruments, Oxford, UK).

*Human myometrium.* Myometrial muscle strips approximately 10 × 2 × 2 mm were mounted vertically in 10 ml organ bath chamber perfused in m-KHS at 37°C. Force was measured with FT03C transducers (Grass Instrument Co, Quincy, Mas) and recorded digitally with *MacLab Chart* software (ADInstruments Ltd, Oxfordshire, UK). Strips were held under 20 mN tension and allowed to equilibrate for 90–120 min, wherein spontaneous contractions were observed. Strips that failed to contract spontaneously were excluded. 5-BDBD was added directly to the organ bath chamber.

#### Computational methods

Data were extracted from published graphs by means of the graph digitiser software package *GraphClick*. Least-squares estimation was used to estimate parameter values. The equilibrium point of the dynamics without input current was used as initial condition. All analyses were carried out in *Mathematica* (Wolfram Research, Champaign, IL), using the built-in facilities for the numerical evaluation of differential equations, least-squares estimation, and singular-value decomposition. Code is listed in S1 and S2 Models. Briefly, the *Mathematica* function NDSolve was used to solve ordinary differential equations, using an adaptive method. The default method, Automatic, automatically switches between backward differentiation formulas and Adams multistep methods, depending on the stiffness. In *Mathematica*, FindFit was used to perform least-squares parameter estimation, SingularValueDecomposition to obtain singular value decompositions, and LinearProgramming to solve linear programming problems. All statistical analyses were carried out using GraphpadPrism (California, USA). Data are presented as mean ± standard error of the mean, with *n* referring to the number of cells. Statistical significance two-tailed paired or unpaired student *t*-test where relevant. Significance is indicated at levels of 0.05 (*), 0.01 (**), or 0.005 (***).

The channel-density method is based on the well-established current summation framework:

$$C\frac{d}{dt}V=I\left(t\right)-\sum_{i=1}^{n}\varkappa_{i}\psi_{i}\left(t\right)$$ (3)

(this is eq (1), repeated here for the sake of convenience). The unit currents *ψ*_*i*_ are generally described by the following standard equation:

$$\psi_{i}=g_{i}\left(x\right)\left(V-E_{i}\right)$$ (4)

where *g*_*i*_ is unit conductance, dependent on gating variables collected in a vector *x*, and *E*_*i*_ is the reversal potential of the *i*th conductance. For every channel, there is a more or less complex model describing the gating kinetics. In abstract form, each of these can be represented as follows:

$$\frac{d}{dt}x=f\left(x,V\left(t\right),u\left(t\right)\right)\;.$$ (5)

Here ***u***(*t*) denotes any additional input variables, such as the intracellular calcium concentration and the extracellular ATP concentration; ***x*** collects the gating variables for all the conductances, transporters, and exchangers in the model, and ***f*** represents the dynamics of these variables. In our model $x\in R^{123}$.

The experimentally observed time series for the membrane potential *V*(*t*), together with the calcium time series (treated as an input variable *u*(*t*)), are used as forcing functions to drive the gating kinetics, eq (5), in a numerical solution of the corresponding initial-value problem (IVP). In practice, we are dealing with a time series in which the membrane potential has been observed at times *t*_1_, *t*_2_, …, *t*_*m*_, where is can be arranged that *m* > *n*. Numerical solution of the IVP requires that we work with interpolated forms of the voltage and calcium waveforms; we ensured that the sampling density was sufficient that linear interpolation gave satisfying results not substantially different from higher-order interpolations. The numerical solution of the IVP yields the currents *ψ*_*i*_, which are in turn integrated numerically to give:

$$q_{i}\left(t_{k}\right)=\int_{0}^{t_{k}}\psi_{i}\left(\tau \right)d\tau$$ (6)

at times *t*_*k*_ with 1 ≤ *k* ≤ *m*. We can represent the basic eq (3) as an integral equation

$$V\left(t_{k}\right)=V\left(0\right)+V_{0}\left(t_{k}\right)-\sum_{i=1}^{n}\frac{\varkappa_{i}}{C}q_{i}\left(t_{k}\right)$$ (7)

where the voltage term due to injected current is given by $V_{0}(t_{k})=C^{-1}\int_{0}^{t_{k}}I(\tau )d\tau$. The integral form, eq (7), is intrinsically linear in the quantities *q*_*i*_ (i.e. not the result of a linearisation, hence global). We define scaled densities as follows: *κ_i_* = *ϰ_i_*/*C* for *i* = 1, …, *n*, which we collect collect in a vector $\kappa \in {R_{+}}^{n}$. This vector represents the conductance repertoire. Our objective is to find a value for the conductance repertoire ***κ*** that minimises the discrepancy between the driving time series *V*(*t*_*k*_) and the membrane potential calculated according to eq (7).

Next, we define ***W*** to be an *m* × *n* matrix such that *W*_*hi*_ = *q*_*i*_(*t*_*h*_) and ***Y*** to be an *m*-vector whose *h*th element is *V*(*t*_*h*_) − *V*(0) − *V*_0_(*t*). The channel densities, i.e. the elements of the conductance repertoire vector ***κ***, are constrained by the matrix equation

Y=W·κ. (8)

Even though this equation is readily solved in the least-squares sense, uniqueness is not ensured since the rank *r* of ***W*** may be smaller than *n*.

The space of least-squares solutions can be found via the singular-value decomposition ***W*** = ***U*** ⋅ Σ ⋅ ***V***^*T*^. Let ***V***_*r*_ denote the matrix containing the first *r* columns of the *n* × *n* matrix ***V***, ***D*** an *r* × *r* diagonal matrix containing the non-zero singular values, and ***U***_*r*_ the matrix containing the first *r* columns of the *m* × *m* matrix ***U***. Then the pseudo-inverse of ***W*** is given by $V_{r}\cdot D^{-1}\cdot U_{r}^{T}$ and a particular least-squares solution is given by the following expression:

$$\kappa^{+}=V_{r}\cdot D^{-1}\cdot U_{r}^{T}\cdot Y\;.$$ (9)

This describes the solution characterised by having minimal Euclidian norm. However, it is not necessarily the desired solution because we also require that the densities be non-negative. Otherwise the solution would have no physiological significance. The final *n* − *r* columns of ***V*** constitute an orthonormal basis for the null space of ***W***. Writing these as $v_{1}^{0}$, $v_{2}^{0}$, … $v_{n-r}^{0}$, we have the least-squares estimate for the scaled densities in the following general form:

$$\hat{\kappa }=\kappa^{+}+\sum_{j=1}^{n-r}\gamma_{j}v_{j}^{0}\;.$$ (10)

The coefficients *γ*_1_, *γ*_2_, …, *γ*_*n*−*r*_ remain to be determined, subject to constraint that *κ* lie in the positive cone. If we wish to single out one vector in the set of channel density vectors, as is necessary to employ the model in free-running mode simulations, further constraints must be imposed. In addition to non-negativity, we use the criterion of least total channel density. The coefficients *γ*_1_, …, *γ*_*n*−*r*_ that minimise the sum of the elements of $\hat{\kappa }$ (such that $\hat{\kappa }\in R_{+}^{n}$) can then be determined by means of linear programming. The resulting $\hat{\kappa }$ represents the expression profile that achieves the observed behavior with the least amount of molecular building blocks expended (a criterion that can be further refined by noting that ion channels do not all have the same molecular weight; and additional step taking this into account could have been included). We should perhaps emphasise that, due to the non-linearity of ***f***, the kernel is strictly local in the space of all possible waveforms; in particular, a reduction of the dimensionality of the kernel may generally be anticipated when the waveform probes the behavioral propensities of the system more extensively (with a slight technical modification, it is even possible to concatenate waveforms from a series of experiments) and in the limit of an arbitrarily extended exploration of the behavioral envelope of the system, the dimensionality of the kernel approaches the intrinsic functional redundancy of the system from above.

In practice, it was found that the affine space generated by eq (10) does not always intersect with the positive cone. Accordingly, a vector *κ*_*p*_ was found using constrained least-squares fitting that satisfies the non-negativity requirement, and this value was used instead of ***κ***^+^ in eq (10) to generate the most-parsimonious solution by means of linear programming. This modification does not affect the analysis of functional redundancy, since the basis of the kernel remains unaltered.

The conductance repertoire vector obtained by the above method was used for exploratory simulations that have been reported previously in McCloskey et al [39].

#### Physiological upper bounds on channel density values

There are natural physiological limitations to the surface density of ion channels in functional cells. To obtain a conservative estimate of these upper bounds (listed in Table 3), we attempted to calculate a reasonable estimate for the maximum possible channel density for each of the conductance species in the model, proceeding as follows. From eqs (3) and (4) we obtain the current carried by a species *i*:

$$I_{i}=\varkappa_{i}\;g_{i}\left(x\right)\left(V-E_{i}\right)\;.$$ (11)

**Fig 16:**
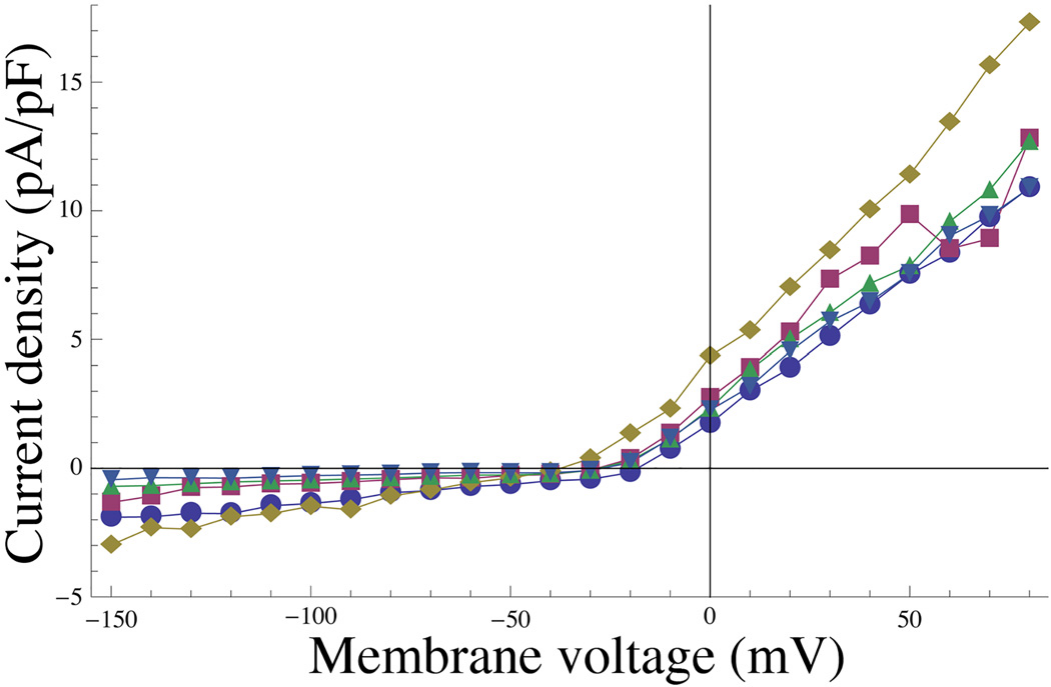
Experimental *I*–*V* relationships. Replicate *I*–*V* curves (*n* = 5 cells) measured under voltage-clamp conditions; values are averages over a 50 ms period, commencing 450 ms after applying a voltage step.

Using a set of whole-cell voltage-clamp *I*–*V* curves for MSMCs (a selection of which is shown in Fig 16), we obtain the steady-state current density $\bar{I}\left(V\right)$ for a given MSMC. Substituting in Eq (11), we find:

$$\bar{I}\left(V\right)=\varkappa_{i}g_{i}\left(\bar{x}\left(V\right)\right)\left(V-E_{i}\right)\;,$$ (12)

where $\bar{x}\left(V\right)$ is the steady state of *x*, which can be treated as function of *V* under voltage-clamp conditions. Hence we have

$$\varkappa_{i}=\frac{\bar{I}\left(V\right)}{g_{i}\left(\bar{x}\left(V\right)\right)\left(V-E_{i}\right)}\;,$$ (13)

which can be interpreted as the density that species *i* would in order to be the sole carrier of the observed current, which gives a conservative upper bound (since in reality the current may be born by several species). Since we are looking for an upper bound, we maximise this expression, described by eq (13), with respect to physiologically achievable *V*,

$${\hat{\varkappa }}_{i}=\max_{V}\left\{\frac{\bar{I}\left(V\right)}{g_{i}\left(\bar{x}\left(V\right)\right)\left(V-E_{i}\right)}\right\}\;.$$ (14)

For the calcium-dependent gating variables, we assumed a baseline level of intracellular calcium concentration (100 nM). The lower bounds or minimum possible channel density values are set to zero as no channel density can take a negative value.

**Table 3:** Channel density upper bounds.

Potential conductance species	maximum number of channels/pF
Kv2.1	17
Kv9.3	11.2
Kv6.1	37
BK	1500
BK*β*1	700
BK*β*3	4
BK*β*4	2
SK_2_	366
SK_3_	1590
SK_4_	14
hERG	69.2
Kv4.1	14
Kv4.3	19
Kv4.3+KCNE3	191
Kv4.3+KChIP2b	15
Kv4.3+KChIP2d	10
Kv4.3+KChIP2b+KCNE3	26
Kv3.4	42
Kv7.1	101
Kv7.4	16
Kir7.1	1031
bgK, bgCl	1, 2

#### Calcium spike events (modeling of Hypothesis III)

Two functionally distinct populations of Cl^−^ channel were assumed to exist. The first is affected only by the global calcium concentration, while the second population, due to close spatial proximity of these channels to the sarcoplasmic reticulum, is affected by both global and local fluctuations. This second population corresponds to about 5% of all CaCCs. These local ‘sparks’ in calcium concentration were represented by a forcing function of the following form:

$${\text{Ca}}_{\text{local}}=\sum_{i=1}^{N}\frac{4\;A_{i}\exp\left\{(t-T_{i})/w\right\}}{{\left(1+\exp\left\{(t-T_{i})/w\right\}\right)}^{2}}\;,$$ (15)

where Ca_local_ is the local calcium concentration (in nM), *N* is the number of sparks, and *w* controls the duration of the events (pulse width). The quantities *T*_1_, *T*_2_, … are independent log-normally distributed random variates. Similarly, the quantities *A*_1_, *A*_2_, … that control the amplitude of the sparks, are independent log-normally distributed random variates.

### Mathematical modeling of gating kinetics and estimation of associated biophysical parameters

The complete repertoire of the electrogenic proteins that are potentially expressed in the MSMC was determined on the basis of mRNA expression data, which have been made available as entire raw data via the GEO database (GEO series accession number GSE50599) and, in processed form, as the supplementary material associated with the original publication by Chan et al [13].

We constructed a complete list of every individual oligomeric channel complex that has been attested in the literature and that is consistent with the subunits in the mRNA expression set; the mathematical model, shown in Fig 1A, incorporates 31 time-dependent ionic currents. These currents include outward currents such as the voltage-gated potassium current, the voltage- and calcium-gated potassium current, as well as the calcium-gated potassium current. In addition, the model comprises two inward, depolarising currents attributed to the two voltage-gated calcium channels (L-type and T-type). Chloride fluxes are represented as a calcium-activated chloride current, as well as a background current. A background potassium current was also accounted for. Finally, the model includes pumps and exchangers: the Na^+^-Ca^2+^ exchanger (NCX), the plasma membrane calcium ATPase (PMCA), and the Na^+^-K^+^ pump (NaK). A separate mathematical model for each oligomeric channel was formulated, either based on models that have already been proposed in the literature or on the basis of the available data. The biophysical and kinetic parameters of several of these entities could in many cases be taken directly in the literature, or else have been obtained by means of least-squares fitting to the experimental data on heterologous expression systems, taken from the literature, as detailed below. The mathematical expressions that describe the individual channel kinetics, together with the associated parameters descriptions, units, and values were previously listed in McCloskey et al [39] and are reproduced here for the sake of clarity in the Supplementary Materials, while a summary of the potential conductance species included in our model is shown in Table 1.

#### Delayed rectifier, voltage-gated potassium channel Kv2.1

**Fig 17:**
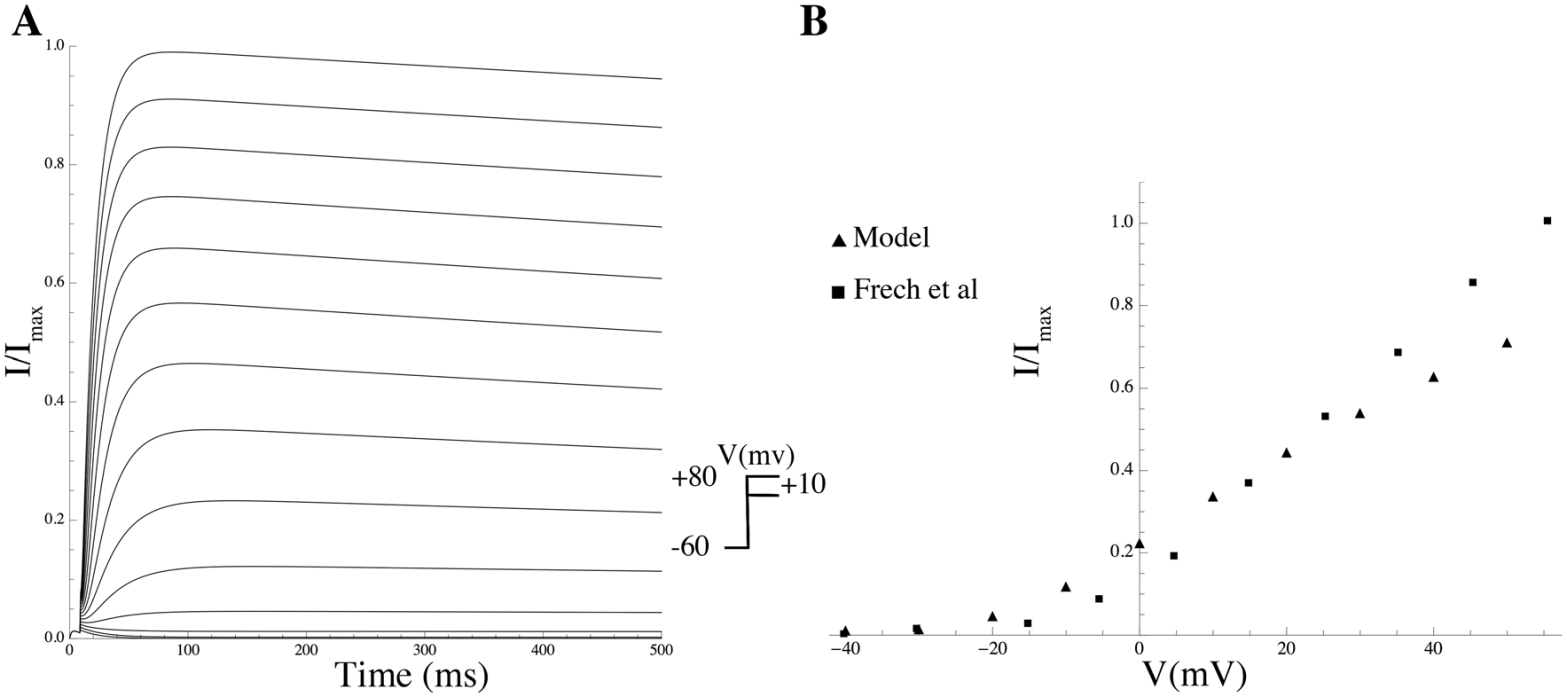
Properties of *I*_Kv2.1_. (A) Normalised *I*_Kv2.1_ current trace in simulated voltage-clamp experiments. Currents are recorded during 1 s voltage steps to potentials ranging from −50 to +80 mV from a holding potential of −60 mV. (B) Data from Frech et al [79] peak *I*–*V* curve (solid squares) obtained from the series of experiments shown in (A); compared to simulation (solid triangles). Values are normalised to the peak current value.

**Fig 18:**
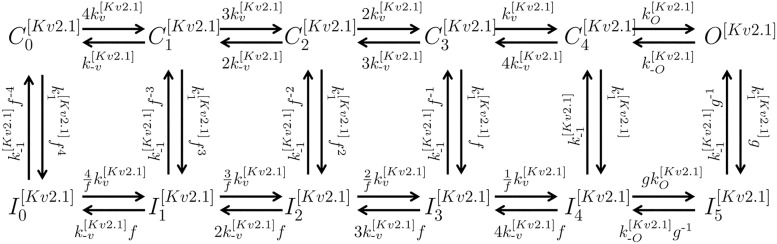
State transition diagram of the Markov model for the Kv2.1 channel. $C_{0}^{\text{[Kv2.1]}}$–$C_{4}^{\text{[Kv2.1]}}$ are the closed states; *O*^[Kv2.1]^ is the open state, and $I_{0}^{\text{[Kv2.1]}}$–$I_{5}^{\text{[Kv2.1]}}$ are the inactivation states; $k_{v}^{\text{[Kv2.1]}}$, $k_{-v}^{\text{[Kv2.1]}}$, $k_{1}^{\text{[Kv2.1]}}$, $k_{-1}^{\text{[Kv2.1]}}$, $k_{0}^{\text{[Kv2.1]}}$, and $k_{-0}^{\text{[Kv2.1]}}$ denote transition rates between the states.

The *I*_Kv2.1_ current is carried by the channel encoded by the *KCNB1* gene. This channel is a voltage-dependent potassium channel related to the shab channel of *Drosophila* [79]. Various lines of evidence indicate that this ion channel plays an important role in uterine contractility: it is highly expressed both in the pregnant and the non-pregnant human uterus at the mRNA level; this was the second-most highly expressed channel after BK [13]. Furthermore, when stromatoxin (ScTx) was applied, the majority of late outward current in MSMCs was inhibited (C. McCloskey, personal communication). Knock et al [80] identified three types of voltage-gated potassium currents in pregnant human myometrium. The first current, IK_1_, was 4-aminopyridine (4-AP)-insensitive with a negative half-inactivation voltage value (*V*_half_ = −61 mV) and slow kinetics. The current was inhibited by tetraethylammonium (TEA; half-maximal block at 3 mM) but insensitive to higher doses. Kv2.1 is the only cloned channel that has similar electrophysiological and pharmacological properties, i.e., slow inactivation, moderate TEA-sensitivity and resistance to block by 4-AP [15]. This suggests that Kv2.1 is a major delayed rectifier channel and plays a key role in returning the depolarised cell back to the resting state. Fig 17 shows the simulated *I*–*V* curve together with the experimental data from expression of the potassium channel gene drk1 (≡ Kv2.1) in *Xenopus* oocytes by Frech et al [79], who found that the channel does not inactivate within 500 ms, in keeping with Klemic et al [14] who observed a slow, weakly voltage-dependent inactivation (*τ* = 4 s at 0 mV; *τ* = 7 s at +80 mV). Kv2.1 forms heteromers with two of the genes (*KCNS3* and *KCNG1*) attested in the mRNA list [13]. These genes form the electrically silent delayed rectifier-like potassium channels Kv9.3 and Kv6.1. When coexisting with Kv2.1, the silencers modify its pharmacological and single-channel properties as well as its kinetics. Separate entities comprising the Kv2.1/Kv6.1 and Kv2.1/Kv9.3 channel complexes were incorporated in the model. The biophysical characteristics observed in heterologous expression systems closely match those of delayed rectifier currents [81]. It activates as a result of a brief depolarisation, exhibiting a sigmoidal time course [81] with *V*_half_ = 20 mV and slope *k* = 19.6 mV [15], then it slowly inactivates during sustained depolarisation with *V*_half_ = −29.8 mV, and *k* = −11.1 mV [15]. This slow inactivation is possibly physiologically irrelevant for a brief AP, but a train of depolarizations may result in a cumulative inactivation. To account for this effect, we use a Markov model which incorporates the slow inactivation-related gating states [82]. We adopted the model proposed by Klemic et al [14], shown in Fig 18. The resulting voltage-dependent inactivation becomes apparent during repetitive spiking; the dependence is ∪-shaped, with the inactivation being lowest at strong depolarizations. Current (expressed in pA/pF) through this channel is described by the following equation:

$$I_{\text{Kv2.1}}=\kappa_{\text{Kv2.1}}G_{\text{Kv2.1}}O^{\text{[Kv2.1]}}\left(V-E_{\text{K}}\right)\;,$$ (16)

where *G*_Kv2.1_ is the single-channel conductance (pS) and *O*^[Kv2.1]^ is the probability of the Kv2.1 channel being in the open state.

The parameters of this model satisfy the principle of detailed balance (microscopic reversibility). For instance, along the left-most loop of the diagram in Fig 18, this principle requires that

$$1=\frac{4k_{v}^{\left[Kv2.1\right]}f^{3}k_{1}^{\left[Kv2.1\right]}fk_{-v}^{\left[Kv2.1\right]}f^{-4}k_{-1}^{\left[Kv2.1\right]}}{k_{-v}^{\left[Kv2.1\right]}f^{-3}k_{1}^{\left[Kv2.1\right]}4f^{-1}k_{v}^{\left[Kv2.1\right]}f^{4}k_{-1}^{\left[Kv2.1\right]}}$$ (17)

which is identically true.

#### Delayed rectifier, voltage-gated, heteromeric potassium channel Kv2.1/Kv9.3

**Fig 19:**
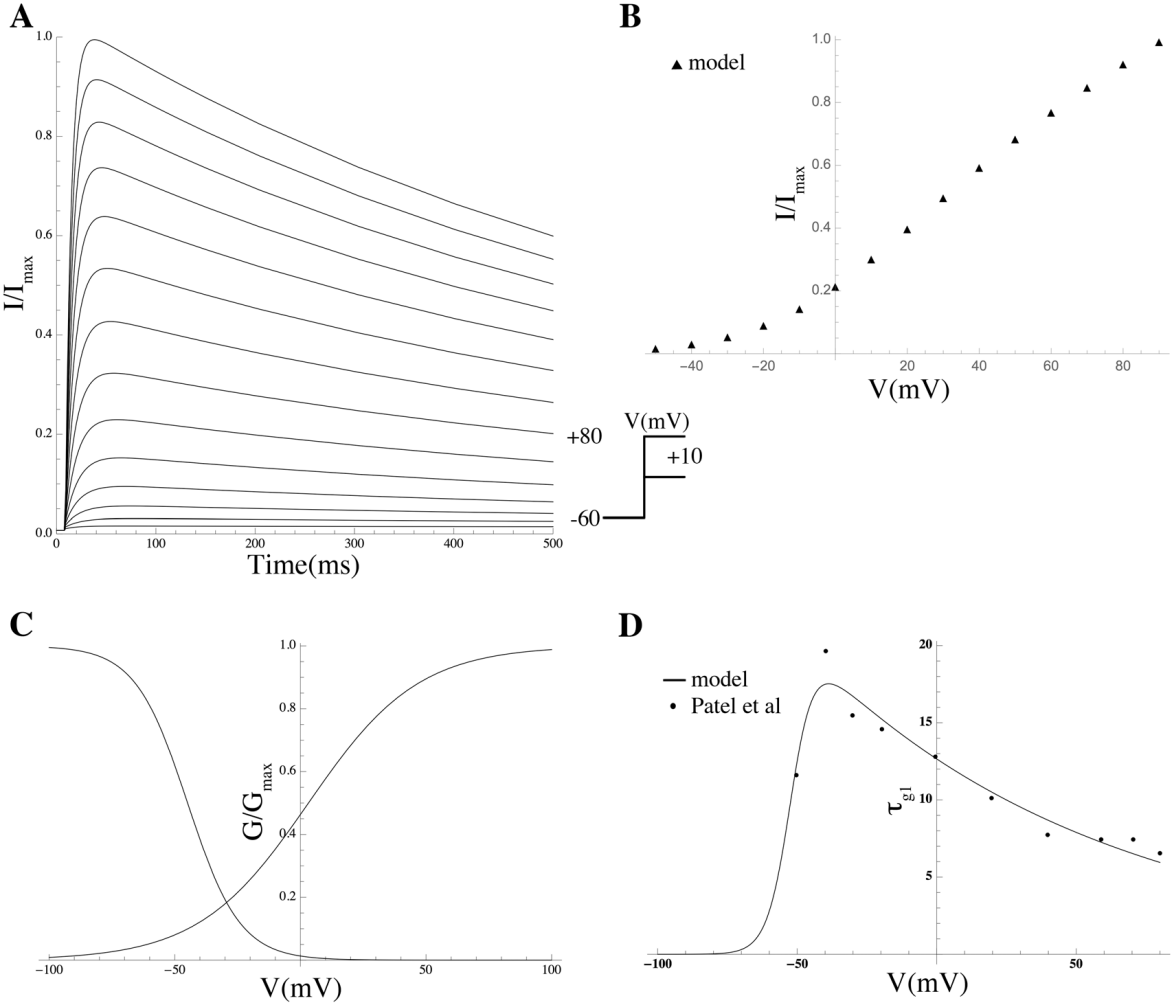
Properties of *I*_Kv9.3_. (A) Normalised *I*_Kv9.3_ current trace in simulated voltage-clamp experiments. Currents are recorded during 1 s voltage steps to potentials ranging from −50 to 80 mV from a holding potential of −60 mV. (B) Simulated (solid triangles) peak *I*–*V* relationship obtained from the series of experiments shown in (A). Values are normalised to the peak current value. (C) Steady state activation and inactivation curves from Patel et al [15]. (D) Simulated activation time constant derived from experimental data (filled circles) from Patel et al [15].

The current I_Kv9.3_ is carried by the heteromeric Kv2.1/Kv9.3 channel, which arises through co-expression of the Kv2.1 channel with the electrically silent delayed rectifier-like potassium channel Kv9.3. The gene encoding the Kv9.3 channel, *KCNS3*, was attested in the mRNA expression data [13]. When Kv9.3 was expressed together with Kv2.1 in *Xenopus* oocytes by Patel et al [15], several alterations were observed in the pharmacological properties, single-channel properties, and kinetics of Kv2.1: the single-channel conductance increased, the activation threshold was displaced towards more negative values and the amplitude of the current was enhanced. Activation and inactivation curves of Kv2.1 were both shifted towards more negative values by about 20 mV, which leads to faster activation and inactivation [15]. Kv2.1 inactivates slowly from both the open and intermediate closed states whereas Kv2.1/Kv9.3 does not inactivate from the open state but rapidly does so from the intermediate closed state. The result is a ∪-shaped steady-state inactivation-voltage curve [83]. We adopted the Boltzmann function proposed by Patel et al [15]. For activation, *V*_half_ = 3.2 mV and *k* = 21.8 mV, while for inactivation *V*_half_ = −44.9 mV and *k* = −10.4 mV [15]. The time constant *τ*_*g*1_ for activation was derived by least-squares fitting to kinetic data taken from Patel et al [15]. Inactivation of Kv2.1/Kv9.3 was faster than Kv2.1 but still relatively slow. The inactivating current was fitted to experimental data taken from Kerschensteiner et al [83] using a double-exponential model with time constants *τ*_*g*_2_fast___ = 0.63 s and *τ*_*g*_2_slow___ = 3.1 s; Fig 19 shows the gating kinetics of the channel. The current carried by Kv2.1/Kv9.3 is described by the following equation:

$$I_{\text{Kv9.3}}=\kappa_{\text{Kv9.3}}\;G_{\text{Kv9.3}}\;g_{1}\;\left(0.7g_{2_{\text{fast}}}+0.3g_{2_{\text{slow}}}\right)\;\left(V-E_{\text{K}}\right)\;,$$ (18)

where *G*_Kv9.3_ is the unitary conductance of the Kv2.1/Kv9.3 channel, and *g*_1_ and *g*_2_ represent the activation and inactivation gating variables, respectively.

#### Delayed rectifier, voltage-gated, heteromeric potassium channel Kv2.1/Kv6.1

**Fig 20:**
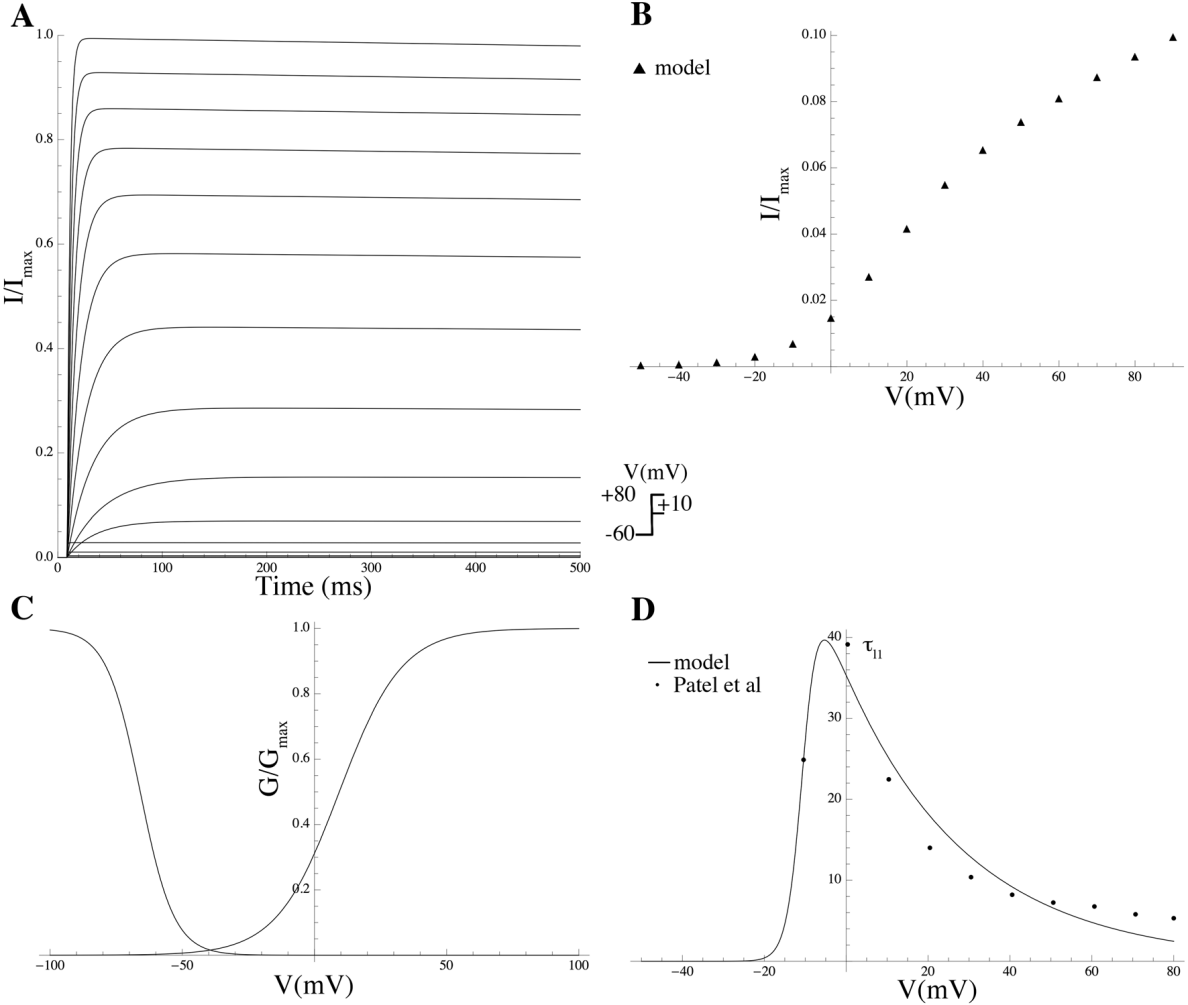
Properties of *I*_Kv6.1_. (A) Normalised *I*_Kv6.1_ current trace in simulated voltage-clamp experiments. Currents are recorded during 1 s voltage steps to potentials ranging from −50 to 80 mV from a holding potential of −60 mV. (B) Simulated (solid triangles) peak *I*–*V* relationship obtained from the series of experiments shown in (A). Values are normalised to the peak current value. (C) Steady state activation and inactivation curves from Kramer et al [16]. (D) Simulated activation time constant derived from experimental data (filled circles) from Patel et al [15].

The channel Kv2.1 can form another heteromeric complex with the electrically silent delayed rectifier Kv6.1, conducting the current *I*_Kv6.1_ [84]. The gene encoding the Kv6.1 channel, *KCNG1*, was present at the mRNA expression level in MSMCs [13]. Kramer et al [16] co-expressed Kv2.1 with Kv6.1 in *Xenopus* oocytes and found that Kv6.1 failed to accelerate the inactivation of Kv2.1 at intermediate potentials, had a slowing effect on inactivation at strong depolarisations, but had no effect on cumulative inactivation. Furthermore, Kv6.1 strongly affected activation of Kv2.1, shifting the steady-state activation curve towards more negative potentials [16]. The single-channel conductance is not known; we assume it to be similar to the heteromeric channel Kv2.1/Kv5.1, which is about 12.5 pS [16]. The parameter *V*_half_ for activation was −9.4 mV and the slope was 11.8 mV, while for inactivation, *V*_half_ = −65.9 mV and *k* = −6.4 mV. Because the presence of Kv6.1 had no significant effect on the activation time constant [16], we assumed the parameter *τ*_*l*_1__ for activation to be the same as the Kv2.1 activation time constant given by Patel et al [15]. Fig 20 shows the gating kinetics of the channel; it can be seen that Kv2.1/Kv6.1 exhibits extremely slow exponential decay current with a voltage-independent *τ*_*l*_2__ = 32 s, which suggests that the regulatory subunit Kv6.1 virtually abrogates inactivation. The current is described by the following equation:

$$I_{\text{Kv6.1}}=\kappa_{\text{Kv6.1}}G_{\text{Kv6.1}}l_{1}\;l_{2}\left(V-E_{\text{K}}\right)\;,$$ (19)

where *l*_1_ and *l*_2_ represent the activation and inactivation gating variables, respectively.

#### A-type, voltage-gated potassium channel Kv3.4

**Fig 21:**
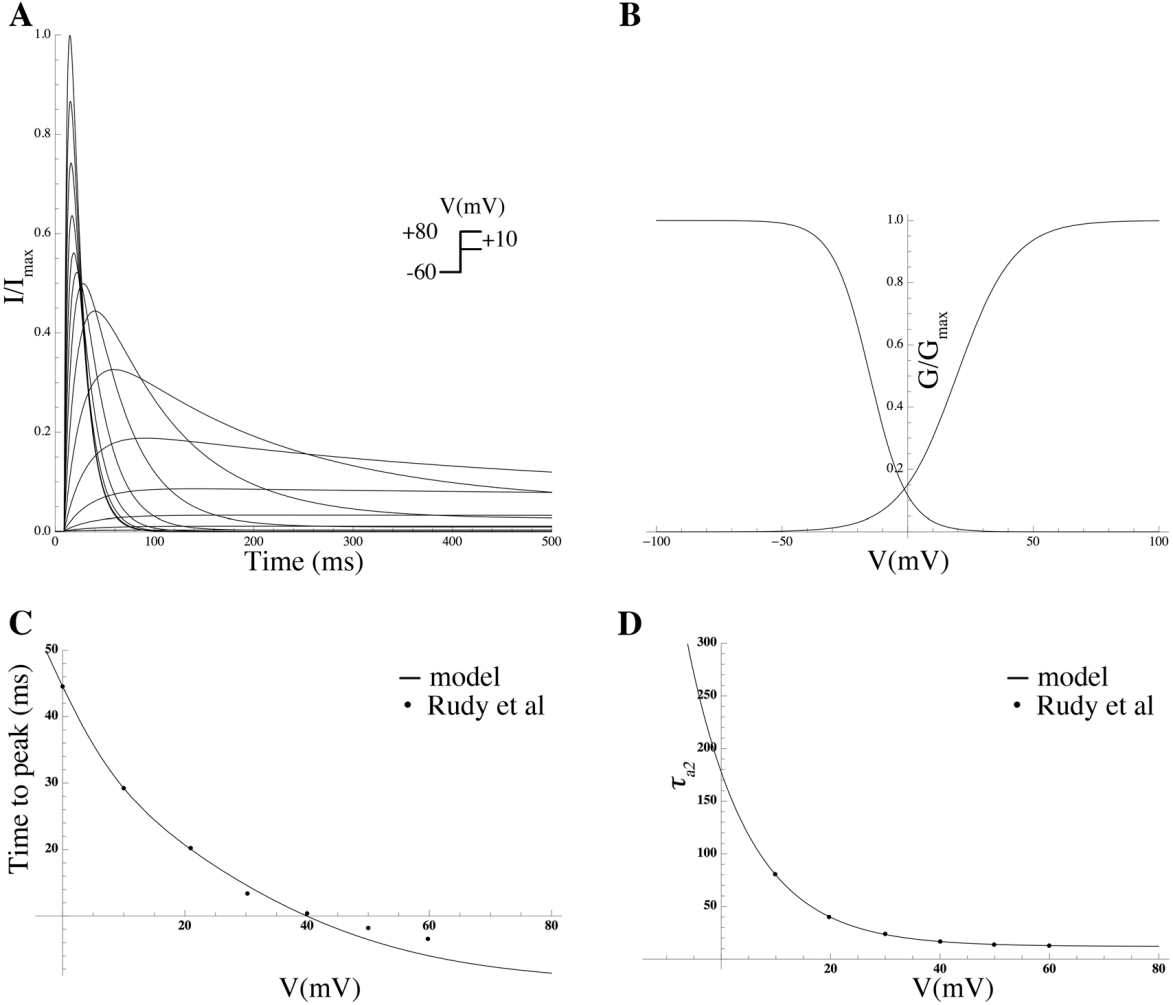
Properties of *I*_Kv3.4_. (A) Normalised *I*_Kv3.4_ current trace in simulated voltage-clamp experiments. Currents are recorded during 1 s voltage steps to potentials ranging from −50 to 80 mV from a holding potential of −60 mV. (B) Steady state activation and inactivation curves from Rudy et al [28] (C) Simulated time-to-peak derived from data (filled circles) from Rudy et al [28]. (D) Simulated inactivation time constant derived from experimental data (filled circles) from Rudy et al [28].

The current *I*_Kv3.4_ is carried by the channel encoded by the *KCNC4* gene. This is an A-type fast inactivating potassium channel, responsible for a transient outward current and operating in the subthreshold range of the AP. Current starts activating at high voltages, around −10 mV, rising fast before quickly inactivating, this rapid inactivation being due to the specialised N-terminus that closes the channel from the intracellular side. Rudy et al [28] demonstrated an A-current in *Xenopus* oocytes when co-expressed with human potassium shaker channel HKShIIIC (≡ Kv3.4). For activation, *V*_half_ is 19.1 mV and the slope *k* is 11.3 mV, while for inactivation *V*_half_ = −15 mV and *k* = −7.4 mV [28]. The activation and inactivation gating variables are *a*_1_ and *a*_2_, respectively, and the corresponding steady-state activation and inactivation variables are *a*_1∞_ and *a*_2∞_. An empirical function was chosen for the activation time constants *τ*_*a*_1__ to fit the equation *t*_*p*_ = *τ*_*a*_1__ ln{1 + *nτ*_*a*_2__/*τ*_*a*_1__} [85] to time-to-peak experimental data taken from Rudy et al [28], where *n* is the order of the activation kinetics (here *n* = 1). For inactivation, *τ*_*a*_2__ was optimised to fit experimental data from Rudy et al [28]. Fig 21 shows the gating kinetics of the channel. The current is given by the following expression:

$$I_{\text{Kv3.4}}=\kappa_{\text{Kv3.4}}\;G_{\text{Kv3.4}}\;a_{1}\;a_{2}\;\left(V-E_{\text{K}}\right)\;.$$ (20)

#### A-type, voltage-gated potassium channel Kv4.1

**Fig 22:**
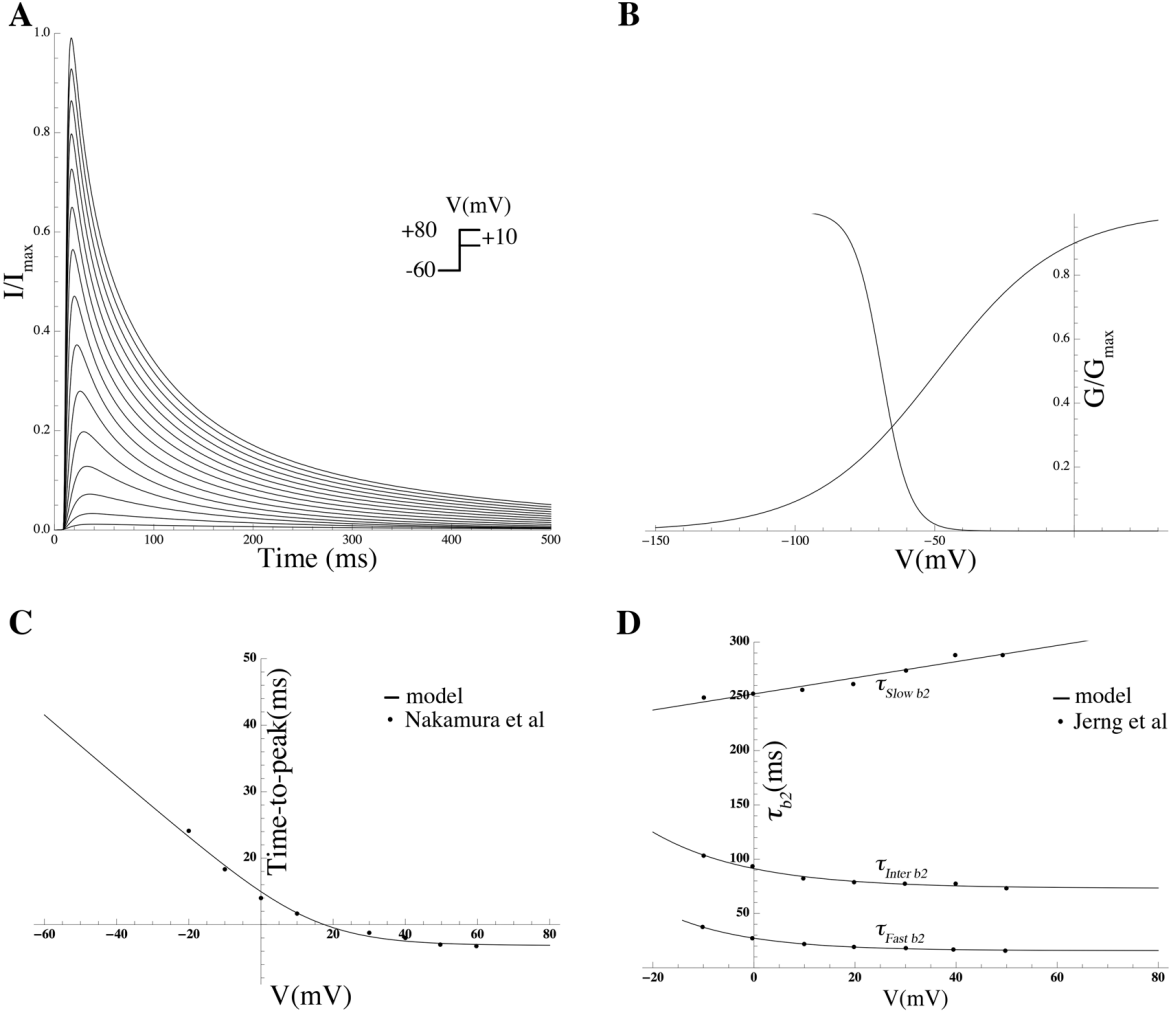
Properties of *I*_Kv4.1_. (A) Normalised *I*_Kv4.1_ current trace in simulated voltage-clamp experiments. Currents are recorded during 1 s voltage steps to potentials ranging from −50 to 80 mV from a holding potential of −60 mV. (B) Steady state activation and inactivation curves from Jerng et al [29]. (C) Simulated time-to-peak derived from data (filled circles) from Nakamura et al [30]. (D) Simulated inactivation time constants derived from experimental data (filled circles) from Jerng et al [29].

Another transient A-type potassium current is *I*_Kv4.1_, carried by the Kv4.1 channel, which according to the mRNA data is expressed in MSMSs; the gene encoding the channel is the *KCND1* gene [13]. Jerng et al [29] modeled activation as fourth-order Boltzmann function. The activation time constant *τ*_*b*_1__ was derived from the time-to-peak data provided by Nakamura et al [30] in a similar manner to the derivation of *τ*_*a*_1__ (for the Kv3.4 channel) with *n* = 4. Inactivation follows a more complex time course which was described as the sum of three exponential terms. The corresponding time constants *τ*_fast*b*_2__, *τ*_inter*b*_2__, and *τ*_slow*b*_2__, representing fast, intermediate and slow kinetics, respectively, were obtained by fitting the sum of the three terms to the decay phase of the current. The corresponding relative magnitudes were assumed to be time- and voltage-independent and equal to 18%, 42%, and 40%, respectively. The intermediate and slow components account for the recovery from inactivation, which determines the inter-spike interval during repetitive firing. The inactivation exponential functions were fitted to data from Jerng et al [29]. Fig 22 summarises the gating kinetics. The current is described by the following expression:

$$I_{\text{Kv4.1}}=\kappa_{\text{Kv4.1}}\;G_{\text{Kv4.1}}\;{b_{1}}^{4}\;\left(0.18\;b_{2,\text{fast}}+0.42\;b_{2,\text{inter}}+0.4\;b_{2,\text{slow}}\right)\;\left(V-E_{\text{K}}\right)\;,$$ (21)

where *b*_1_ and *b*_2_ represent the activation and inactivation gating kinetics, respectively, with corresponding steady-state activation and inactivation variables *b*_1∞_ and *b*_2∞_. For activation, we used *V*_half_ = − 49 mV and *k* = 22.3 mV, whereas for inactivation we used *V*_half_ = − 69 mV and *k* = − 5 mV.

#### A-type, voltage-gated potassium channel Kv4.3

**Fig 23:**
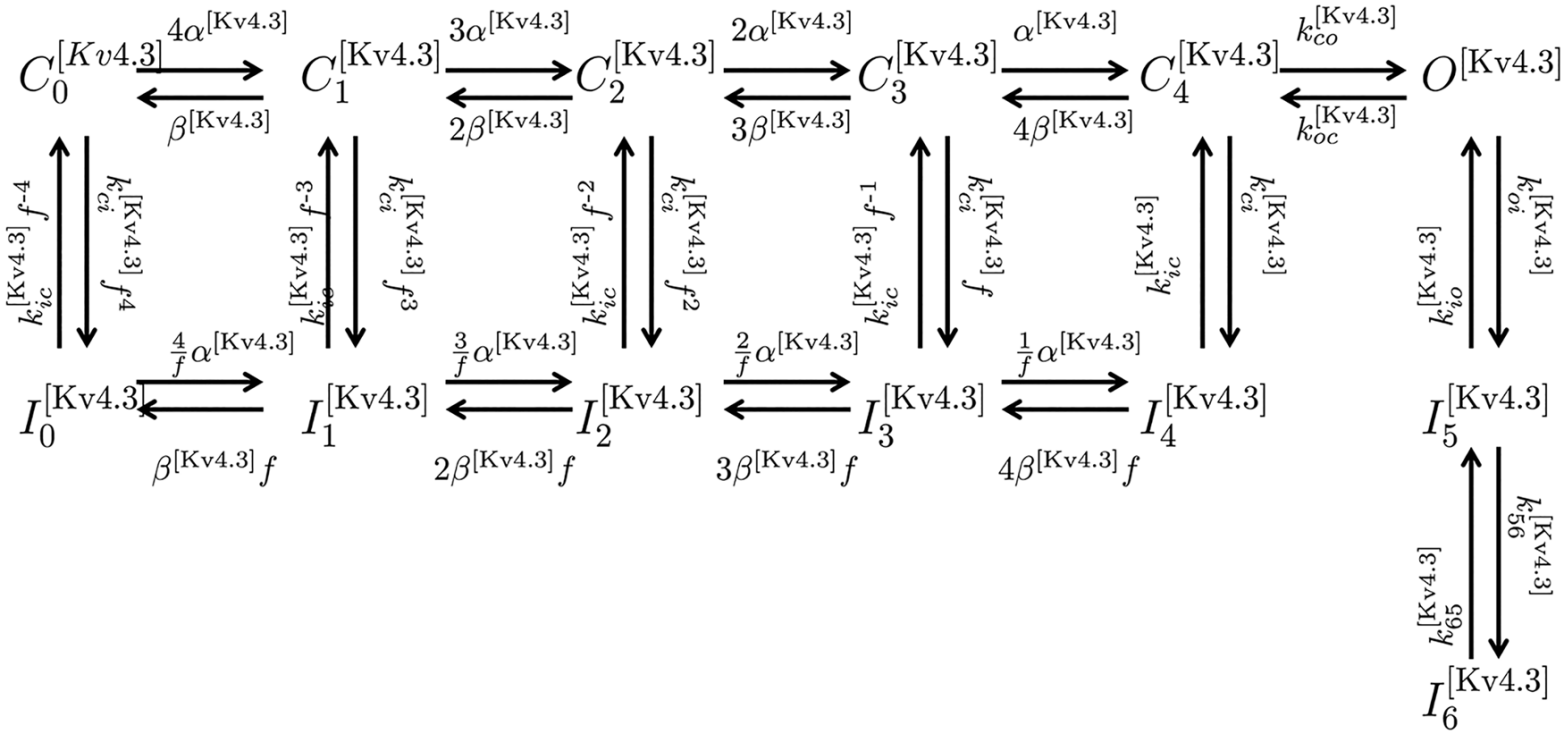
State transition diagram of the Markov model for the Kv4.3 channel. $C_{0}^{\text{[Kv4.3]}}$–$C_{4}^{\text{[Kv4.3]}}$ are the closed states; *O*^[Kv4.3]^ is the open state and $I_{0}^{\text{[Kv4.3]}}$–$I_{6}^{\text{[Kv4.3]}}$ are the inactivation states; *α*^[Kv4.3]^, *β*^[Kv4.3]^, $k_{ci}^{\text{[Kv4.3]}}$, $k_{ic}^{\text{[Kv4.3]}}$, $k_{oc}^{\text{[Kv4.3]}}$, $k_{co}^{\text{[Kv4.3]}}$, $k_{56}^{\text{[Kv4.3]}}$ and $k_{65}^{\text{[Kv4.3]}}$ denote transition rates between the states.

**Fig 24:**
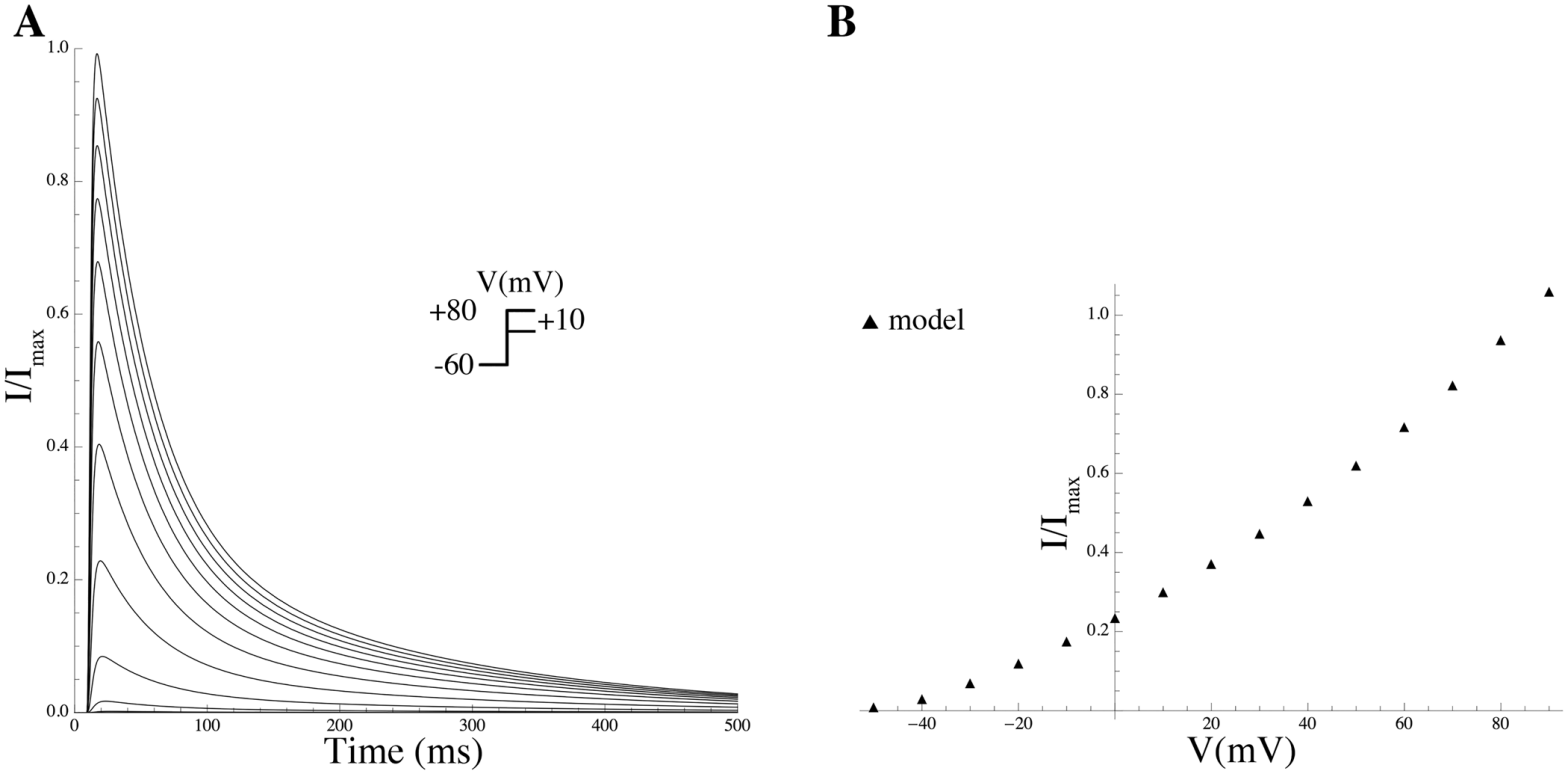
Properties of *I*_Kv4.3_. (A) Normalised *I*_Kv4.3_ current trace in simulated voltage-clamp experiments. Currents are recorded during 1 s voltage steps to potentials ranging from −50 to 80 mV from a holding potential of −60 mV. (B) Simulated (solid triangles) peak *I*–*V* relationship obtained from the series of experiments shown in (A). Values are normalised to the peak current value.

A voltage-gated potassium current is carried by the Kv4.3 channel encoded by the *KCND3* gene, which is prominent at the transcriptional level [13]. Key characteristics include a transient outward current as well as fast activation and inactivation; Patel et al [32] give *V*_half_ = −7.9 mV, *k* = 12.34 mV for activation, and *V*_half_ = −68.9 mV, *k* = −6.31 mV for inactivation. Inactivation of the Kv4.3 is a complex process that can occur from both closed and open states [32, 86]. Beck et al [31] expressed Kv4.3 in *Xenopus* oocytes and modeled the changes in its kinetics using the allosteric model shown in Fig 23. Fig 24 summarises the gating kinetics. The current is given by the following expression:

$$I_{\text{Kv4.3}}=\kappa_{\text{Kv4.3}}\;G_{\text{Kv4.3}}\;O^{\text{[Kv4.3]}}\;\left(V-E_{\text{K}}\right)\;,$$ (22)

where *O*^[Kv4.3]^ denotes the open state probability of the Kv4.3 channel (see Fig 23). The principle of microscopic reversibility is obeyed by the parameters of the Kv4.3 Markov chain model.

#### Voltage-gated Kv4.3/KChIP2

A range of auxiliary subunits is involved in the modulation of Kv4 channels (Kv4.2 and Kv4.3 in particular); one family of regulatory subunits that appears to be of physiological significance is comprised by the KChIPs [31]. Members of this family, when heterologously co-expressed with Kv4 *α*-subunits, were shown to increase cell surface expression, slow down inactivation kinetics, and accelerate recovery kinetics [32]. A regulatory characteristic of KChIPs on Kv4 channels is to increase cell surface expression as presented by an increase in peak macroscopic current amplitude, with no effect on unitary channel conductance [31]. Patel et al [32] analysed the voltage-dependence of inactivation, recovery from activation, and deactivation kinetics of Kv4.3 expressed in the presence of KChIP2b and KChIP2d. Activation was modeled as a fourth-order Boltzmann function. Inactivation follows a complex time course, well-described by the sum of two exponential terms representing the fast and slow kinetics, respectively. The corresponding time constants and relative magnitudes are voltage-dependent. The current for both channels takes the following general form:

$$I_{□}=\kappa_{□}\;G_{□}\;{k_{1,□}}^{4}\;\left(A_{\text{fast},□}\;k_{2_{\text{fast}},□}+A_{\text{slow},□}\;k_{2_{\text{slow}},□}\right)\;\left(V-E_{\text{K}}\right)\;,$$ (23)

where the wildcard ‘□’ stands for either Kv4.3/KChIP2b or Kv4.3/KChIP2d, *k*_1_ and *k*_2_ represent the activation and inactivation gates with corresponding steady-state activation and inactivation parameters *k*_1∞_ and *k*_2∞_. For the steady state activation of Kv4.3/KChIP2b, we used *V*_half_ = −2.97 mV and *k* = 12.64 mV, whereas for inactivation, *V*_half_ = − 57.4 mV and *k* = − 4.78 mV. For activation of Kv4.3/KChIP2d, we used the values *V*_half_ = − 2.3 mV and *k* = 12.49 mV, and for inactivation, we used *V*_half_ = − 61.1 mV and *k* = − 5 mV.

#### Voltage-gated Kv4.3/KCNE3 and Kv4.3/KCNE3/KChIP2

The KCNE3 *β*-subunit has a strong inhibitory effect on heterologously expressed Kv4.3 channels; the current amplitude is reduced and, moreover, activation, inactivation and recovery from inactivation are slowed down [87]. KCNE3 also inhibits currents generated by Kv4.3 in complex with the accessory subunit KChIP2. We modeled two separate entities: one corresponding to Kv4.3 co-expressed with KCNE3 and another for Kv4.3 co-expressed with both KCNE3 and KChIP2. We extracted the data from Lundby et al [87] who analysed the voltage-dependence of activation, inactivation, and recovery from inactivation for Kv4.3 co-expressed with KCNE3 in the presence and absence of KChIP2. For Kv4.3/KCNE3, the steady state activation, we used the values *V*_half_ = 6 mV and *k* = 17.5 mV, whereas for inactivation *V*_half_ = − 72 mV and *k* = − 11.1 mV were used. For Kv4.3/KCNE3/KChIP2 activation, *V*_half_ = − 5 mV and *k* = 17.5 mV, and for inactivation we set *V*_half_ = − 56 mV and *k* = − 11.1 mV. The corresponding time constants were assumed to be voltage-independent. The current for both channels takes the following general form:

$$I_{□}=\kappa_{□}\;G_{□}\;m_{1,□}\;m_{2,□}\;m_{3,□}\;\left(V-E_{\text{K}}\right)\;,$$ (24)

where the wildcard ‘□’ stands for either Kv4.3/KCNE3 or Kv4.3/KCNE3/KChIP2d, *m*_1_ and *m*_2_ represent the activation and inactivation gates with corresponding steady-state activation and inactivation parameters *m*_1∞_ and *m*_2∞_. We postulated an additional gating variable *m*_3_ to account for the recovery from inactivation with steady-state variable *m*_2∞_.

#### Voltage-gated potassium channel hERG

**Fig 25:**
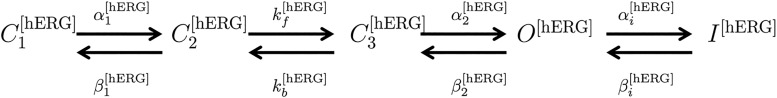
State transition diagram of the Markov model for the hERG channel. $C_{1}^{\text{[hERG]}}$–$C_{3}^{\text{[hERG]}}$ are closed states, *O*^[hERG]^ is the open state and *I*^[hERG]^ the inactivation state.

**Fig 26:**
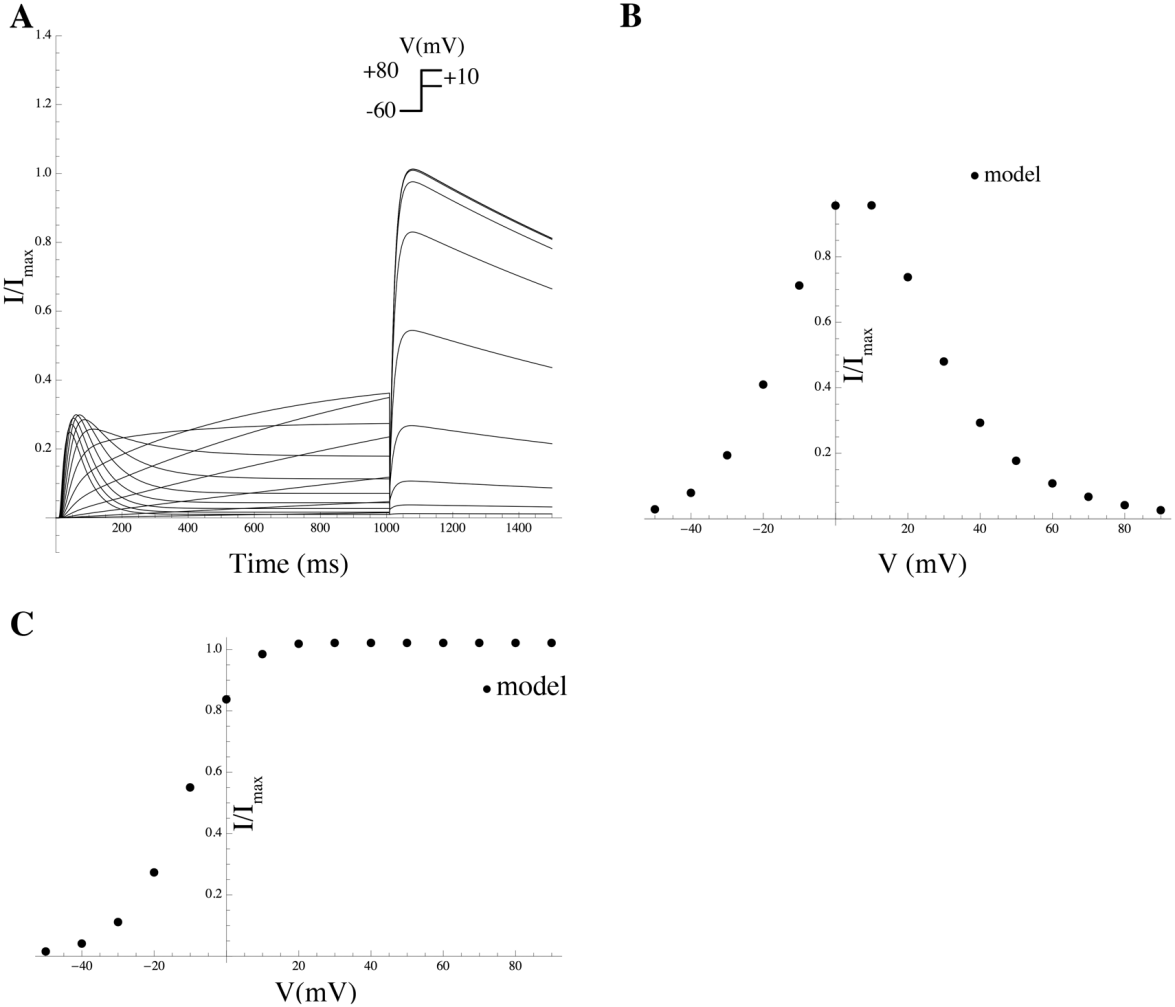
Properties of *I*_hERG_. (A) Simulated *I*_hERG_ current trace in simulated voltage-clamp experiments. Currents are recorded during 1 s voltage steps to potentials ranging from −50 to 80 mV from a holding potential of −60 mV. Values are normalised to the peak current value. (B) Simulated *I*–*V* relationship obtained from the series of experiments shown in (A) at *t* = 400 ms. Values are normalised to the peak current value at the same time point. (C) Simulated peak tail *I*–*V* relationship obtained from the series of experiments shown in (A). Values are normalised to the peak tail current value.

**Fig 27:**
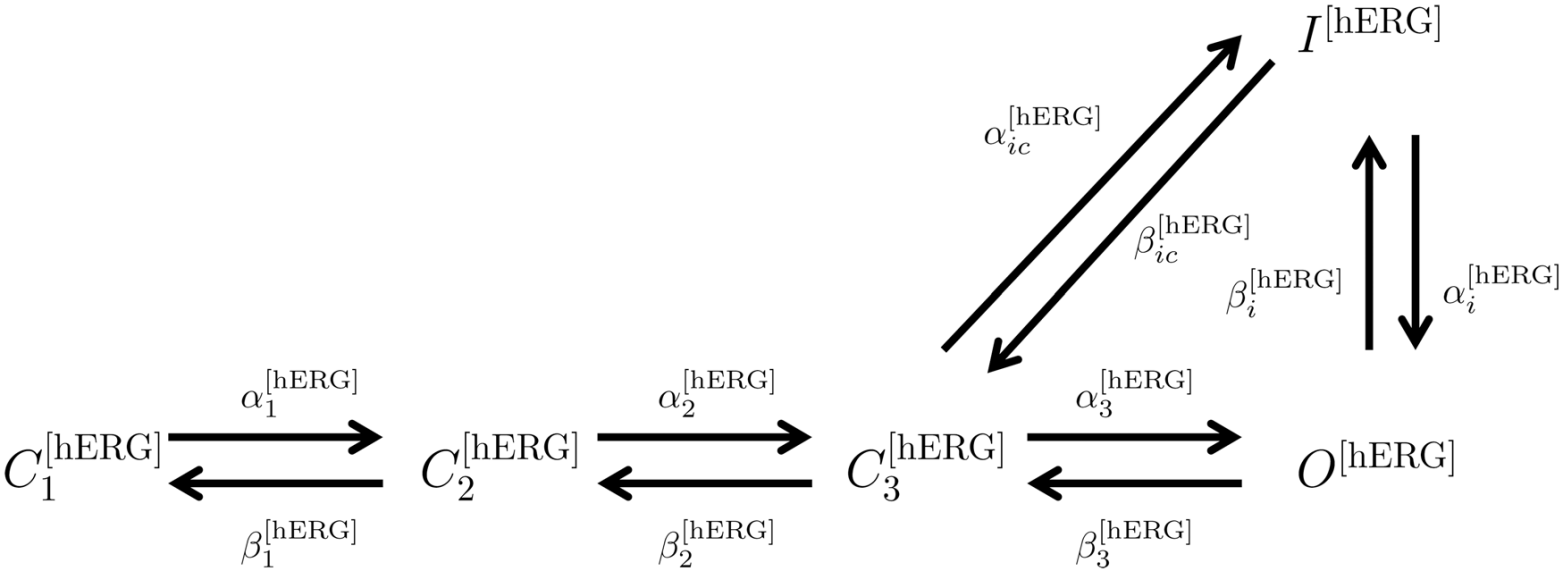
State transition diagram of the Markov model for the effect of PIP_2_ on the hERG channel. $C_{1}^{\text{[hERG]}}$–$\;C_{3}^{\text{[hERG]}}$ are closed states, *O*^[hERG]^ is the open state, and *I*^[hERG]^ the inactivation state. The transition rates have different values depending on whether PIP_2_ is absent or present [55].

The hERG (the human *ether-à-go-go*-related gene), alias *KCNH2* gene, encodes a protein known as Kv11.1 potassium ion channel, which is voltage-gated with inward rectification. According to Sanguinetti et al [88], the magnitude of its current increases up to −10 mV, then progressively decreases with potentials ≥ 0 mV, resulting in a negative slope of the *I*–*V* relationship. The peak outward current decreases with incremental depolarisation, which implies rectification, possibly due to inactivation being much more rapid than activation [88]. Activation of the current competes with rapid inactivation, resulting in a reduction of the current magnitude, as compared to what may be expected on the basis of steady-state activation and the driving force for the outward current; moreover, recovery from inactivation is much faster than deactivation [89]. We modeled the channel following Wang et al [25], who expressed hERG in *Xenopus* oocytes and observed a sigmoidal time course for current activation; these authors postulated a Markov model with three closed states (Fig 25), the intermediate closed state being voltage-insensitive. The properties of hERG resulting from the Markov model used are shown in Fig 26. The current is given by the following expression:

$$I_{\text{hERG}}=\kappa_{\text{hERG}}\;G_{\text{hERG}}\;O^{\left[\text{hERG}\right]}\;\left(V-E_{\text{K}}\right)\;,$$ (25)

where *O*^[hERG]^ denotes the open-channel probability. An variant version, based on the work of Rodriguez et al [55], was used to simulate the effects of PIP_2_; this model is shown in Fig 27. The principle of detailed balance (microscopic reversibility) requires

$$\frac{\alpha_{iC}^{\text{[hERG]}}}{\beta_{iC}^{\text{[hERG]}}}=\frac{\alpha_{i}^{\text{[hERG]}}}{\beta_{i}^{\text{[hERG]}}}\cdot \frac{\alpha_{3}^{\text{[hERG]}}}{\beta_{3}^{\text{[hERG]}}}\;.$$ (26)

The parameter values provided by Rodriguez et al [55] are in agreement with this constraint.

#### Delayed rectifier (voltage-gated) potassium channel Kv7.1

**Fig 28:**
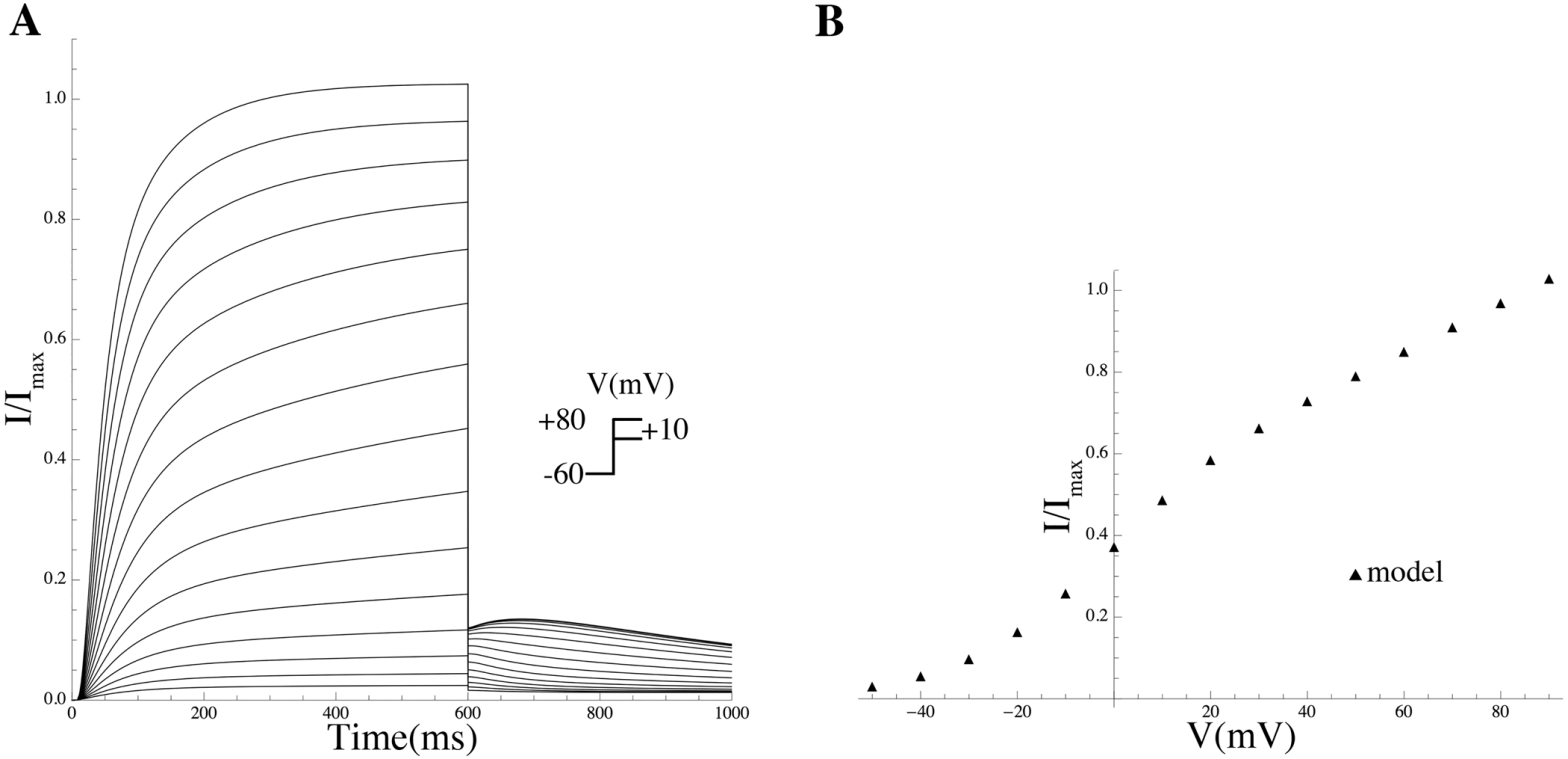
Properties of *I*_Kv7.1_. (A) Simulated voltage clamp traces of homomeric Kv7.1 channel from holding potential of −60 mV, where the voltage was stepped to values up to +80 mV in 10 mV increments. (B) Simulated peak *I*–*V* relationship obtained from the voltage clamp experiments shown in (A). Values are normalised to the peak current values.

**Fig 29:**
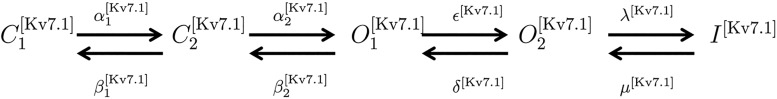
State transition diagram of the Markov model for the Kv7.1 channel. $C_{1}^{\text{[Kv7.1]}}$–$C_{2}^{\text{[Kv7.1]}}$ are the closed states, $O_{1}^{\text{[Kv7.1]}}$–$O_{2}^{\text{[Kv7.1]}}$ are the open states and *I*^[Kv7.1]^ the inactivation state. $\alpha_{1}^{\text{[Kv7.1]}}$, $\beta_{1}^{\text{[Kv7.1]}}$, $\alpha_{2}^{\text{[Kv7.1]}}$, $\beta_{2}^{\text{[Kv7.1]}}$, *λ*^[Kv7.1]^, *μ*^[Kv7.1]^, *δ*^[Kv7.1]^, and *ϵ*^[Kv7.1]^ denote transition rates between the states.

The channel Kv7.1 mediates a delayed rectifier potassium current and is encoded by the *KCNQ1* gene; when associated with the small subunit *KCNE1*, this channel behaves as a slow delayed rectifier voltage-gated potassium channel [90, 91]. Activators of Kv7 can act as myometrium relaxants in pregnant mice and humans [92]. There was no conclusive evidence for expression of the KCNE1 gene in the mRNA expression data [13]. Unlike Kv7.1/ *KCNE1*, a fast outward current is mediated by homomeric Kv7.1 [90, 91]. The latter has been analysed in a *Xenopus* oocyte expression system [33]. A delayed inactivation process that follows the channel activation is indicated by a transient increase in conductance after membrane repolarisation to negative values (manifested by an increase in the tail current), as shown in Fig 28; the inactivation of Kv7.1 is incomplete even at positive voltages [33]. An allosteric gating model can account for the delayed, voltage-dependent onset and for the incompleteness of inactivation [33]. The model contains two open states and a voltage-independent inactivating step. The current through the homomeric Kv7.1 channel is as follows:

$$I_{\text{Kv7.1}}=\kappa_{\text{Kv7.1}}\;G_{\text{Kv7.1}}\;P_{o}^{\text{[Kv7.1]}}\;\left(V-E_{\text{K}}\right)\;,$$ (27)

where $P_{o}^{\text{[Kv7.1]}}$ is the probability of the Kv7.1 channel being in the open state (cf. Fig 29). Members of the *KCNE* family of accessory *β*-subunits modulate Kv7.1 in distinct ways; when co-expressed with *KCNE3* (≡ MiRP2), which has been attested at the mRNA expression level [13], Kv7.1 loses its voltage-dependence gating properties and produces an instantaneous, virtually ohmic whole-cell current [93]. Accordingly, we modeled the heteromer Kv7.1/*KCNE3* as a potassium leak channel (see below). In sharp contrast, the *KCNE4* *β*-subunit, which is found to be highly abundant at the mRNA expression level [13], has a dramatic inhibitory effect on Kv7.1 in heterologous systems [94]. Moreover, *KCNE4* can inhibit the Kv7.1 current, even in the presence of other accessory subunits [95].

#### Delayed rectifier (voltage-gated) potassium channel Kv7.4

**Fig 30:**
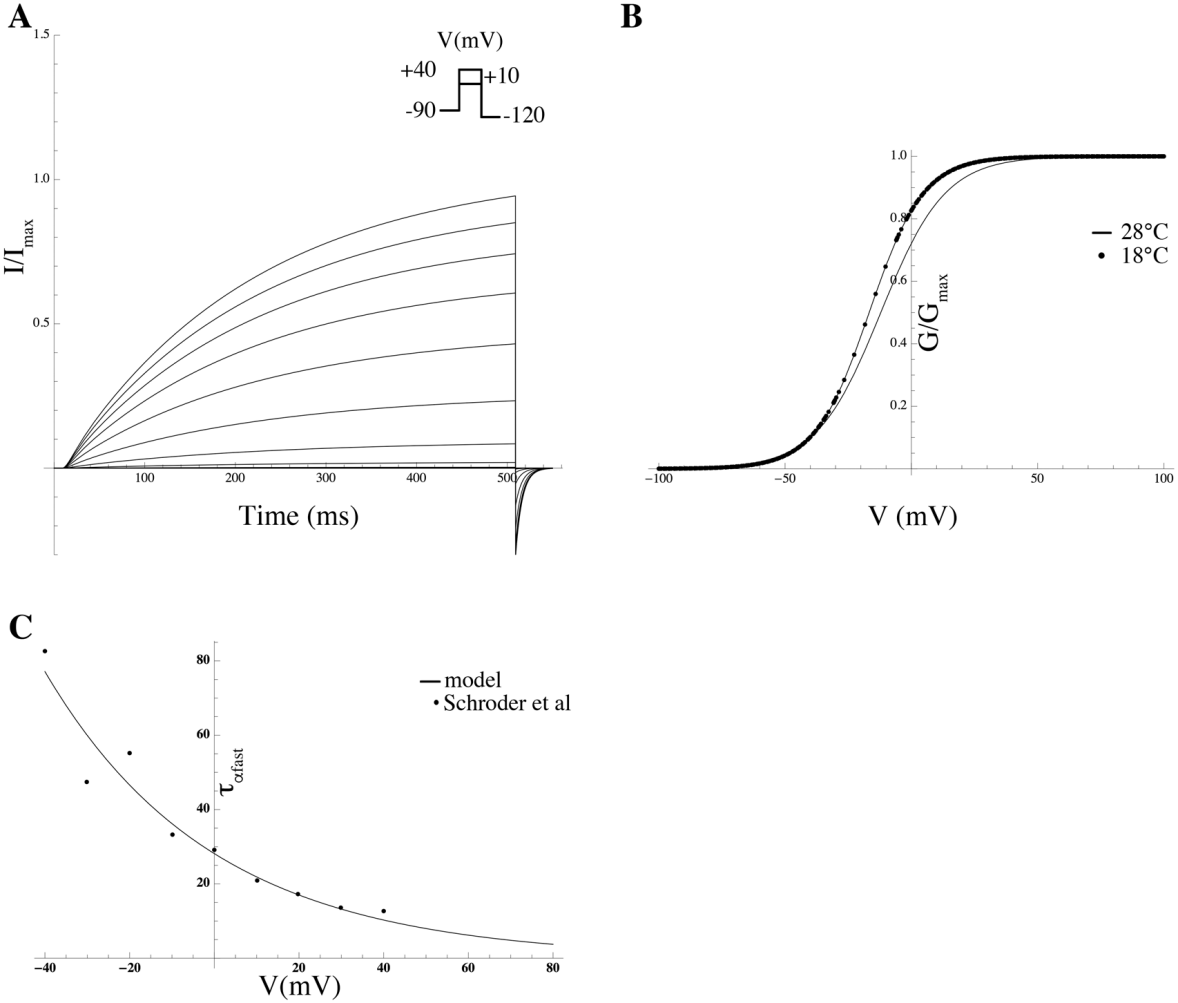
Properties of *I*_Kv7.4_. (A) Simulated voltage clamp traces of homomeric Kv7.4 channel at 28°C from holding potential of −90 mV, where the voltage was stepped to values up to +40 mV in increments of 10 mV, subsequently stepped down to −120 mV. (B) Steady state activation curves from Miceli et al [96]. (C) Simulated activation fast time constant derived from experimental data (filled circles) from Schröder et al [34].

Another voltage-gated potassium current is carried by the Kv7.4 channel, which is encoded by the *KCNQ4* gene; it is a slow delayed rectifier, attested at the transcription level in MSMCs [13], conducting a slowly activating, non-inactivating current [96]. Miceli et al [96] studied the currents of various homomeric Kv7 channels in *Xenopus* oocytes, determining *V*_half_ = −12.1 mV and *k* = 12.7 mV. Temperature had a significant effect on those channels: increasing the temperature from 18°C to 28°C accelerated the activation and deactivation kinetics of the currents, as indicated by the parameter values *V*_half_ = −16.7 mV and *k* = 10.7 mV [96]. The current fits a double exponential function with *τ*_*a*_fast__ and *τ*_*a*_slow__ representing the time constants for the fast and slow components, respectively (Fig 30). The corresponding relative magnitudes did not change significantly with voltage, with the fast component accounting for ∼35% [34]. We postulated an additional ultrafast activation gate *d*_2_ to account for the extremely fast deactivation kinetics with deactivation time constant *τ*_*d*_deac__. The latter was determined by fitting an exponential function to the deactivation tail current elicited at −120 mV following steps to potential ranging from −40 mV to 40 mV [34]. The current is described by the following equation:

$$I_{\text{Kv7.4}}=\kappa_{\text{Kv7.4}}\;G_{\text{Kv7.4}}\;d_{1}\;d_{2}\;\left(V-E_{\text{K}}\right)\;,$$ (28)

where *d*_1_ and *d*_2_ represent the activation and deactivation gates, respectively, both with corresponding steady-state variable *d*_∞_.

#### Calcium-dependent potassium channel BK_*α*_

**Fig 31:**
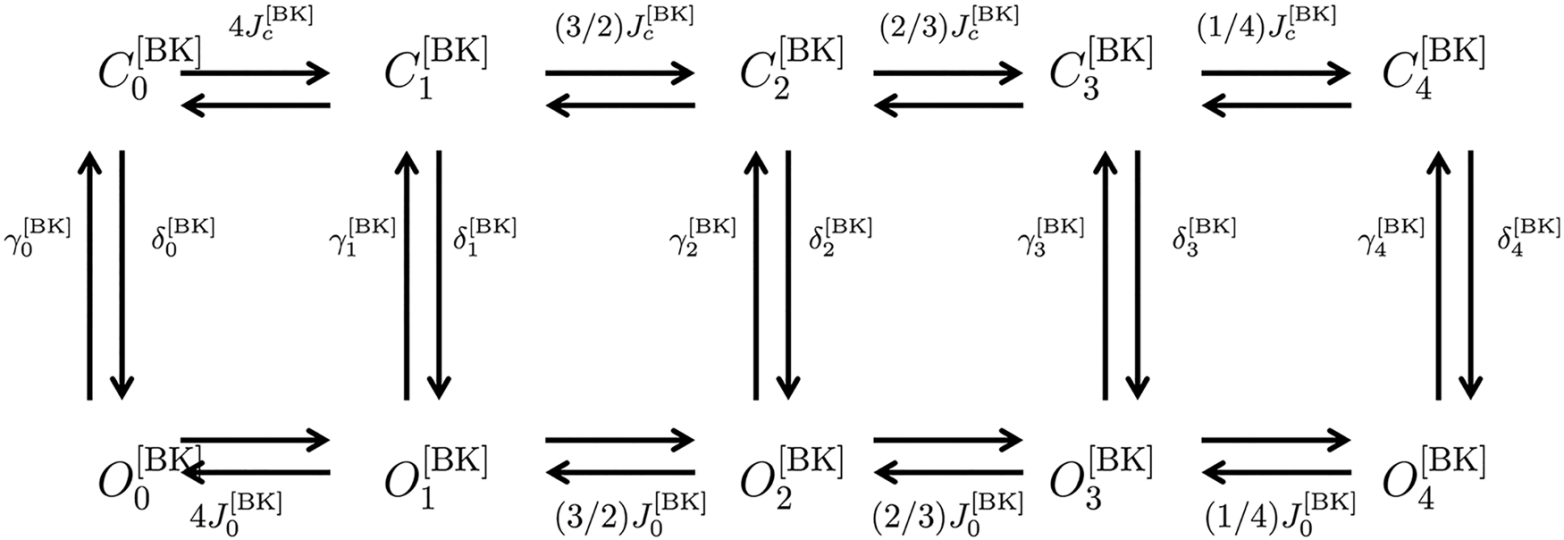
State transition diagram of the Markov model for the BK_*α*_ channel. State transition diagram of the Markov model for the BK_*α*_ channel. $C_{1}^{\text{[BK]}}$–$C_{4}^{\text{[BK]}}$ are the closed states and $O_{1}^{\text{[BK]}}$–$O_{4}^{\text{[BK]}}$ are the open states. Horizontal transitions represent voltage sensor movement while vertical transitions represent channel opening.

**Fig 32:**
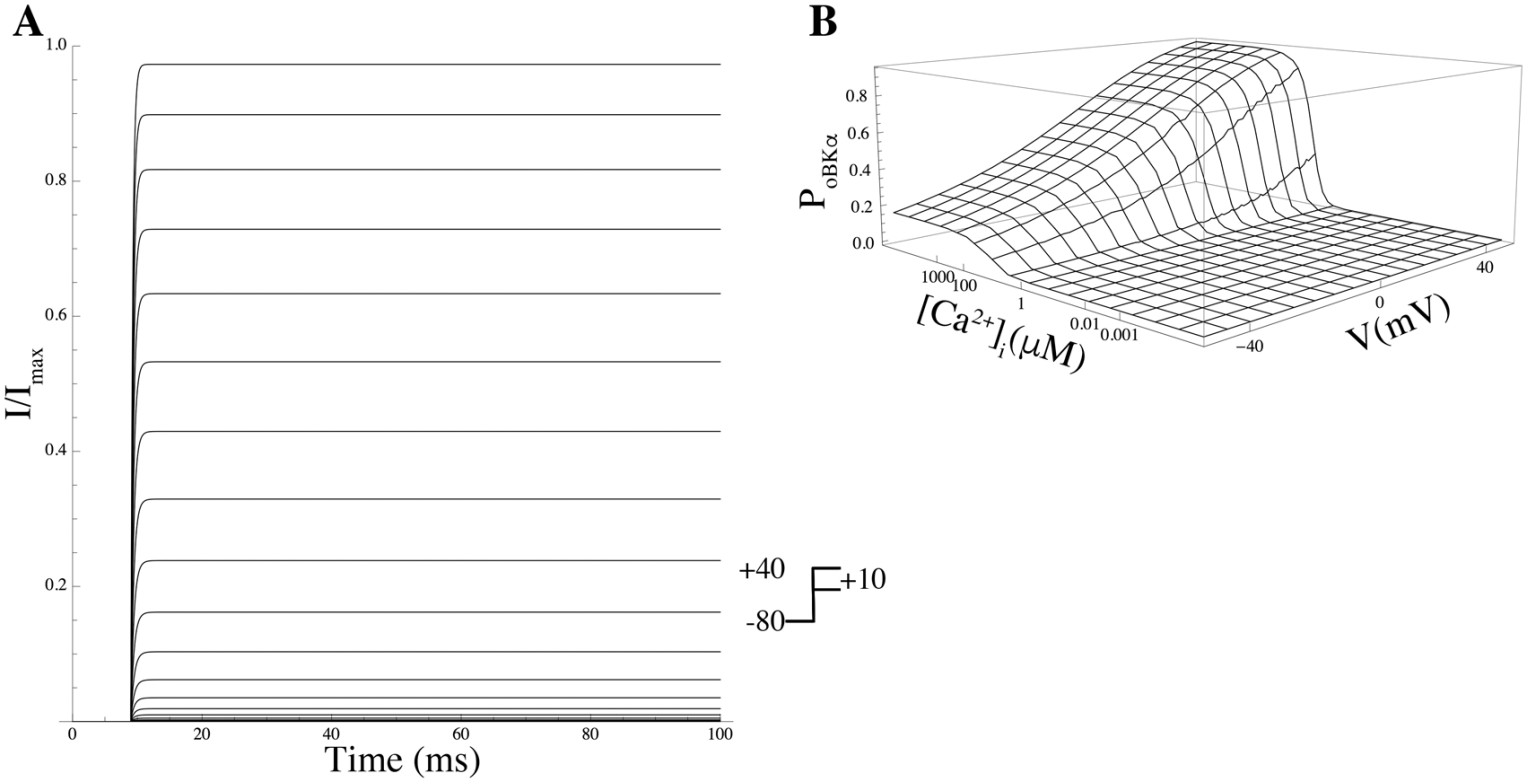
Properties of *I*_BK_*α*__. (A) Simulated voltage clamp traces of *α*-subunit BK channel from holding potential of −80 mV, where the voltage was stepped to values up to +240 mV in 10 mV increments. (B) Simulated open-channel probability plotted against [Ca^2+^]_*i*_ and the membrane potential.

The current *I*_BK_*α*__ is carried by the channel BK_*α*_, encoded by *KCNMA1* gene and forming the *α*-subunit of the large BK conductance; cloning and heterologous expression studies revealed that this channel’s *α*-subunit is a non-inactivating calcium-and voltage-sensitive channel, displaying a large single-channel conductance of around 260 pS [97–99]. At low cytosolic levels of Ca^2+^, *V*_half_ ≈ +125 mV, this parameter being lower at higher levels of Ca^2+^ [17], although the channel retains the capability of being fully activated by voltage alone [100]. At the mRNA level, BK_*α*_ is the most preponderant channel, both in the pregnant and the non-pregnant uterus [13]. An allosteric model, shown in Fig 31, was proposed by Horrigan et al [19]. Their model contains five open (O) and five closed (C) states that are arranged in parallel; the kinetics and steady-state properties of BK depend on the components associated with the transitions between these states, with horizontal transitions in the diagram representing movement of the channel’s four voltage sensors, each of which can be either active or inactive, and vertical transitions representing the conformational changes by which the channel opens or closes. This model is satisfactory in the absence of [Ca^2+^]_*i*_ [19]. To determine the calcium sensitivity of BK_*α*_, Bao et al [17] proposed that there are two groups of four high-affinity calcium binding sites, which are structurally distinct but have similar binding properties. This model could be combined with the allosteric model of Horrigan et al [19] to produce a model that considers both Ca^2+^ binding and voltage sensing; we found that this combined model gives an acceptable description of the channel’s behavior. The parameters were taken from Bao et al [17]. The voltage- and calcium-dependence of the channel’s open probability is plotted in Fig 32. The current is described by the following expression:

$$I_{\text{BK}{}_{\alpha }}=\kappa_{\text{BK}{}_{\alpha }}P_{o\text{BK}{}_{\alpha }}\left(V-E_{\text{K}}\right)\;,$$ (29)

where *P*_*o*BK*_α_*_ denotes the open-channel probability.

#### Calcium-dependent potassium channel BK_*α*+*β*1_

**Fig 33:**
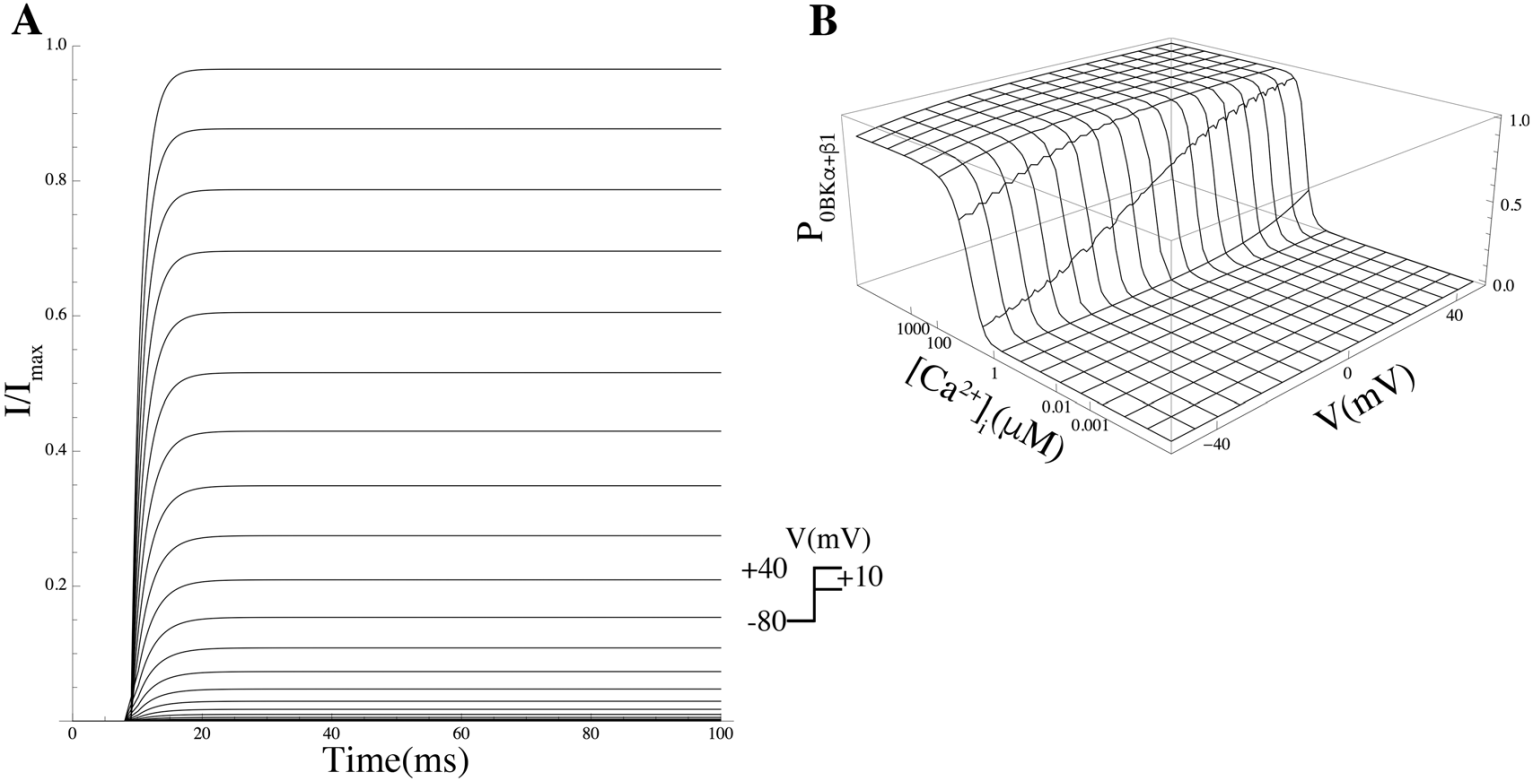
Properties of *I*_BK_*α*+*β*1__. (A) Simulated voltage clamp traces of BK_*α*+*β*1_ channel from holding potential of −80 mV, where the voltage was stepped to values up to +240 mV in increments of 10 mV, in the absence of calcium. (B) Simulated open-channel probability plotted against [Ca^2+^]_*i*_ and the membrane potential.

The *α*-subunit of the large BK conductance *KCNMA1* may combine with the *β*1-subunit (*KCNMB1*); the latter was found to be prominent at the mRNA level, both in the pregnant and the non-pregnant uterus [13]. The *β*1-subunit, when associated with the *α*-subunit, increases the calcium sensitivity and decreases the voltage-dependence of the channel; studies at the single-channel level indicated that the *β*1-subunit shifts the calcium dose-response curve of the BK channel leftward: at +30 mV, the affinity was 9.2 *μ*M and 2.6 *μ*M for BK_*α*_ and BK_*α*+*β*1_, respectively [17, 101, 102], with perhaps ∼80% of this shift due to voltage sensing and ∼20% due to calcium binding [17, 102]. In the absence of calcium, the *β*1-subunit increases the open-channel probability approximately 7 to 10-fold [17, 102]. The subunit has almost no effect on the equilibrium constants of the conformational change by which the BK channel opens and it does not affect the gating charge on the channel’s voltage sensors; it only affects the voltage sensor [17]. In particular, voltage sensor activation occurs at more negative voltages: at [Ca^2+^]_*i*_ = 1 *μ*M, *V*_half_ for activation in BK_*α*+*β*1_ was 82.1 mV as compared to 120.6 mV for BK_*α*_ [17]. We modeled the BK_*α*+*β*1_ channel following Horrigan et al [19], as we did with BK_*α*_. The gating kinetics is shown in Fig 33. The current is as follows:

$$I_{{\text{BK}}_{\alpha +\beta 1}}=\kappa_{{\text{BK}}_{\alpha +\beta 1}}P_{o{\text{BK}}_{\alpha +\beta 1}}\left(V-E_{K}\right)\;,$$ (30)

where *P*_*o*BK_*α*+*β*_1___ denotes the open-channel probability.

#### Calcium-dependent potassium channel BK_*α*+*β*3_

**Fig 34:**
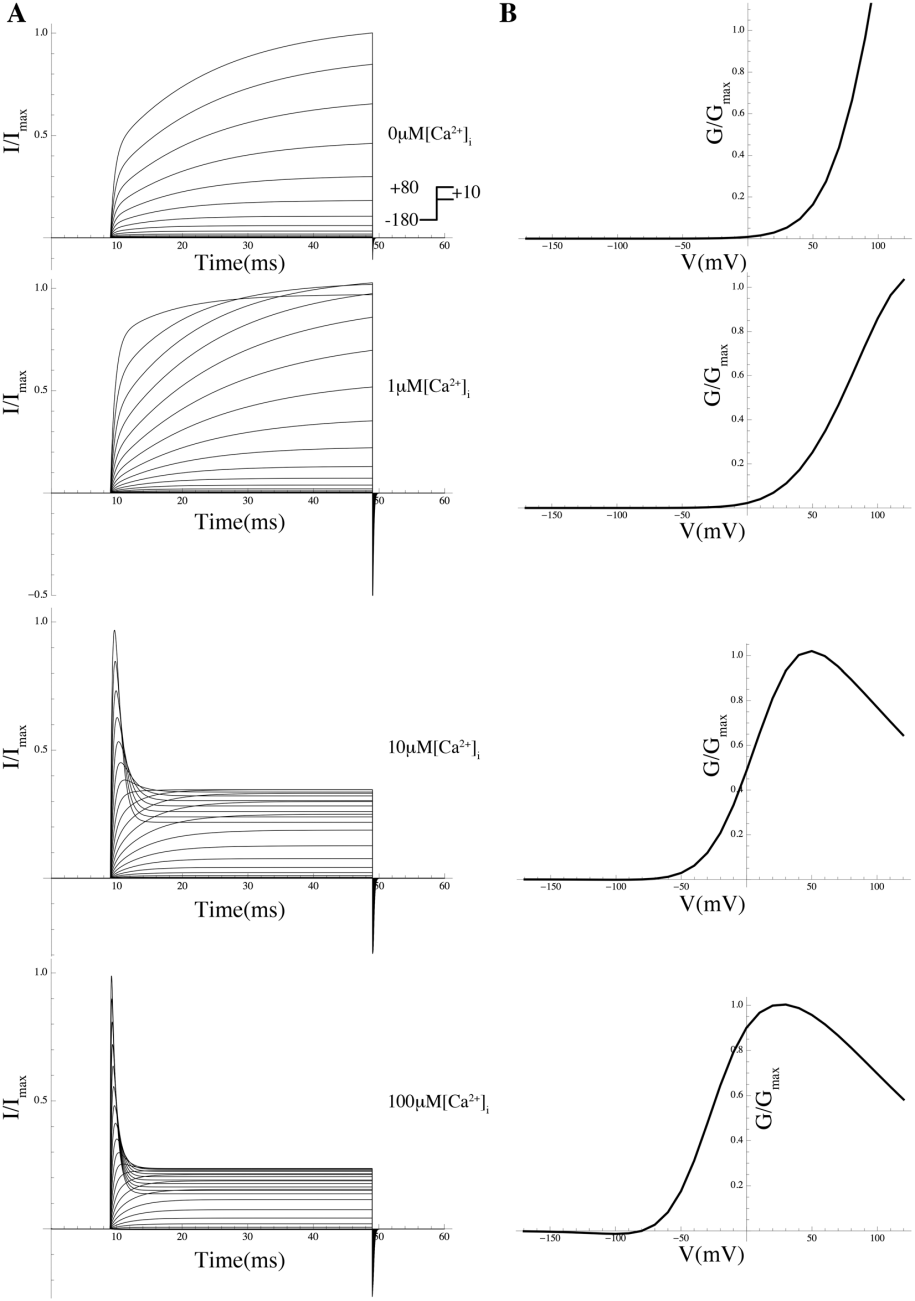
Properties of *I*_BK_*α*+*β*3__. (A) Simulated voltage clamp traces of BK_*α*+*β*3_ channel from holding potential of −180 mV, where the voltage was stepped to values up to +80 mV in 10 mV increments for various *μ*M [Ca^2+^]*_i_*, then stepped back to −180 mV (B) Simulated steady-state *I−V* relationship obtained from the series of voltage clamp experiments shown in (A). Values are normalised to the peak current values.

**Fig 35:**
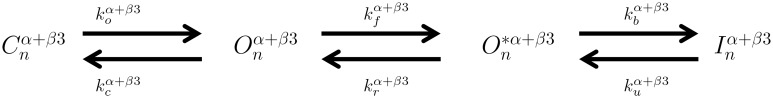
State transition diagram of the Markov model for the BK_*α*+*β*3_ channel. $C_{n}^{\left[\alpha +\beta 3\right]}$ is the closed state, $O_{n}^{\left[\alpha +\beta 3\right]}$ is the open state, and $I_{n}^{\left[\alpha +\beta 3\right]}$ is the inactivation state (n = 1…5); *k*_*f*_, *k*_*r*_, *k*_*b*_, and *k*_*u*_ denote transition rates between the states.

The combination of the BK_*α*_ subunit with the *β*_3_-subunit (*KCNMB3*) forms an entity which we denote as BK_*α*+*β*3_. The current observed when BK_*α*+*β*3_ was expressed in *Xenopus* oocytes was an inactivating current exhibiting both voltage-dependence and calcium-dependence, with activation displaced to more negative potentials as [Ca^2+^]_*i*_ was increased [18]. At strong depolarisation and higher [Ca^2+^]_*i*_, the current exhibits a rapid, if incomplete, inactivation [103], as shown in Fig 34. Above +50 mV, the current inactivates to a steady-state level that is 10–50% of the peak value, dependent on voltage, but largely independent of calcium [18]. Upon repolarisation, BK_*α*+*β*3_ recovers rapidly from inactivation [103]. Lingle et al [18] described the kinetics of BK_*α*+*β*3_ using the Markov model shown in Fig 35. The current mediated by the BK_*α*+*β*3_ channel is given by the following expression:

$$I_{{\text{BK}}_{\alpha +\beta 3}}=\kappa_{{\text{BK}}_{\alpha +\beta 3}}G_{{\text{BK}}_{\alpha +\beta 3}}P_{o{\text{BK}}_{\alpha +\beta 3}}\left(V-E_{\text{K}}\right)\;.$$ (31)

Inasmuch as the channel allows current in the ‘inactivated’ state (inactivation is incomplete) as well as the open state, the probability of being in the conducting state is described as follows:

$$P_{{\text{BK}}_{{\alpha +\beta 3}_{o}}}=\frac{O_{n}^{\left[\alpha +\beta 3\right]}+I_{n}^{\left[\alpha +\beta 3\right]}}{C_{n}^{\left[\alpha +\beta 3\right]}+O_{n}^{\left[\alpha +\beta 3\right]}+I_{n}^{\left[\alpha +\beta 3\right]}}\;.$$ (32)

The principle of microscopic reversibility is obeyed by the parameters of the BK Markov chain model.

#### Calcium-dependent potassium channel BK_*α*+*β*4_

**Fig 36:**
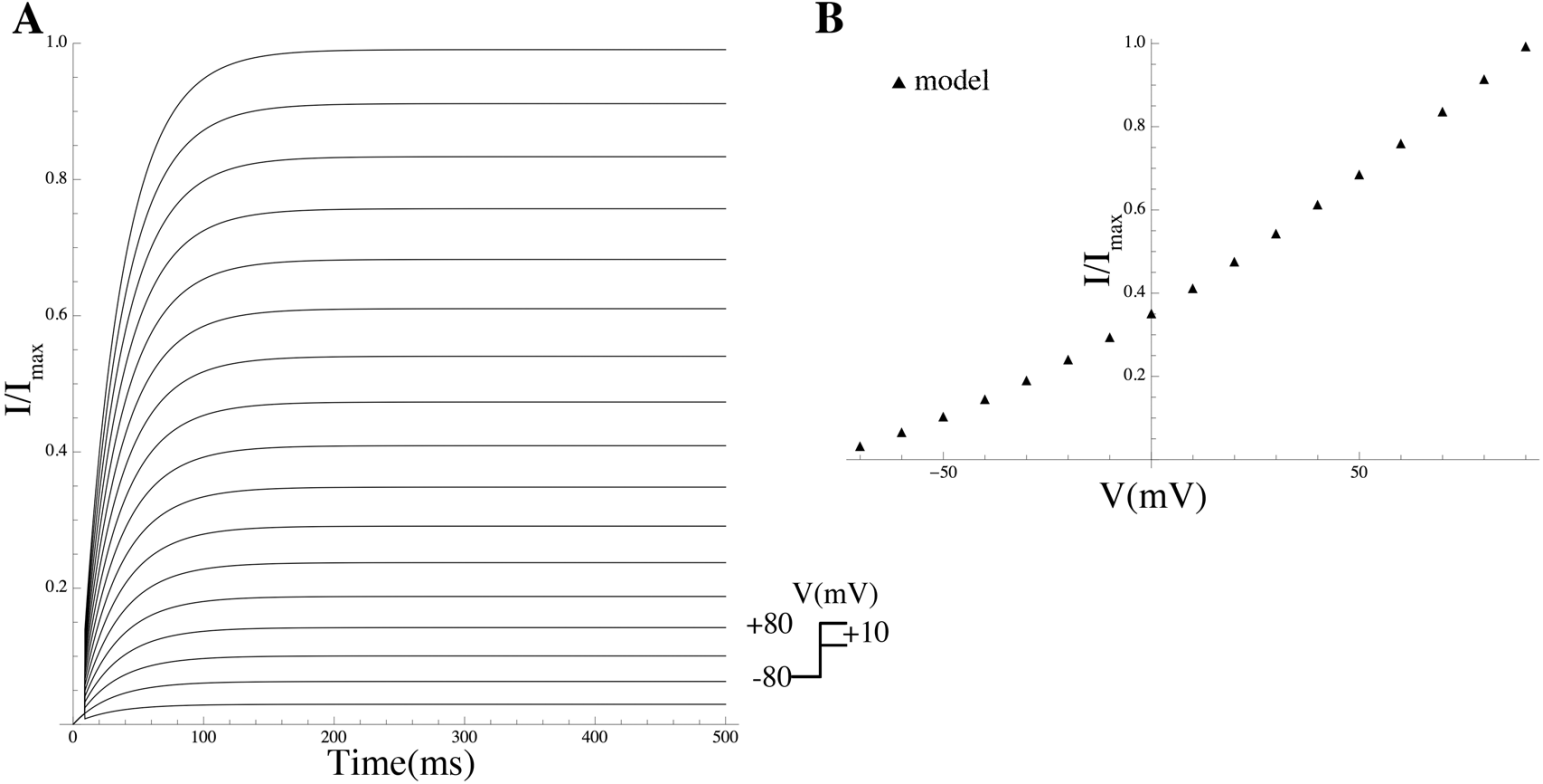
Properties of *I*_BK_*α*+*β*4__. (A) Simulated voltage clamp traces of BK_*α*+*β*4_ channel from holding potential of −80 mV, where the voltage was stepped to values up to +80 mV in 10 mV increments in 10 *μ*M [Ca^2+^]_*i*_ (B) Simulated peak *I*–*V* relationship obtained from the series of voltage clamp experiments shown in (A). Values are normalised to the peak current values.

The BK *α*-subunit can also associate with the *β*4-subunit (*KCNMB4*), which was attested at the mRNA level [13], and which modulates the biophysical characteristics of the *α*-subunit: activation is slowed by almost 30 ms [104], the activation range shifts to a range of more depolarised voltages [104], and unlike the BK channels discussed above, expression with the *β*4-subunit *decreases* calcium sensitivity at low cytosolic [Ca^2+^] and *increases* the sensitivity at high cytosolic [Ca^2+^] [20]. The most profound effect of the *β*4-subunit is a decrease of the open-channel probability by at least 11-fold, as compared to *α* by itself, in the absence of calcium binding and voltage sensor activation [105]. On the other hand, the subunit promotes the channel opening by sensitising the voltage-dependence of the open probability at negative membrane potentials. We modeled the BK_*α*+*β*4_ channel once more following Horrigan et al [19], using parameter values taken from Wang et al [20]. The current through this channel is given by the following expression:

$$I_{{\text{BK}}_{\alpha +\beta 4}}=\kappa_{{\text{BK}}_{\alpha +\beta 4}}P_{o{\text{BK}}_{\alpha +\beta 4}}\left(V-E_{\text{K}}\right),$$ (33)

where *P*_*o*BK_*α*+*β*4__ is the open-channel probability. Fig 36 summarises the kinetics of the BK_*α*+*β*4_ channel.

#### Calcium-dependent potassium channel SK_2_

**Fig 37:**
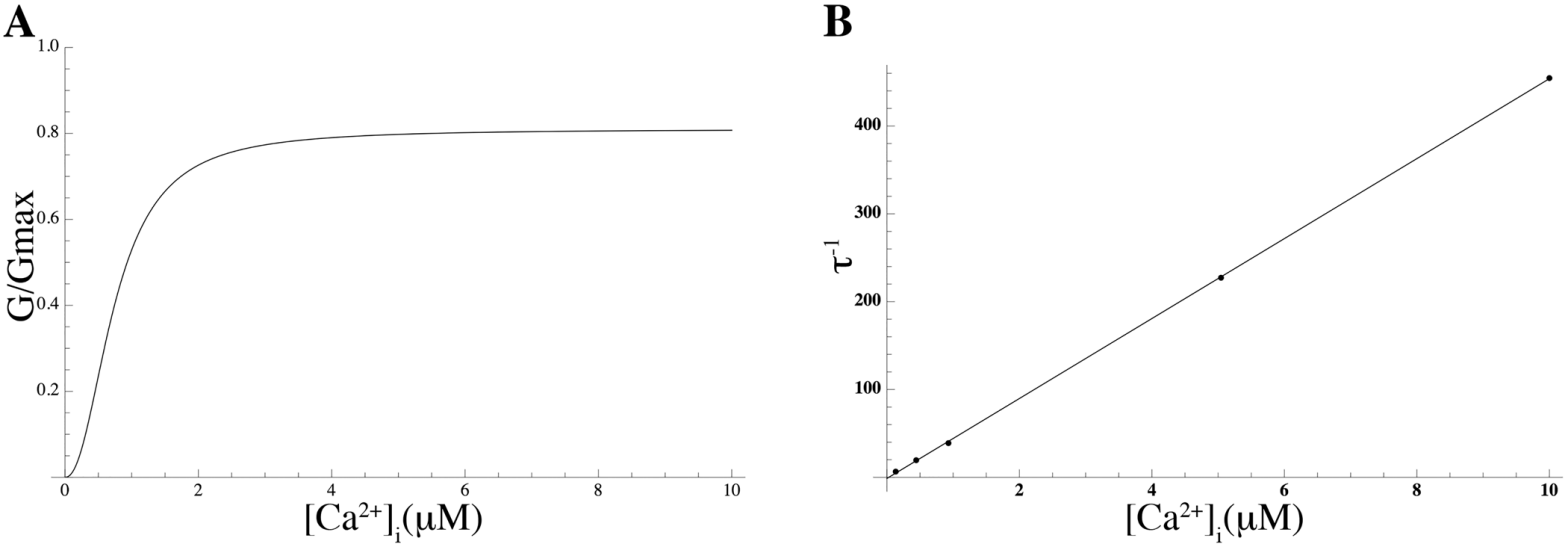
Properties of *I*_SK_2__. (A) Activation variable as a function of [Ca^2+^]_*i*_ obtained from experimental data of Hirschberg et al [22] as function of [Ca^2+^]_*i*_. (B) Activation time constant obtained from simulated current traces as function of [Ca^2+^]_*i*_.

The SK_2_ channel encoded by the gene *KCNN2* was attested at the mRNA level [13]; this is the second member of the SK family of low-conductance, calcium-activated, voltage-independent potassium channels. When macroscopic currents were recorded from *Xenopus* oocytes expressing rat SK_2_, the average open probability was found to be 0.42±0.12 at [Ca^2+^]_*i*_ = 0.6 *μ*M and 0.74±0.16 at [Ca^2+^]_*i*_ = 1 *μ*M [21]. The open probability as a function of [Ca^2+^]_*i*_ conforms to a Hill equation with EC_50%_ = 0.74 *μ*M and a Hill coefficient 2.2 [21, 22] (Fig 37). Hirschberg et al [22] expressed this channel in *Xenopus* oocytes and inferred the activation time constant; Fig 37 shows the activation rate (*τ*^−1^) as a function of [Ca^2+^]_*i*_. Over the range of 0.2 to 10 *μ*M [Ca^2+^]_*i*_, there is an approximately linear relationship between the activation rate of the macroscopic current and [Ca^2+^]_*i*_. We extracted the time constant from the Hirschberg et al [22] data, assuming the activation rate to be a linear function of [Ca^2+^]_*i*_. The SK_2_ current is given by the following expression:

$$I_{{\text{SK}}_{2}}=\kappa_{{\text{SK}}_{2}}G_{{\text{SK}}_{2}}P_{o{\text{SK}}_{2}}\left(V-E_{\text{K}}\right)\;,$$ (34)

where *P*_*o*SK_2__ denotes the open-channel probability, with corresponding steady state *P*_SK_2_*ss*_.

#### Calcium-dependent potassium channel SK_3_

**Fig 38:**
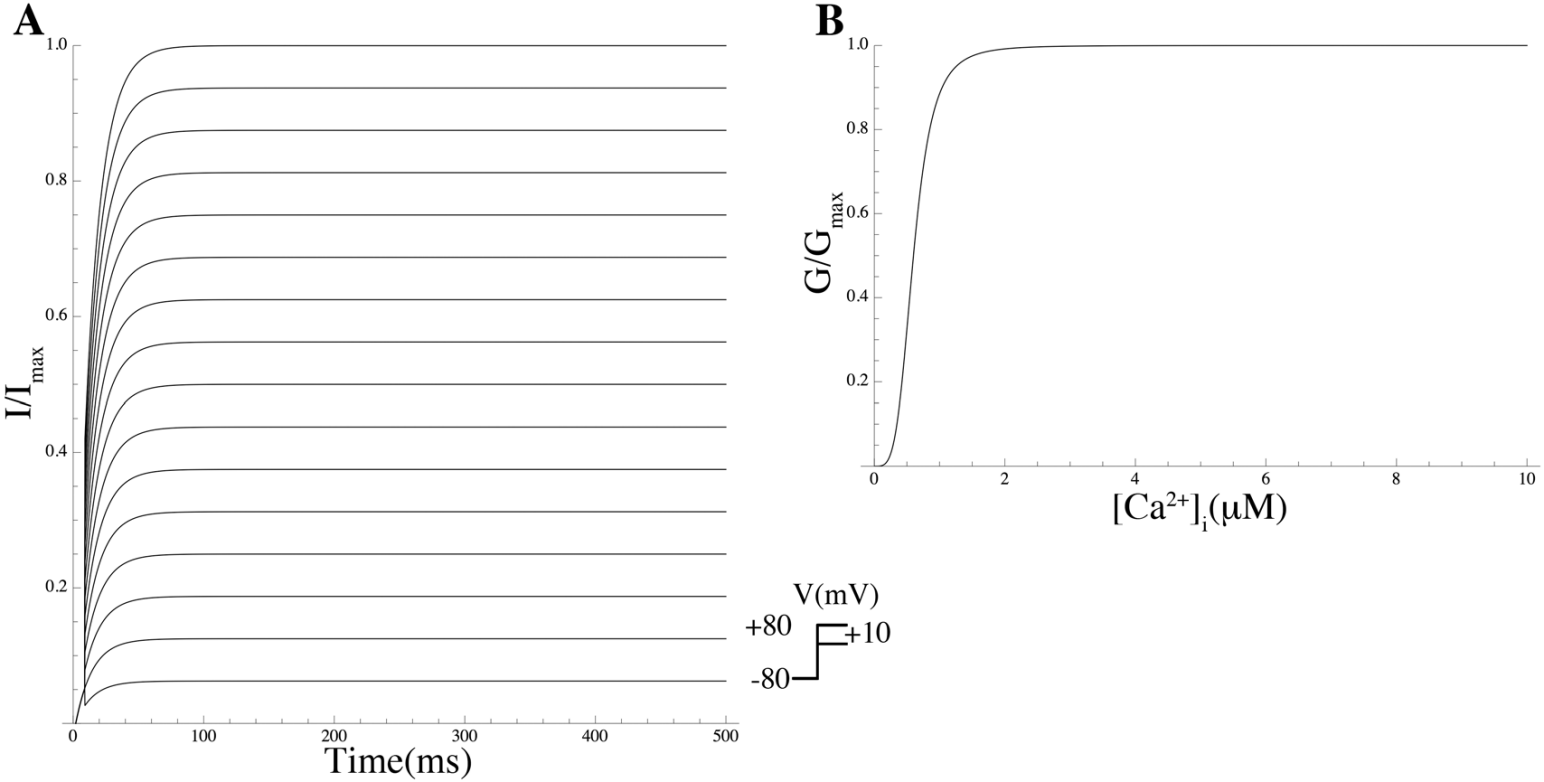
Properties of *I*_SK_3__. (A) Simulated voltage clamp traces of SK3 channel from holding potential of −80 mV, where the voltage was stepped to test potential up to +80 mV in 10 mV increments in 5 *μ*M [Ca^2+^]*_i_* (B) Activation variable obtained from experimental data of Xia et al [23] as function of [Ca^2+^]*_i_*.

The third member of the calcium-activated potassium channel is SK_3_, encoded by *KCNN3*; it is highly expressed at the mRNA level, with a significant difference between the pregnant and the non-pregnant uterus [13]. This channel may play a role in regulating uterine function by limiting the L-type calcium influx, thus contributing to a negative feedback that regulates [Ca^2+^]_*i*_ and consequently assisting relaxation of uterus and interrupting phasic contractions; furthermore, SK_3_ expression is depressed during labor, promoting L-type calcium channel activity [106]. Although it is known that SK_3_ is susceptible to voltage-dependent inactivation at voltages more positive than −40mV [107], we decided to ignore such inactivation in our model, since we could not find any relevant data. Calcium-dependent activation is voltage-independent and well-described by a Hill equation with EC_50%_ = 0.3 *μ*M and Hill coefficient 5 [23]. We assumed a single invariant activation time constant *τ*_SK_3_*a*_ = 12.9 ms (Fig 38). The current carried by SK_3_ is as follows:

$$I_{{\text{SK}}_{3}}=\kappa_{{\text{SK}}_{3}}G_{{\text{SK}}_{3}}P_{{\text{SK}}_{3}}\left(V-E_{\text{K}}\right)\;,$$ (35)

where *P*_SK_3__ represents the activation gate.

#### Calcium-dependent potassium channel SK_4_

The channel SK_4_ is also called the intermediate potassium channel IK1; it is activated via a Calmodulin-dependent mechanism [108]. It is considered to be a member of the SK family in view of shared properties, such as a relatively low conductance of about 11 pS (higher than the other SKs), weak dependence of activity on membrane potential, and calcium dependence. This channel has a high affinity for calcium with EC_50%_ = 95 nM [24]. The activation process is well-described by the Hill equation with Hill coefficient 3.2. We assumed a single invariant activation time constant *τ*_SK_4__ = 5.8 ms. The current carried by SK_4_ is given by the following expression:

$$I_{{\text{SK}}_{4}}=\kappa_{{\text{SK}}_{4}}G_{{\text{SK}}_{4}}P_{o{\text{SK}}_{4}}\left(V-E_{\text{K}}\right)\;,$$ (36)

where *P*_*o*SK_4__ is the activation variable.

#### Inward rectifier potassium channel Kir7.1

**Fig 39:**
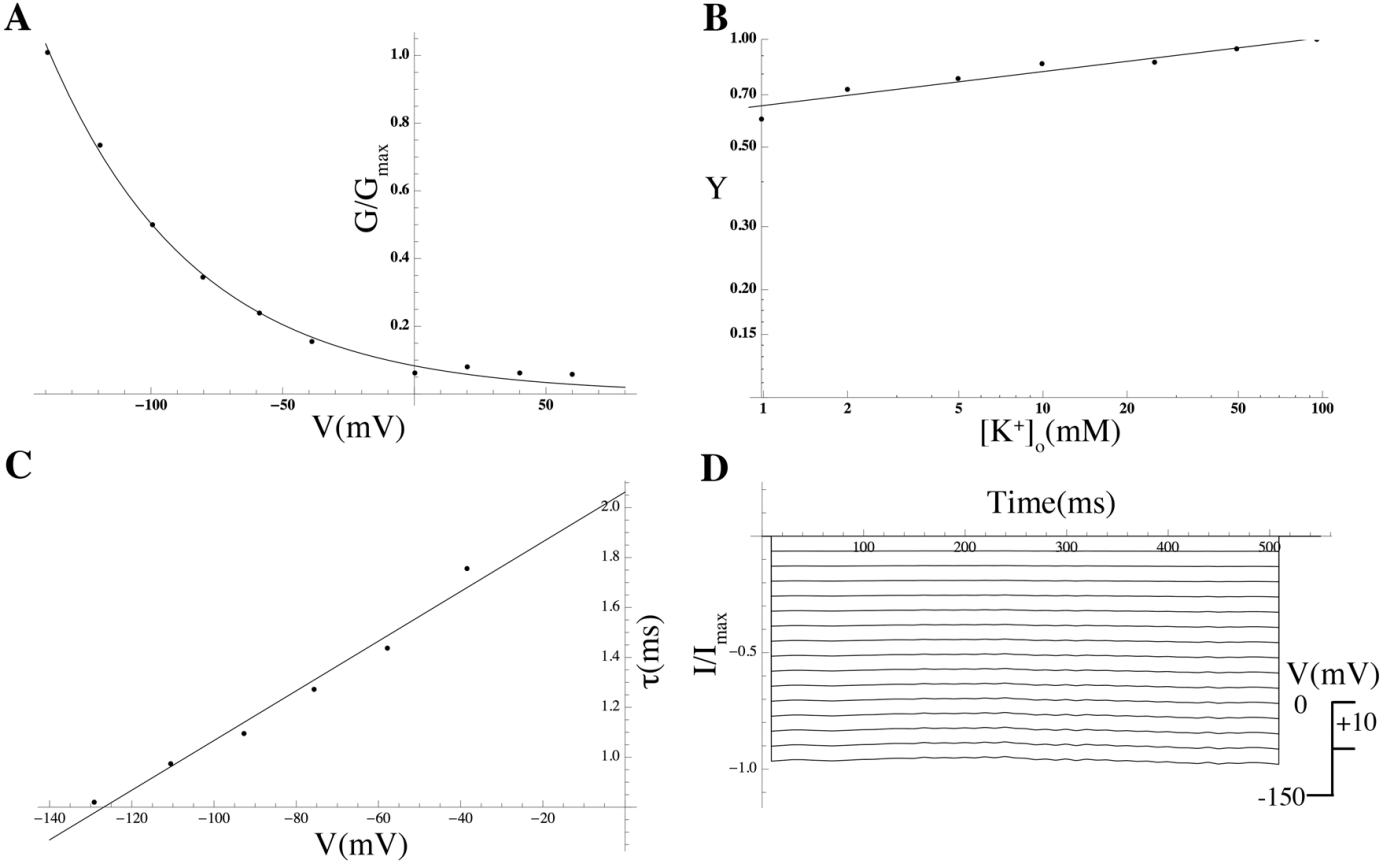
Properties of *I*_Kir7.1_. (A) Steady state activation curve from Doring et al [35] fit by a single exponential. (B) Double logarithmic plot for the Kir7.1 conductance as a function of [K^+^]_*o*_. (C) Activation time constant derived from experimental data (filled circles) from Doring et al [35]. (D) Current trace in simulated voltage-clamp experiments. Currents are recorded during 1 s voltage steps to potentials up to −150 mV from a holding potential of 0 mV. Values are normalised to the peak current values.

The channel Kir7.1, encoded by the *KCNJ13* gene, shares less than 37% homology with other Kir family members [35]; it has an extremely low single-channel conductance (∼ 50 fS) and inward rectification unaffected by the internal blocking particle [109]unusual pore properties that may be attributed to a group of amino acid residues that deviate from the corresponding conserved residues in all other Kir proteins [35, 109]. Doring et al [35] injected human Kir7.1 into *Xenopus* oocytes and observed a current that exhibited a weak dependence on [K^+^]_*o*_, again in contrast to other Kir channels. In view of these unique properties, the channel could not be modeled following the general example of the Kir family (see Fig 39B). Channel activation kinetics in response to hyperpolarising voltage pulses between −60 mV and −150 mV is rapid. The time constants of current activation were determined from single exponential fits (Fig 39). The *G*-*V* curve did not follow the Boltzmann function but was described by a single exponential (Fig 39). The current is given by the following expression:

$$I_{\text{Kir7.1}}=\kappa_{\text{Kir7.1}}\;G_{\text{Kir7.1}}\;P_{o\text{Kir7.1}}\;Y\;\left(V-E_{\text{K}}\right)\;,$$ (37)

where *P*_*o*Kir7.1_ the open-channel probability, and *Y* is a term to account for the dependence of the conductance on [K^+^]_*o*_, which was assumed to equal 5.4 mM [2] (Fig 39B).

#### Voltage-gated L-type calcium channel

**Fig 40:**
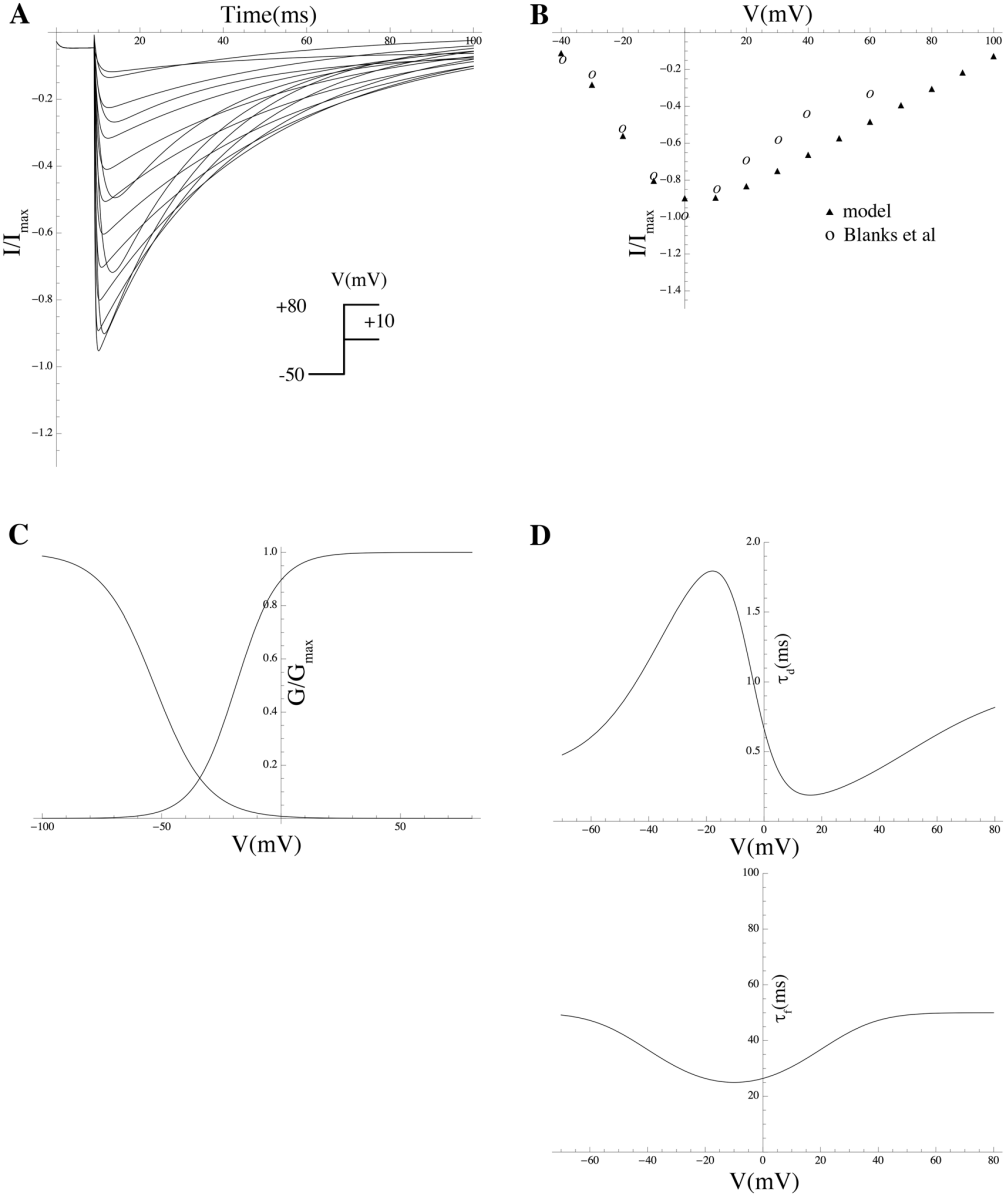
Properties of *I*_L-type_. (A) Normalised *I*_L-type_ current trace in simulated voltage-clamp experiments. Currents are recorded during 1 s voltage steps to potentials ranging from −50 to 80 mV from a holding potential of −50 mV. (B) Simulated (solid triangles) peak *I*–*V* relationship obtained from the series of experiments shown in (A). Values are normalised to the peak current values, data (circles) from Blanks et al [112]. (C) Steady state activation and inactivation curves. (D) Simulated activation and inactivation time constant.

The Ca_*v*_2.1 gene, also known as *CACNA1C*, encodes a member of the voltage-gated calcium channel family; it forms the main route for calcium entry into the MSMC and is distinguished by its robust and considerable unitary conductance [110]; L connotes ‘large’ and ‘lasting.’ Upon membrane depolarisation, this high-voltage-activated channel opens with a sigmoidal time course and subsequently inactivates. The current starts activating at around −50 mV and peaks at 0 mV [110]. The L-type channel is permeable to Ca^2+^, K^+^, and Na^+^ ions but we ignored the latter two because they do not vary significantly over the time scales of interest. The calcium reversal potential *E*_Ca_ varies in accordance with [Ca^2+^]_*i*_ as described by the Nernst equation. Whereas inactivation of the L-type channel is thought to be both calcium- and voltage-dependent, activation is strictly voltage-dependent [111]. A variety of gating characteristics has been reported; this may be due to different [Ca^2+^]_*o*_, holding potential or temperatures used in the experiments [2]. We based the steady-state voltage-dependent activation *d*_∞_ on the *I*–*V* curve found by Blanks et al [112], who performed experiments using human myometrial samples taken under term, preterm, labor, and not-in-labor conditions; the activation curve was described using Boltzmann equation with parameters *V*_half_ = −18.9 mV and *k* = 8.8 mV, values that closely match those reported by Hu et al [113], who expressed human *α*1*C*-subunit with *β*2*a*- and *α*2/*δ*- subunits in HEK 293 cells (*V*_half_ = −17 mV and *k* = 7 mV). A voltage-dependent steady-state inactivation variable *f*_∞_ was expressed by a Boltzmann function with *V*_half_ = −53 mV and *k* = −9.9 mV as reported in Shmigol et al [114]. We adopted the formula for activation time constant used in the Luo-Rudy model [3, 115], which was itself a modification of original work by Varghese and Sell [116]. This time constant *τ*_*d*_ is of the same order of magnitude as determined by Jones et al [117], who studied the calcium-activated chloride current in rat MSMCs. The inactivation time constant was taken from the Luo-Rudy model [3]. The formula for the calcium-dependent steady-state inactivation variable, *f*_Ca_, was adopted from the Ten Tusscher model [2]. Fig 40 summarises the gating kinetics. The L-type current is as follows:

$$I_{\text{Ltype}}=\kappa_{\text{Ltype}}\;G_{\text{Ltype}}\;d\;f\;f_{\text{Ca}}\left(V-E_{\text{Ca}}\right)\;,$$ (38)

where *d* and *f* are the activation and inactivation gating variables, respectively, with corresponding steady states *d*_∞_ and *f*_∞_, while *f*_Ca_ is the calcium-dependent inactivation variable.

#### Voltage-gated T-type calcium channel

**Fig 41:**
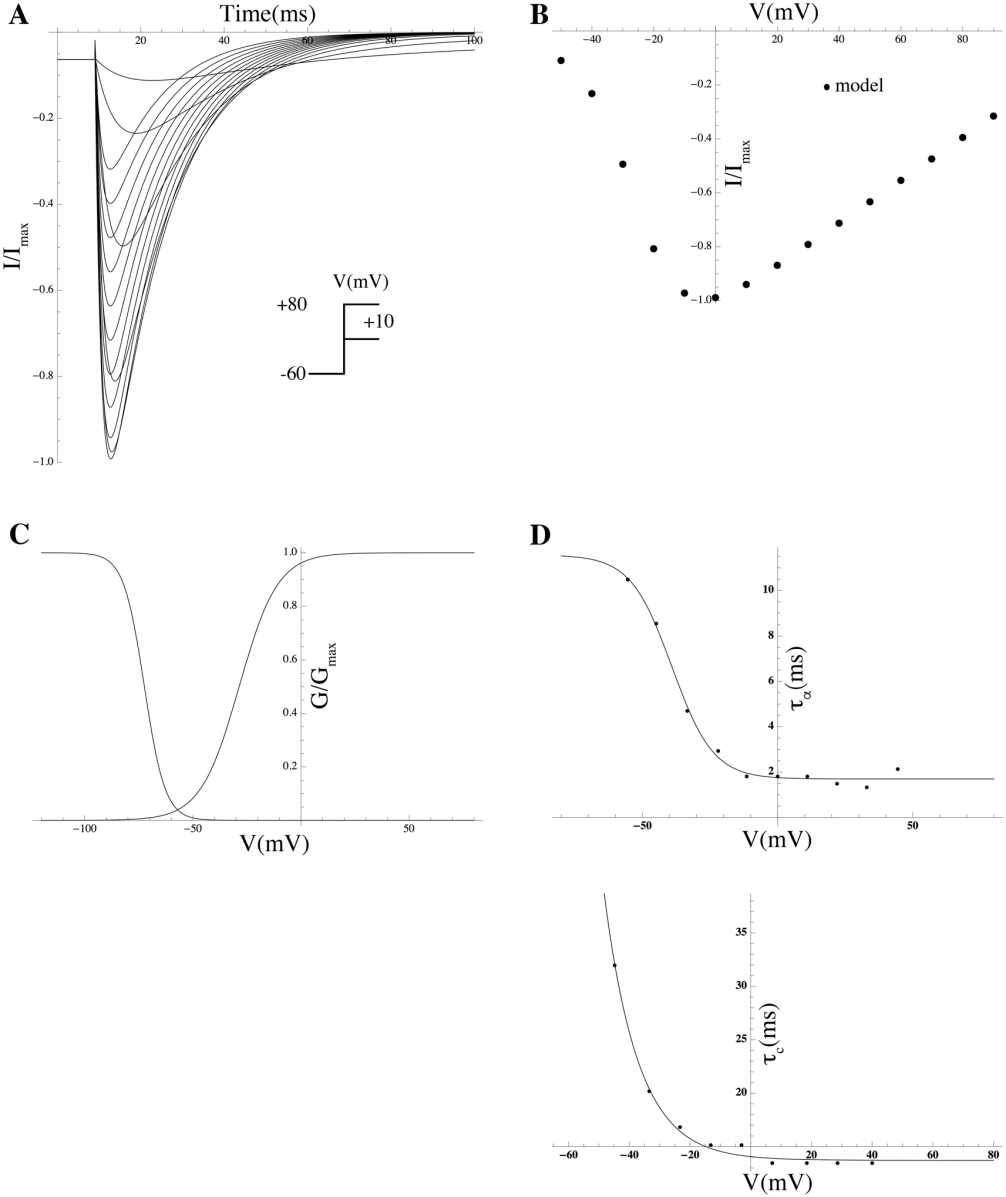
Properties of *I*_T-type_. (A) Normalised *I*_T-type_ current trace in simulated voltage-clamp experiments. Currents are recorded during 1 s voltage steps to potentials ranging from −60 to 80 mV from a holding potential of −60 mV. (B) Simulated (circles) peak *I*–*V* relationship obtained from the series of experiments shown in (A). Values are normalised to the peak current values. (C) Steady state activation and inactivation curves. (D) Simulated activation and inactivation time constant.

A subset of MSMCs expresses *CACNA1G*, which encodes the *α*1G-subunit of the T-type calcium channel Ca_*v*_3.1 [112]. In contrast to the L-type channel, the T-type calcium current is tiny and transient, with a relatively small unitary conductance of 7.5 pS [26], and is activated at potentials ranging from −50 mV to −30 mV, beginning to open after small depolarisations (10 mV) and displaying fast inactivation [118]. Although this channel contributes to the rise in [Ca^2+^]_*i*_, its main role remains unclear [112]. Perez-Reyes et al [26] transfected HEK-293 cells with rat *α*1G; their data conform to the Boltzmann equation with parameter values *V*_half_ = −28.6 mV, slope 8.9 mV for activation and *V*_half_ = −72.4 mV, slope = −4.8 mV for inactivation. Time constants of activation and inactivation were calculated from exponential fits to the current traces obtained during test pulses. The properties of the T-type channel are shown in Fig 41. The current through the T-type channel is given by the following expression:

$$I_{\text{T-type}}=\kappa_{\text{T-type}}\;G_{\text{T-type}}\;a\;c\;\left(V-E_{\text{Ca}}\right)\;,$$ (39)

where *a* and *c* are the activation and inactivation variables with corresponding steady states *a*_∞_ and *c*_∞_.

#### Calcium-dependent chloride channel CaCC

**Fig 42:**
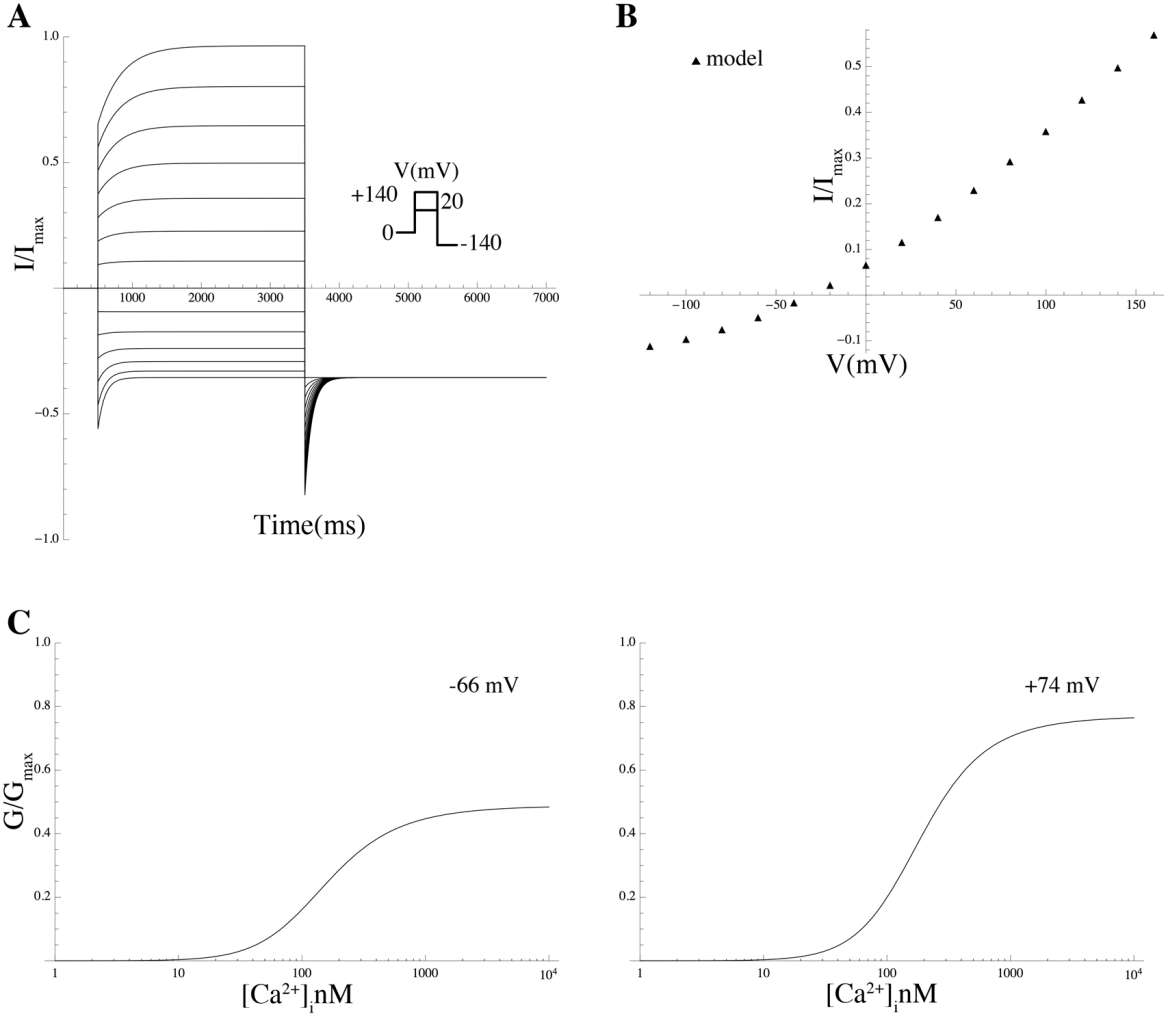
Properties of *I*_CaCC_. (A) Normalised *I*_CaCC_ current trace in simulated voltage-clamp experiments. Currents are recorded during 1 s voltage steps to potentials ranging from −120 to 140 mV from a holding potential of 0 mV then followed by a repolarising pulse to −140 mV. (B) Simulated steady-state *I*–*V* relationship obtained from the series of experiments shown in (A). Values are normalised to the peak current values. (C) [Ca^2+^]_*i*_-dependence of CaCC channel activation. Conductance as a function of [Ca^2+^]_*i*_ at −66 mV and +74 mV, respectively.

The calcium-activated chloride channel in MSMCs is encoded by the gene *ANNO1* which is attested by the mRNA expression data [13]. It is voltage- and calcium-dependent: elevated [Ca^2+^]_*i*_ levels induce an increase in the Cl^−^ current, irrespective of membrane potential, and the steady-state *I*–*V* relationship exhibits outward rectification at low [Ca^2+^]_*i*_ [27]. We adopted the model proposed by Arreola et al [27]; the calcium-dependence of the steady-state channel activation at various membrane potentials was modeled by the Hill equation. The dissociation constant K_*d*_ and the Hill coefficient were both taken to be functions of the membrane potential. Fig 42 shows the relative conductance obtained at membrane potentials of −66 mV and +74 mV for different [Ca^2+^]_*i*_, with K_*d*_ values 360 nM and 73 nM, respectively, and Hill coefficients 1.2 and 2.3, respectively. The chloride current is given by the following expression:

$$I_{\text{CaCC}}=\kappa_{\text{CaCC}}\;G_{\text{CaCC}}\;c_{c}\;\left(V-E_{\text{Cl}}\right)\;,$$ (40)

where *c*_*c*_ is an activation gating variable. This model was based on whole-cell recordings of macroscopic currents.

#### Gap junctions

**Fig 43:**
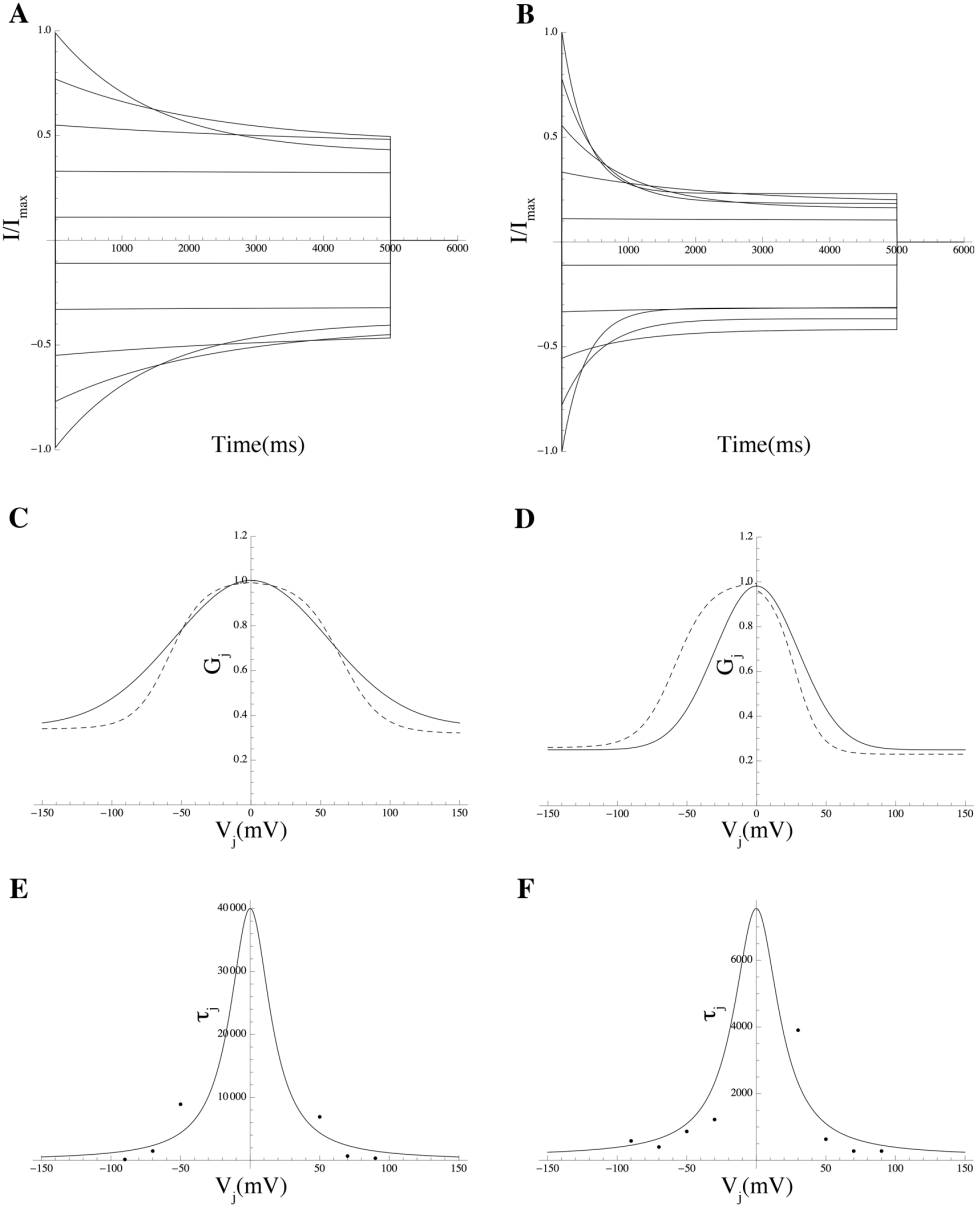
Properties of the gap junctions in MSMC. Simulated currents for Type I and Type II are shown in (A) and (B), respectively. The currents are elicited in response to 5 s square pulses in the voltage range of ± 90 mV from holding potential of 0 mV. Steady-state conductance as function of *V*_*j*_ for Type I an Type II shown in (C) and (D), respectively, using quasi-symmetrical Boltzmann functions (dashed line) and the Gaussian function (solid line). The time constant as function of *V*_*j*_ for Type I and Type II are shown in (E) and (F), respectively.

Gap junctions form a passage between adjacent cells, providing a direct pathway for electrical and metabolic signalling between cells [119–122]. The connexin proteins of the gap junctions belong to a gene family that comprises around 20 members; connexin-43 has been identified as the principal protein of human myometrial gap junctions [123, 124]. The gap junctions between MSMCs increase dramatically during the first stages of pregnancy: over 200-fold during the last 12 hours of pregnancy in rat MSMCs [36, 125]. Miyoshi et al [36] used the double-whole-cell voltage-clamp approach to study gap junctions between freshly isolated pairs of cells from rat myometrium; the macroscopic gap junction currents decayed slowly from an instantaneous, constant-conductance level to a steady-state level (dependent on the transjunctional voltage *V*_*j*_) given by eq (41) below. Miyoshi et al [36] reported two distinct types of voltage-dependence, called Type I and Type II as shown in Fig 43. Type I closely resembles currents observed in other systems where connexin-43 predominates, whereas Type II exhibited a marked voltage-dependence that is similar to the currents associated with connexin-45 [126]. In our model, we incorporated two different entities to account for both types of gap junction populations; the junctional conductances were 85 pS for Type I [36] and 30 pS for Type II [126]. The normalised (dimensionless) conductances as a function of the transjunctional potential difference *V*_*j*_ at the steady state were described by the following equation:

$$G_{j}=\frac{1-G_{\text{min}}}{1+\exp\{-A\left(V_{j}-V_{h}\right)\}}+G_{\text{min}}\;,$$ (41)

where *G*_*j*_ is the normalised value of junctional conductance, *G*_min_ is the minimum value of *G*_*j*_, *V*_*h*_ denotes the half inactivation voltage and *A* is a slope factor ((mV)^−1^). We used the following expressions to describe *G*_*j*_:

$$G_{j}\text{(Type}\;\text{I)}=0.35+\frac{90}{\sqrt{2\pi \sigma^{2}}\;\exp\left\{\frac{-V_{j}^{2}}{2\sigma^{2}}\right\}}\;,$$ (42)

$$G_{j}\text{(Type}\;\text{II)}=0.25+\frac{55}{\sqrt{2\pi \sigma^{2}}\;\exp\left\{\frac{-V_{j}^{2}}{2\sigma^{2}}\right\}}\;,$$ (43)

where *σ* = 55 for Type I and *σ* = 30 for Type II. The junctional current was instantaneously activated, then inactivated in a voltage-dependent manner to a steady-state level, and inactivation was incomplete even at the largest test voltages [36]. The inactivation time constant as function of *V*_*j*_ was extracted from data taken from Miyoshi et al [36]. We modeled the decline as a first-order process; the decay time constant was found to depend on voltage as shown in Fig 43. We described the time constants as a function of *V*_*j*_ (see Supplementary Materials). We used the following model for the gap-junctional current:

$$I_{\text{GJ}}=\kappa_{\text{GJ}}\;G_{\text{GJ}}\;P_{\text{GJ}}\;\left(V_{j}\right)\;,$$ (44)

where *P*_GJ_ is the inactivation variable with steady-state *G*_*j*_ and time constant *τ*_*j*_. If the cell under investigation is the driving cell (the pacemaker cell), the following expression for the driving force can be assumed:

$$V_{j}=V\left(t\right)-V\left(t+t_{d}\right)\;,$$ (45)

where *t*_*d*_ is an effective delay time (a constant parameter). From a physics point of view, this is reasonable provided that the waveform passing through the system is similar for the driving cells and the adjacent cells.

#### Potassium background current

The K^+^ background or ‘leak’ current is a collective term for currents that are carried by a variety of ohmic (or quasi-ohmic) transmembrane pores [1]; mRNA expression data indicate that background potassium channels are abundantly expressed in MSMCs [13]. The leak current is important in maintaining a negative resting potential and counterbalancing depolarising drives [1]. One prominent potassium channel that carries background current is Kv7.1/KCNE3; when modulated by the KCNE3 subunit, which is abundantly expressed at the mRNA level [13], this channel loses its voltage-dependence gating and produces an instantaneous, nearly ohmic current [93]. Furthermore, three members of the two-pore domain (K2P) family were attested at the mRNA level in MSMCs [13]: (i) TWIK1, encoded by *KCNK1*, also known as K2P1.1; (ii) TASK1, encoded by *KCNK3*, also known as K2P1.3; and (iii) TWIK2, encoded by *KCNK6*, also known as K2P6.1. These channels are affected by non-voltage factors such as pH, temperature, and deformation of the cell membrane; each subunit of the K2P contains two K^+^ channel pore loops, forming domains with four transmembrane segments (reviewed by Enyedi et al [127]). In our model, a simple ohmic expression was used to describe the sum total of these currents:

$$I_{\text{bgK}}=\kappa_{\text{bgK}}G_{\text{bgK}}\left(V-E_{\text{K}}\right)\;.$$ (46)

Parameter values are listed in the Supplementary Materials.

#### Chloride background current

An ohmic background chloride channel was also added to the model, with the following expression for the current:

$$I_{\text{bgCl}}=\kappa_{\text{bgCl}}G_{\text{bgCl}}\left(V-E_{\text{Cl}}\right)\;.$$ (47)

Parameter values are listed in the Supplementary Materials.

#### Plasma membrane Ca-ATPase

An MSMC maintains a relaxed state largely by ATP-dependent extrusion of calcium from smooth muscle cells [128]; at rest, [Ca^2+^]_*i*_ in MSMCs is kept at 100–200 nM which is extremely low, compared to the high extracellular concentration of about 1–2 mM [110]. The plasma membrane Ca-ATPase (PMCA) is an ATP-dependent transport protein that removes Ca^2+^ from the intracellular milieu against a steep concentration gradient; one calcium ion is removed for every hydrolysed molecule of ATP [1]. Expression of plasma membrane Ca-ATPase has been attested in both rat and human MSMSc [128–132]. The Ca-ATPase isolated from smooth muscle plasma membrane closely resembles the calcium pump ATPase of the heart plasma membrane [133]. Therefore, we adopted the Luo-Rudy model for the cardiac myocyte which features a Hill equation with saturation constant 0.0005 mM and a unity Hill coefficient [3]:

$$I_{\text{PMCA}}=\kappa_{\text{PMCA}}\;\frac{1.15}{1+0.0005/{\left[{\text{Ca}}^{2+}\right]}_{i}}\;,$$ (48)

where *I*_PMCA_ is the current density in pA/pF. Because of the lack of data regarding the unitary conductance, we adapted a model with currents expressed in pA/pF; *κ*_PMCA_ is a dimensionless channel density factor that is estimated by the procedure outlined in “Computational Methods” above.

#### Na^+^/K^+^-ATPase

During each cycle, Na^+^/K^+^-ATPase transfers three Na^+^ ions out of the cell and two K^+^ ions into the cell, which helps to maintain the resting membrane potential and the standing gradient of the sodium and potassium ions. The protein has been attested in both rat and human MSMCs [134, 135]. On the time scales involved, the associated Na^+^ and K^+^ currents do not appreciably affect the ionic content of the cell or its environment; the values of the intracellular and extracellular ionic concentrations are listed in S1 Data. In view of a lack of experimental data that describe the pump activity in myometrium, we adopted the Luo-Rudy cardiac myocyte model [3] for the Na^+^/K^+^-ATPase current:

$$I_{\text{NaK}}=\kappa_{\text{NaK}}I_{{\text{NaK}}_{\text{max}}}f_{\text{NaK}}I_{\text{NaK,nai}}I_{\text{NaK,ko}}\;,$$ (49)

where the dependence of the pump on [Na^+^]_*i*_ and [K^+^]_*o*_ is described using the Hill equation:

$$I_{\text{NaK,nai}}=1/\left(1+{\left(10/{\left[{\text{Na}}^{+}\right]}_{i}\right)}^{2}\right)\;,$$ (50)

$$I_{\text{NaK,ko}}=1/\left(1+1.5/{\left[{\text{K}}^{+}\right]}_{o}\right)\;.$$ (51)

Since the unitary conductance is not known, we adapted a model with currents expressed in pA/pF; *κ*_NaK_ is a dimensionless channel density factor that is estimated by the procedure outlined in “Computational Methods” above. The effects of membrane potential and [Na^+^]_*o*_ are represented by the following functions:

$$f_{\text{NaK}}={\left(1+0.1245\;\exp\{-0.1VF/RT\}+0.0365\;\sigma \;\exp\{VF/RT\}\right)}^{-1}\;,$$ (52)

$$\sigma ={\left(7(\exp\left\{{\left[{\text{Na}}^{+}\right]}_{o}/67.3\right\}-1)\right)}^{-1}\;,$$ (53)

where *I*_NaK_ represents the current density in pA/pF.

#### Na^+^/Ca^+^ exchanger

The Na^+^/Ca^+^ exchanger (NCX) is a transmembrane protein that removes calcium ions from the cell, thus assisting in the creation and maintenance of Na^+^ and Ca^2+^ concentration gradients across the cell membrane. The exchanger been identified in uterine cells and mediates a calcium influx at the expense of sodium efflux; the stoichiometry is 1 Ca^2+^ for every 3 Na^+^ [130, 136]. It has low affinity but a high capacity for calcium ions and accordingly, its contribution is thought to reside mainly in the regulation of elevated cytosolic Ca^2+^ levels, in contrast to PMCA, which extrudes calcium at lower [Ca^2+^]_*i*_ thus providing “fine tuning” of resting [Ca^2+^]_*i*_ levels [137]. As before, we adapted the Luo-Rudy model [3]. The NCX current is given by the following expression:

$$I_{\text{NCX}}=\kappa_{\text{NCX}}\left(0.00025e^{-0.65VF/\left(RT\right)}\frac{e^{VF/\left(RT\right)}{\left[{\text{Na}}^{+}\right]}_{i}^{3}{\left[{\text{Ca}}^{2+}\right]}_{o}-{\left[{\text{Na}}^{+}\right]}_{o}^{3}{\left[{\text{Ca}}^{2+}\right]}_{i}}{1+0.0001{\left[{\text{Na}}^{+}\right]}_{i}^{3}{\left[{\text{Ca}}^{2+}\right]}_{o}+{\left[{\text{Na}}^{+}\right]}_{o}^{3}{\left[{\text{Ca}}^{2+}\right]}_{i}}\right)\;,$$ (54)

where *I*_NCX_ is the current density in pA/pF. Because of the lack of data regarding the unitary conductance, we adapted a model with currents expressed in pA/pF, where *κ*_NCX_ is a dimensionless channel density factor that is estimated by the procedure outlined in “Computational Methods” above.

#### Calcium dynamics

To represent the MSMC calcium dynamics, we developed a minimal model. Although the rise in [Ca^2+^]_*i*_ can be augmented by the release from the stores, calcium entry from the extracellular space is the major source of calcium-triggered contractions [43]. Therefore, the model only considers the increase of [Ca^2+^]_*i*_ due to the plasma membrane calcium channels (L-type and T-type), while ignoring Ca^2+^ release from internal stores. To determine the parameter values, [Ca^2+^]_*i*_ has to be related to the signal that is experimentally observed, i.e. *f*/*f*_0_. Further details on the acquisition of the *f*/*f*_0_ signal were given in a foregoing section on experimental methods. The cytosolic calcium concentration is related to the fluorescence intensity *f* by the following equation:

$${\text{[Ca}}^{2+}{\text{]}}_{i}=K_{d}\frac{\left(f-f_{\text{min}}\right)}{\left(f_{\text{max}}-f\right)}$$ (55)

where *K*_*d*_ is the apparent calcium-binding affinity of the calcium indicator (Fluo-4), and *f*_min_, *f*_max_ are the minimum and maximum fluorescence intensities, respectively. We used the following differential equation to account for the biophysical processes that affect cytosolic calcium dynamics:

$$\frac{d{\text{[Ca}}^{2+}{\text{]}}_{i}}{dt}=\gamma_{L}\;I_{\text{L-type}}+\gamma_{T}\;I_{\text{T-type}}-\lambda \;{\text{[Ca}}^{2+}{\text{]}}_{i}$$ (56)

where *I*_L-type_ and *I*_T-type_ are the voltage-activated calcium currents from the L-type and the T-type channels (resulting in calcium entry), respectively. The corresponding coefficients are *γ*_*L*_ and *γ*_*T*_. The term *λ* [Ca^2+^]_*i*_ represents the recovery from excitation. The removal of calcium proceeds via calcium extrusion caused by the existing pumps or due to binding to the intracellular calcium buffers. We used the observed time series of the membrane potential as a forcing function to drive the voltage-gated calcium channels. The simultaneous calcium fluorescence signal (see Materials and Methods) was used to find the least-squares fit of the calcium excitation model (Fig 2). The parameters of the model *K*_*d*_, *f*_max_, *γ*_*L*_, *γ*_*T*_, *λ*, and the steady state [Ca^2+^]_*i*_ (*f*_min_ = 0 for Fluo-4) were determined by means of least-squares estimation. The estimated parameter values are shown in Table 4. Although our model is a single-variable model and does not take into account calcium release from internal stores (via IP_3_ receptors and RyR), it was able to reproduce experimental data quite accurately, with a small number of free parameters.

**Table 4:** Calcium dynamics parameter values.

Parameter	Definition	Value
*K*_*d*_	Apparent calcium-binding affinity of the calcium indicator (Fluo-4)	1050 nM
*f*_max_	Maximum fluorescence intensity	4520
*γ*_*L*_	L-type current coefficient	2251
*γ*_*T*_	T-type current coefficient	1.38
*λ*	Coefficient for recovery from excitation	0.00063
Ca_in_	Initial [Ca^2+^]_*i*_	60.7 nM

## Supporting Information

S1 Model Mathematica code for the free-running model. (NB)

S2 Model Mathematica code for the parameter estimation. (NB)

S1 Equations Model parameters and equations. (PDF)

S1 Data Voltage and intracellular calcium trace for parameter estimation. (XLS)

S2 Data Raw voltage-clamp traces underlying the validation data reported in Fig 3. (XLSX)

S1 Fig Left: breakdown of the three Kv2.1 oligomeric complexes (Kv2.1/6.1, Kv2.1/9.3, Kv2.1) that contribute to the total current depicted in Fig 3. Right: comparison of hERG current as simulated and observed. (TIF)

## References

[1] Hille B (2001) Ion Channels of Excitable Membranes, Third Edition Sinauer Associates, Inc.

[2] ten Tusscher K, Noble D, Noble PJ, Panfilov AV (2004) A model for human ventricular tissue. Am J Physiol Heart Circ Physiol 286: H1573–H1589. 14656705 10.1152/ajpheart.00794.2003

[3] Livshitz L, Rudy Y (2009) Uniqueness and stability of action potential models during rest, pacing, and conduction using problem-solving environment. Biophys J 97: 1265–1276. 10.1016/j.bpj.2009.05.062 19720014 PMC2749757

[4] Tong WC, Choi CY, Karche S, Holden AV, Zhang H, et al (2011) A computational model of the ionic currents, Ca^2+^ dynamics and action potentials underlying contraction of isolated uterine smooth muscle. PLoS ONE 6(4): e18685 10.1371/journal.pone.0018685 21559514 PMC3084699

[5] Andersen H, Barclay M (1995) A computer model of uterine contractions based on discrete contractile elements. Obstet Gynecol 86: 108–111. 10.1016/0029-7844(95)00111-4 7784002

[6] Vauge C, Mignot TM, Paris B, Breuiller-Fouché M, Chapron C, et al (2003) A mathematical model of the spontaneous contractions of the isolated uterine smooth muscle from patients receiving progestin treatment. Acta Biotheor 51: 19–34. 10.1023/A:1023048205232 12765250

[7] Young R (1997) A computer model of uterine contractions based on action potential propagation and intercellular calcium waves. Obstet Gynecol 89: 604–608. 10.1016/S0029-7844(96)00502-9 9083321

[8] Bursztyn L, Eytan O, Jaffa AJ, Elad D (2007) Modeling myometrial smooth muscle contraction. Reproductive Biomechanics 1101: 110–138. 10.1196/annals.1389.025 17303825

[9] Hai C, Murphy R (1988) Cross-bridge phosphorylation and regulation of latch state in smooth muscle. Am J Physiol Cell Physiol 254: C99–C106. 10.1152/ajpcell.1988.254.1.C99 3337223

[10] Rihana S, Terrien J, Germain G, Marque C (2009) Mathematical modelling of electrical activity of uterine muscle cells. Med Biol Eng Comput 47: 667–675. 10.1007/s11517-009-0433-4 19301052

[11] Garfield R (1994) Role of cell-to-cell coupling in control of myometrial contractility and labor in: garfield R. E. and tabb T. N. (eds.). Control of Uterine Contractility: 39–81. CRC Press.

[12] Taggart M, Blanks A, Kharche S, Holden A, Wang B, et al (2007) Towards understanding the myometrial physiome: approaches for the construction of a virtual physiological uterus. BMC Pregnancy Childbirth 7: S3 10.1186/1471-2393-7-S1-S3 17570163 PMC1892060

[13] Chan Y, van den Berg H, Moore J, Quenby S, Blanks A (2014) Assessment of myometrial transcriptome changes associated with spontaneous human labour by high-throughput RNA-seq. Exp Physiol 99(3): 510–524. 10.1113/expphysiol.2013.072868 24273302

[14] Klemic KG, Shieh CC, Kirsch GE, Jones SW (1998) Inactivation of Kv2.1 potassium channels. Biophys J 74: 1779–1789. 10.1016/S0006-3495(98)77888-9 9545040 PMC1299522

[15] Patel AJ, Lazdunski M, Honore E (1997) Kv2.1/Kv9.3, A novel ATP-dependent delayed-rectifier K^+^ channel in oxygen-sensitive pulmonary artery myocytes. EMBO J 16: 6615–6625. 10.1093/emboj/16.22.6615 9362476 PMC1170266

[16] Kramer JW, Post MA, Brown AM, Kirsch GE (1998) Modulation of potassium channel gating by coexpression of Kv2.1 with regulatory Kv5.1 or Kv6.1 alpha-subunits. Am J Physiol Cell Physiol 274: C1501. 10.1152/ajpcell.1998.274.6.C1501 9696692

[17] Bao L, Cox DH (2005) Gating and ionic currents reveal how the BK_Ca_ channel’s Ca^2+^sensitivity is enhanced by its *β*1 subunit. J Gen Physiol 126: 393–412. 10.1085/jgp.200509346 16186565 PMC2266624

[18] Lingle CJ, Zeng XH, Ding JP, Xia XM (2001) Inactivation of BK channels mediated by the Nh2 terminus of the *β*3b auxiliary subunit involves a two-step mechanism: Possible separation of binding and blockade. J Gen Physiol 117: 583–605. 10.1085/jgp.117.6.583 11382808 PMC2232400

[19] Horrigan FT, Aldrich RW (2002) Coupling between voltage sensor activation, Ca^2+^ binding and channel opening in large conductance (BK) potassium channels. J Gen Physiol 120: 267–305. 10.1085/jgp.20028605 12198087 PMC2229516

[20] Wang B, Rothberg BS, Brenner R (2006) Mechanism of beta4 subunit modulation of BK channels. J Gen Physiol 127: 449–465. 10.1085/jgp.200509436 16567466 PMC2151511

[21] Köhler M, Hirschberg B, Bond CT, Kinzie JM, Marrion NV, et al (1996) Small-conductance, calcium-activated potassium channels from mammalian brain. Science 273: 1709–1714. 10.1126/science.273.5282.1709 8781233

[22] Hirschberg B, Maylie J, Adelman JP, Marrion NV (1998) Gating of recombinant small-conductance Ca^2+^-activated K^+^ channels by calcium. J Gen Physiol 111: 565–581. 10.1085/jgp.111.4.565 9524139 PMC2217120

[23] Xia XM, Fakler B, Rivard A, Wayman G, Johnson-Pais T, et al (1998) Mechanism of calcium gating in small-conductance calcium-activated potassium channels. Nature 395: 503–507. 10.1038/26758 9774106

[24] Joiner WJ, Wang LY, Tang MD, Kaczmarek LK (1997) hSK4, a member of a novel subfamily of calcium-activated potassium channels. Proc Natl Acad Sci USA 94: 11013–11018. 10.1073/pnas.94.20.11013 9380751 PMC23566

[25] Wang SM, Liu SG, Morales MJ, Strauss HC, Rasmusson RL (1997) A quantitative analysis of the activation and inactivation kinetics of hERG expressed in *Xenopus* oocytes. J Physiol 502: 45–60. 10.1111/j.1469-7793.1997.045bl.x 9234196 PMC1159571

[26] Perez-Reyes E, Cribbs LL, Daud A, Lacerda AE, Barclay J, et al (1998) Molecular characterization of a neuronal low-voltage-activated T-type calcium channel. Nature 391: 896–900. 10.1038/36110 9495342

[27] Arreola J, Melvin JE, Begenisich T (1996) Activation of calcium-dependent chloride channels in rat parotid acinar cells. J Gen Physiol 108: 35–47. 10.1085/jgp.108.1.35 8817383 PMC2229297

[28] Rudy B, Sen K, Vega-Saenz De Miera E, Lau D, Ried T, et al (1991) Cloning of a human cDNA expressing a high voltage-activating, TEA-sensitive, type-A K^+^ channel which maps to chromosome-1 band-p21. J Neurosci Res 29: 401–412. 10.1002/jnr.490290316 1920536

[29] Jerng HH, Covarrubias M (1997) K^+^ channel inactivation mediated by the concerted action of the cytoplasmic N- and C-terminal domains. Biophys J 72: 163–174. 10.1016/S0006-3495(97)78655-7 8994601 PMC1184305

[30] Nakamura TY, Pountney DJ, Nandi S, Artman M, Rudy B, et al (2001) Different effects of the Ca^2+^-binding protein, KChIP1, on two Kv4 subfamily members, Kv4.1 and Kv4.2. J Mol Cell Cardiol 33: A83. 10.1016/s0014-5793(01)02560-1 11423117

[31] Beck EJ, Bowlby M, An WF, Rhodes KJ, Covarrubias M (2002) Remodelling inactivation gating of Kv4 channels by KChIP1, a small-molecular-weight calcium-binding protein. J Physiol 538: 691–706. 10.1113/jphysiol.2001.013127 11826158 PMC2290090

[32] Patel SP, Parai R, Campbell DL (2004) Regulation of Kv4.3 voltage-dependent gating kinetics by KChIP2 isoforms. J Physiol 557: 19–41. 10.1113/jphysiol.2003.058172 14724186 PMC1665034

[33] Pusch M, Magrassi R, Wollnik B, Conti F (1998) Activation and inactivation of homomeric KvLQT1 potassium channels. Biophys J 75: 785–792. 10.1016/S0006-3495(98)77568-X 9675180 PMC1299753

[34] Schrøder RL, Jespersen T, Christophersen P, Strøbæk D, Jensen BS, et al (2001) KCNQ4 channel activation by BMS-204352 and retigabine. Neuropharmacol 40: 888–898. 10.1016/S0028-3908(01)00029-6 11378159

[35] Doring F, Derst C, Wischmeyer E, Karschin C, Schneggenburger R, et al (1998) The epithelial inward rectifier channel Kir7.1 displays unusual K^+^ permeation properties. J Neurosci 18: 8625–8636. 9786970 10.1523/JNEUROSCI.18-21-08625.1998 PMC6793533

[36] Miyoshi H, Mary B, Lynette B (1996) Voltage-clamp studies of gap junctions between uterine muscle cells during term and preterm labor. Biophys J 71: 1324–1334. 10.1016/S0006-3495(96)79332-3 8874006 PMC1233599

[37] Faber GM, Silva J, Livshitz L, Rudy Y (2007) Kinetic properties of the cardiac L-type Ca^2+^ channel and its role in myocyte electrophysiology: A theoretical investigation. Biophys J 92: 1522–1543. 10.1529/biophysj.106.088807 17158566 PMC1796810

[38] Toulme E, Garcia A, Samways D, Egan T (2010) P2X4 receptors in activated C8-B4 cells of cerebellar microglial origin. J Gen Physiol 135: 333–353. 10.1085/jgp.200910336 20231374 PMC2847917

[39] McCloskey C, Rada C, Bailey E, McCavera S, van den Berg HA, et al (2014) The inwardly rectifying K^+^ channel KIR7.1 controls uterine excitability throughout pregnancy. EMBO Mol Med 6(9): 1161–1174. 10.15252/emmm.201403944 25056913 PMC4197863

[40] Aaronson PI, Sarwar U, Gin S, Rockenbauch U, Connolly M, et al (2006) A role for voltage-gated, but not Ca^2+^-activated, K^+^ channels in regulating spontaneous contractile activity in myometrium from virgin and pregnant rats. Br J Pharmacol 147: 815–824. 10.1038/sj.bjp.0706644 16415906 PMC1751504

[41] Greenwood IA, Yeung SY, Tribe RM, Ohya S (2009) Loss of functional K^+^ channels encoded by *ether-à-go-go*-related genes in mouse myometrium prior to labour onset. J Physiol 587: 2313–2326. 10.1113/jphysiol.2009.171272 19332483 PMC2697300

[42] Pierce SL, Kresowik V, Lamping K, England S (2008) Overexpression of SK3 channels dampens uterine contractility to prevent preterm labor in mice. Biol Reprod 78: 1058–1063. 10.1095/biolreprod.107.066423 18305226 PMC2930016

[43] Wray S (1993) Uterine contraction and physiological-mechanisms of modulation. Am J Physiol 264: C1–C18. 8430759 10.1152/ajpcell.1993.264.1.C1

[44] Gomes P, Srinivas S, Van Driessche W, Vereecke J, Himpens B (2005) ATP release through connexion hemichannels in corneal endothelial cells. Invest Ophthalmol Vis Sci 46: 1208–1218. 10.1167/iovs.04-1181 15790881

[45] Lu Z (2004) Mechanism of rectification in inward-rectifier K^+^ channels. Ann Rev of Physiol 66: 103–129. 10.1146/annurev.physiol.66.032102.150822 14977398

[46] Zhao H, Ning Y, Fleming C (2005) Gap junctional hemichannel-mediated ATP release an hearing controls in the inner ear. PNAS 102: 18724–18729. 10.1073/pnas.0506481102 16344488 PMC1317927

[47] Urabe S, Miyoshi H, Fujiwara H, Yamaoka K, Kudo Y (2009) Enhanced expression of P2X4 and P2X7 purinergic receptors in the myometrium of pregnant rats in preterm delivery models. Reprod Sci: 1186–1192. 10.1177/1933719109344630 19767540

[48] Miyoshi H, Yamaoka K, Urabe S, Kudo Y (2010) Functional expression of purinergic P2X7 receptors in pregnant rat myometrium. Am J Physiol 298(4): R1117–R1124. 10.1152/ajpregu.00507.2009 20071613

[49] Miyoshi H, Yamaoka K, Urabe S, Kudo Y (2012) ATP-induced currents carried through P2X7 receptor in rat myometrial cells. Reprod Sci 19(12): 1285–1291. 10.1177/1933719112450333 22814097

[50] Stojikovic S, Zonghe Y, Obsil T, Zemkova H (2010) Structural insights into the function of P2X4: and ATP-gated ratio channel of neuroendocrine cells. Cell Mol Neurobiol 30: 1251–1258. 10.1007/s10571-010-9568-y 21107680 PMC3042234

[51] Soto F, Garcia-Guzman M, Gomez-Hernandez J, Hollmann M, Karschin C (1996) P2X4: An ATP-activated ionotropic receptor cloned from rat brain. Neurobiology 93: 3684–3688. 10.1073/pnas.93.8.3684 PMC39672 8622997

[52] Lewis C (1979) Ion-concentration dependence of the reversal potential and the single-channel conductance of ion channels at the frog neuromuscular junction. J Physiol 286: 417–445. 10.1113/jphysiol.1979.sp012629 312319 PMC1281581

[53] Spangler S (1972) Expansion of the constant field equation to include both divalent and monovalent ions. Ala J Med Sci 9: 218–223. 5045041

[54] Hille B (2008) PIP_2_ is a necessary cofactor for ion channel function: How and why. Annu Rev Biophys 37: 175–195. 10.1146/annurev.biophys.37.032807.125859 18573078 PMC2692585

[55] Rodriguez N, Amarouch M (2010) Phosphatidylinositol-4,5-bisphosphate (PIP_2_) stabilizes the open pore conformation of the Kv11.1 (hERG) channel. Biophys J 99: 1110–1118. 10.1016/j.bpj.2010.06.013 20712994 PMC2920645

[56] Pattnaik B, Hughes B (2009) Regulation of Kir channels in bovine retinal pigment epithelial cells by phosphatidylinositol 4,5-bisphosphate. Am J Physiol Cell Physiol 297: C1001–C1011. 10.1152/ajpcell.00250.2009 19641096 PMC2770741

[57] Loussouarn K, Park K, Bellocq C, Baro I, Charpentier F, et al (2003) Phosphatidylinositol-4,5-bisphosphate, PIP_2_, controls KCNQ1/KCNE1 voltage-gated potassium channels: a functional homology between voltage-gated and inward rectifier K^+^ channels. EMBO J 22: 5412–5421. 10.1093/emboj/cdg526 14532114 PMC213780

[58] Jaggar J, Porter V, Lederer W, Nelson M (2000) Calcium sparks in smooth muscle. Am J Physiol Cell Physiol 278: C235–C256. 10666018 10.1152/ajpcell.2000.278.2.C235

[59] Wray S, Burdyga T, Karen N (2005) Calcium signalling in smooth muscle. Cell Calcium 38: 397–407. 10.1016/j.ceca.2005.06.018 16137762

[60] Bernstein K, Vink J, Wen Fu X, Wakita H, Danielsson J, et al (2014) Calcium-activated chloride channels anoctamin 1 and 2 promote murine uterine smooth muscle contractility. Am J Obstet Gynecol 211: 1-e1–1-e10. 10.1016/j.ajog.2014.06.018 24928056 PMC4253652

[61] Cheng H, Lederer W, Cannell M (1993) Calcium sparks: elementary events underlying excitation-contraction coupling in heart muscle. Science 262: 740–744. 10.1126/science.8235594 8235594

[62] Klein M, Cheng L, Santana Y, Jiang W, Lederer J, et al (1996) Two mechanisms of quantised calcium release in skeletal muscle. Nature 379: 455–458. 10.1038/379455a0 8559251

[63] Tsugorka A, Rios E, Blatter L (1995) Imaging elementary events of calcium release in skeletal muscle cells. Science 269: 1723–1726. 10.1126/science.7569901 7569901

[64] Nelson M, Cheng H, Rubart M, Santana L, Bonev A, et al (1995) Relaxation of arterial smooth muscle by calcium sparks. Science 270: 633–637. 10.1126/science.270.5236.633 7570021

[65] Wellman G, Nelson M (2003) Signalling between SR and plasmalemma in smooth muscle: sparks and the activation of Ca^2+^-sensitive ion channels. Cell Calcium 34: 211–229. 10.1016/S0143-4160(03)00124-6 12887969

[66] Porter V, Bonev A, Knot H, Heppner T, Stevenson A, et al (1998) Frequency modulation of Ca^2+^ sparks is involved in regulation of arterial diameter by cyclic nucleotides. Cell Physiol 274(5): C1346–C1355. 10.1152/ajpcell.1998.274.5.C1346 9612222

[67] Emmert-Buck M, Bonner R, Smith P, Chuaqui R, Zhuang Z, et al (1996) Laser capture microdissection. Science 274: 998–1001. 10.1126/science.274.5289.998 8875945

[68] Fink M, Noble D (2009) Markov models for ion channels: Versatility versus identifiability and speed. Phys Trans R Soc A 367: 2161–2179. 10.1098/rsta.2008.0301 19414451

[69] Huys Q, Ahrens M, Paninski L (2006) Efficient estimation of detailed single-neuron models. J Neurophysiol 96: 872–890. 10.1152/jn.00079.2006 16624998

[70] van den Berg HA (2011) Mathematical Models of Biological Systems. Oxford University Press.

[71] Saltelli A, Annoni P (2010) How to avoid a perfunctory sensitivity analysis. Environ Modell Software 25: 1508–1517. 10.1016/j.envsoft.2010.04.012

[72] Sher AA, Wang K, Wathen A, Maybank PJ, Mirams GR, et al (2013) A local sensitivity analysis method for developing biological models with identifiable parameters: Application to cardiac ionic channel modelling. Future Gener Comp Sy 29: 591–598. 10.1016/j.future.2011.09.006

[73] Sobie EA (2009) Parameter sensitivity analysis in electrophysiological models using multivariate regression. Biophys J 96: 1264–1274. 10.1016/j.bpj.2008.10.056 19217846 PMC2717232

[74] Khan RN, Matharoo-Ball B, Arulkumaran S, Ashford MLJ (2001) Potassium channels in the human myometrium. Exp Physiol 86: 255–264. 10.1113/eph8602181 11429642

[75] Wang S, Yoshino M, Sui J, Wakui M, Kao P, et al (1998) Potassium current in freshly dissociated uterine myocytes from nonpregnant and late-pregnant rats. J Gen Physiol 112: 737–756. 10.1085/jgp.112.6.737 9834143 PMC2229446

[76] Matharoo-Ball B, Ashford M, Arulkumaran S, Khan R (2003) Down-regulation of the *α*- and *β*- subunits of the calcium-activated potassium channel in human myometrium with parturition. Biol Reprod 68: 2135–2141. 10.1095/biolreprod.102.010454 12606455

[77] Tseng-Crank J, Foster CD, Krause JD, Mertz R, Godinot N, et al (1994) Cloning, expression, and distribution of functionally distinct Ca^2+^-activated K^+^ channel isoforms from human brain. Neuron 13: 1315–1330. 10.1016/0896-6273(94)90418-9 7993625

[78] Rae J, Cooper K, Gates P, Watsky M (1991) Low access resistance perforated patch recordings using amphotericin B. J Neurosc Methods 37: 15–26. 10.1016/0165-0270(91)90017-T 2072734

[79] Frech GC, Vandongen AMJ, Schuster G, Brown AM, Joho RH (1989) A novel potassium channel with delayed rectifier properties isolated from rat-brain by expression cloning. Nature 340: 642–645. 10.1038/340642a0 2770868

[80] Knock GA, Smirnov SV, Aaronson PI (1999) Voltage-gated K^+^ currents in freshly isolated myocytes of the pregnant human myometrium. J Physiol 518: 769–781. 10.1111/j.1469-7793.1999.0769p.x 10420013 PMC2269461

[81] Klemic KG, Shieh CC, Kirsch GE, Jones SW (1997) Gating kinetics of the rat Kv2.1 potassium channel expressed in *Xenopus* oocytes. Biophys J 72: MPOS8.

[82] Aldrich RW (1981) Inactivation of voltage-gated delayed potassium current in molluscan neurons—a kinetic model. Biophys J 36: 519–532. 10.1016/S0006-3495(81)84750-9 6275919 PMC1327644

[83] Kerschensteiner D, Monje F, Stocker M (2003) Structural determinants of the regulation of the voltage-gated potassium channel Kv2.1 by the modulatory alpha-subunit Kv9.3. J Biol Chem 278: 18154–18161. 10.1074/jbc.M213117200 12642579

[84] Post MA, Kirsch GE, Brown AM (1996) Kv2.1 and electrically silent Kv6.1 potassium channel subunits combine and express a novel current. FEBS Lett 399: 177–182. 10.1016/S0014-5793(96)01316-6 8980147

[85] Bonifazzi C, Belluzzi O, Sacchi O (1988) Kinetic analysis of incomplete current tracings according to the Hodgkin-Huxley model. J Theor Biol 130: 183–190. 10.1016/S0022-5193(88)80093-6 2458509

[86] Wang SM, Bondarenko VE, Qu YJ, Bett GCL, Morales MJ, et al (2005) Time- and voltage-dependent components of Kv4.3 inactivation. Biophys J 89: 3026–3041. 10.1529/biophysj.105.059378 16100281 PMC1366800

[87] Lundby A, Olesen S (2006) KCNE3 is an inhibitory subunit of the kv4.3 potassium channel. Biochem Biophys Res Commun 346: 958–967. 10.1016/j.bbrc.2006.06.004 16782062

[88] Sanguinetti MC, Jiang CG, Curran ME, Keating MT (1995) A mechanistic link between an inherited and an acquired cardiac-arrhythmia—hERG encodes the *I*_*KR*_ potassium channel. Cell 81: 299–307. 10.1016/0092-8674(95)90340-2 7736582

[89] Sanguinetti MC, Tristani-Firouzi M (2006) hERG potassium channels and cardiac arrhythmia. Nature 440: 463–469. 10.1038/nature04710 16554806

[90] Barhanin J, Lesage F, Guillemare E, Fink M, Lazdunski M, et al (1996) KvLQT1 and IsK (minK) proteins associate to form the *I*_Ks_ cardiac potassium current. Nature 384: 78–80. 10.1038/384078a0 8900282

[91] Sanguinetti MC, Curran ME, Zou A, Shen J, Spector PS, et al (1996) Coassembly of KvLQT1 and mink (IsK) proteins to form cardiac I_*Ks*_ potassium channel. Nature 384: 80–83. 10.1038/384080a0 8900283

[92] McCallum L, Pierce S, England S, Greenwood I, Tribe R (2010) The contribution of Kv7 channels to pregnant mouse and human myometrial contractility. J Cell Mol Med 15(3): 577–586. 10.1111/j.1582-4934.2010.01021.x PMC3922379 20132415

[93] Schrœder BC, Waldegger S, Fehr S, Bleich M, Warth R, et al (2000) A constitutively open potassium channel formed by KCNQ1 and KCNE3. Nature 403: 196–199. 10.1038/35003200 10646604

[94] Grunnet M, Jespersen T, Rasmussen HB, Ljungstrom T, Jorgensen NK, et al (2002) KCNE4 is an inhibitory subunit to the KCNQ1 channel. J Physiol 542: 119–130. 10.1113/jphysiol.2002.017301 12096056 PMC2290389

[95] Manderfield LJ, Daniels MA, Vanoye CG, George AL (2009) KCNE4 domains required for inhibition of KCNQ1. J Physiol 587: 303–314. 10.1113/jphysiol.2008.161281 19029186 PMC2670046

[96] Miceli F, Cilio MR, Taglialatela M, Bezanilla F (2009) Gating currents from neuronal Kv7.4 channels general features and correlation with the ionic conductance. Channels 3: 274–283. 10.4161/chan.3.4.9477 19690464

[97] Atkinson NS, Robertson GA, Ganetzky B (1991) A component of calcium- activated potassium channels encoded encoded by the *Drosophila*-Slo locus. Science 253: 551–555. 10.1126/science.1857984 1857984

[98] Butler A, Tsunoda S, McCobb DP, Wei A, Salkoff L (1993) mSlo, a complex mouse gene encoding mouse gene encoding Maxi calcium-activated potassium channels. Science 261: 221–224. 10.1126/science.7687074 7687074

[99] Pallanck L, Ganetzky B (1994) Cloning and characterization of human and mouse homologs of the drosophila calcium-activated potassium channel gene, slowpoke. Hum Mol Genet 3: 1239–1243. 10.1093/hmg/3.8.1239 7987297

[100] Cox DH, Aldrich RW (2000) Role of the beta1 subunit in large-conductance Ca^2+^-activated K^+^ channel gating energetics—mechanisms of enhanced Ca^2+^ sensitivity. J Gen Physiol 116: 411–432. 10.1085/jgp.116.3.411 10962017 PMC2233685

[101] Nimigean CM, Magleby KL (1999) *β*-subunits increase the calcium sensitivity of mSlo by stabilizing bursting kinetics. Biophys J 76: A328.

[102] Nimigean CM, Magleby KL (2000) Functional coupling of the *β*1 subunit to the large conductance Ca^2+^-activated K^+^ channel in the absence of Ca^2+^ increased Ca^2+^ sensitivity from a Ca^2+^-independent mechanism. J Gen Physiol 115: 719–734. 10.1085/jgp.115.6.719 10828246 PMC2232893

[103] Xia XM, Ding JP, Lingle CJ (1999) Molecular basis for the inactivation of Ca^2+^- and voltage-dependent BK channels in adrenal chromaffin cells and rat insulinoma tumor cells. J Neurosci 19: 5255–5264. 10377337 10.1523/JNEUROSCI.19-13-05255.1999 PMC6782330

[104] Weiger TM, Holmqvist MH, Levitan IB, Clark FT, Sprague S, et al (2000) A novel nervous system beta subunit that downregulates human large conductance calcium-dependent potassium channels. J Neurosci 20: 3563–3570. 10804197 10.1523/JNEUROSCI.20-10-03563.2000 PMC6772688

[105] Wang LJ, Sobie EA (2008) Mathematical model of the neonatal mouse ventricular action potential. Am J Physiol Heart Circ Physiol 294: H2565–H2575. 10.1152/ajpheart.01376.2007 18408122 PMC3032983

[106] Brown A, Adelman J, Bond C, Korniyenko I, Taylor M (2006) Modulation of uterine contractility by small-conductance Ca^2+^-activated K^+^ SK3 channels. FASEB J 20: A1242.

[107] Barfod ET, Moore AL, Lidofsky SD (2001) Cloning and functional expression of a liver isoform of the small conductance Ca^2+^-activated K^+^ channel SK3. Am J Physiol Cell Physiol 280: C836–C842. 11245600 10.1152/ajpcell.2001.280.4.C836

[108] Khanna R, Chang M, Joiner W, Kaczmarek L, Schlichter L (1999) hSK4/hIK1, a Calmodulin-binding K_Ca_ channel in human T lymphocytes. J Biol Chem 274 10.1074/jbc.274.21.14838 10329683

[109] Krapivinsky G, Medina I, Eng L, Krapivinsky L, Yang YH, et al (1998) A novel inward rectifier K^+^ channel with unique pore properties. Neuron 20: 995–1005. 10.1016/S0896-6273(00)80480-8 9620703

[110] Shmigol AV, Eisner DA, Wray S (1998) Properties of voltage-activated ${\text{Ca}}_{i}^{2+}$ transients in single smooth muscle cells isolated from pregnant rat uterus. J Physiol 511: 803–811. 10.1111/j.1469-7793.1998.803bg.x 9714861 PMC2231157

[111] Sun H, Leblanc N, Nattel S (1997) Mechanisms of inactivation of L-type calcium channels in human atrial myocytes. Am J Physiol Heart Circ Physiol 272: H1625–H1635. 10.1152/ajpheart.1997.272.4.H1625 9139944

[112] Blanks AM, Zhao ZH, Shmygol A, Bru-Mercier G, Astle S, et al (2007) Characterization of the molecular and electrophysiological properties of the T-type calcium channel in human myometrium. J Physiol 581: 915–926. 10.1113/jphysiol.2007.132126 17446221 PMC1976399

[113] Hu H, Marban E (1998) Isoform-specific inhibition of L-type calcium channels by dihydropyridines is independent of isoform-specific gating properties. Molecular Pharmacology 53: 902–907. 9584217

[114] Shmigol AV, Eisner DA, Wray B (2001) Simultaneous measurements of changes in sarcoplasmic reticulum and cytosolic Ca^2+^ in rat uterine smooth muscle cells. J Physiol 531: 707–713. 10.1111/j.1469-7793.2001.0707h.x 11251052 PMC2278495

[115] Luo CH, Rudy Y (1994) A dynamic model of the cardiac ventricular action-potential. I. Simulations of ionic currents and concentration changes. Circ Res 74: 1071–1096. 10.1161/01.RES.74.6.1097 7514509

[116] Varghese A, Sell G (1997) A conservation principle and its effect on the formulation of Na-Ca exchanger current in cardiac cells. J Theoret Biol 189: 33–40. 10.1006/jtbi.1997.0487 9398501

[117] Jones K, Shmygol A, Kupittayanant S, Wray S (2004) Electrophysiological characterization and functional importance of calcium-activated chloride channel in rat uterine myocytes. Pflügers Arch 448: 36–43. 14740218 10.1007/s00424-003-1224-7

[118] Lee JH, Daud AN, Cribbs LL, Lacerda AE, Pereverzev A, et al (1999) Cloning and expression of a novel member of the low voltage-activated T-type calcium channel family. J Neurosci 19: 1912–1921. 10066244 10.1523/JNEUROSCI.19-06-01912.1999 PMC6782566

[119] Bennett M (1994) Connexins in disease. Nature 368: 18–19. 10.1038/368018a0 8107878

[120] Elfgang C, Eckert H, Lichtenberg-Frate A, Butterweck O, Klein R (1995) Specific permeability and selective formation of gap junction channels in connexin-trasfected HeLa cells. J Cell Biol 129: 805–817. 10.1083/jcb.129.3.805 7537274 PMC2120441

[121] Goodenough D, Godiger D, Paul D (1996) Connexins, connexons, and intercellular communication. Annu Rev Biochem 65: 475–502. 10.1146/annurev.bi.65.070196.002355 8811187

[122] Paul D (1986) Molecular cloning of cDNA for rat liver gap junction protein. J Cell Biol 103: 123–134. 10.1083/jcb.103.1.123 3013898 PMC2113807

[123] Chow L, Lye S (1994) Expression of the gap junction connexin-43 is increased in the human myometrium toward term and with onset of labor. Am J Obstet Gynecol 170: 788–795. 10.1016/S0002-9378(94)70284-5 8141203

[124] Tabb T, Thilander G, Grover A (1992) An immunohistochemical and immunocytologic study of increase in myometrial gap junctions (and connexin43) in rats and human during pregnancy. Am J Obstet Gynecol 167: 559–567. 10.1016/S0002-9378(11)91453-7 1323215

[125] Garfield R, Sims S, Kannan M, Daniel E (1978) Possible role of gap junctions in activation of myometrium during parturition. Am J Physiol 235: C168–C179. 727239 10.1152/ajpcell.1978.235.5.C168

[126] Saffitz J, Davis B, Darrow U (1995) The molecular basis of anisotropy: role of gap junctions. Electrophysiol 6: 498–510. 10.1111/j.1540-8167.1995.tb00423.x 7551319

[127] Enyedi P, Czirjak G (2010) Molecular background of leak K^+^ currents: two-pore domain potassium channels. Physiol Rev 90: 559–605. 10.1152/physrev.00029.2009 20393194

[128] Janis R, Crankshaw D, Daniel E (1977) Control of intracellular Ca^2+^ activity in rat myometrium. Am J Physiol 232: C50–C58. 189618 10.1152/ajpcell.1977.232.1.C50

[129] Enyedi P, Czirjak G (2010) Molecular background of leak K^+^ currents: Two-pore domain potassium channels. Physiol Rev 90: 559–605. 10.1152/physrev.00029.2009 20393194

[130] Grover A, Kwan C, Daniels E (1982) Ca^2+^-dependence of calcium uptake by rat myometrium plasma membrane. Am J Physiol 242: C278–C282. 7081424 10.1152/ajpcell.1982.242.5.C278

[131] Carrera F, Proverbio T, Marin R (2000) Ca-ATPase of human myometrium plasma membranes. Physiol Res 49: 331–338. 11043920

[132] Guerini D (1998) The significance of the isoforms of plasma membrane calcium ATPase. Cell Tissue Res 292: 191–197. 10.1007/s004410051050 9560462

[133] Popescu L, Ignat P (1983) Calmodulin-dependent Ca^2+^-pump ATPase of human smooth muscle sarcolemma. Cell Calcium 4: 219–235. 10.1016/0143-4160(83)90001-5 6315240

[134] Taylor F, Paton D (1970) Characteristics of electrogenic sodium pumping in rat myometrium. J Gen Physiol 56: 360–375. 10.1085/jgp.56.3.360 4920320 PMC2225959

[135] Parkington H, Tonta M (1999) Contractile activity, membrane potential, and cytoplasmic calcium in human uterine smooth muscle in the their trimester of pregnancy and during labor. Am J Obstet Gynecol 181: 1445–1451. 10.1016/S0002-9378(99)70390-X 10601927

[136] Shmigol A, Eisner D, Wray S (1998) Carboxyeosin decreases the rate of decay of the [Ca^2+^]_*i*_ transient in uterine smooth muscle cells isolated from pregnant rats. Pflügers Arch 437: 158–160. 9817801 10.1007/s004240050761

[137] Matthew A, Shmygol A, Wray S (2004) Ca^2+^ entry, efflux and release in smooth muscle. Biol Res 37: 617–624. 10.4067/S0716-97602004000400017 15709690

