# Supplementary material for: Reconstruction of Cell Surface Densities of Ion Pumps, Exchangers, and Channels from mRNA Expression, Conductance Kinetics, Whole-Cell Calcium, and Current-Clamp Voltage Recordings, with an Application to Human Uterine Smooth Muscle Cells
Source: PLoS Comput Biol. 2016 Apr 22;12(4):e1004828. doi: 10.1371/journal.pcbi.1004828 (PMC4841602; doi:10.1371/journal.pcbi.1004828)
Supplement: S1 Equations — (PDF) [file pcbi.1004828.s003.pdf]

Supplementary Mathematical

Reversal potentials:

$$
\begin{aligned}
E_{\mathrm{K}} &= \frac{RT}{F} \ln \frac{[\mathrm{K}^+]_o}{[\mathrm{K}^+]_i} \\
E_{\mathrm{Ca}} &= \frac{RT}{2F} \ln \frac{[\mathrm{Ca}^{2+}]_o}{[\mathrm{Ca}^{2+}]_i} \\
E_{\mathrm{Cl}} &= \frac{RT}{F} \ln \frac{[\mathrm{Cl}^-]_i}{[\mathrm{Cl}^-]_o}
\end{aligned}
$$

Kv2.1

$$I_{Kv2.1} = \kappa_{Kv2.1} G_{Kv2.1} O^{[Kv2.1]} (V - E_{\mathrm{K}})$$

$$
\begin{aligned}
k_v^{[\mathrm{Kv2.1}]} &= 12 \ \exp\left[0.77 \frac{VF}{RT}\right] \\
k_{-v}^{[\mathrm{Kv2.1}]} &= 42 \ \exp\left[-0.54 \frac{VF}{RT}\right] \\
k_{-o}^{[\mathrm{Kv2.1}]} &= 31 \ \exp\left[-0.5 \frac{VF}{RT}\right]
\end{aligned}
$$

$$
\begin{aligned}
\frac{dC_0^{[\mathrm{Kv2.1}]}}{dt} &= 1-(O^{[\mathrm{Kv2.1}]}+\sum_{i=1}^{4}C_i^{[\mathrm{Kv2.1}]}+\sum_{i=0}^{5}I_i^{[\mathrm{Kv2.1}]}) \\
\frac{dC_1^{[\mathrm{Kv2.1}]}}{dt} &= 4k_v^{[\mathrm{Kv2.1}]}C_0^{[\mathrm{Kv2.1}]}-(k_{-v}^{[\mathrm{Kv2.1}]}+3k_v^{[\mathrm{Kv2.1}]}+k_1^{[\mathrm{Kv2.1}]}f^3)C_1^{[\mathrm{Kv2.1}]} \\
&\quad +2k_{-v}^{[\mathrm{Kv2.1}]}C_2^{[\mathrm{Kv2.1}]}+\frac{k_{-1}^{[\mathrm{Kv2.1}]}}{f^3}I_1^{[\mathrm{Kv2.1}]} \\
\frac{dC_2^{[\mathrm{Kv2.1}]}}{dt} &= 3k_v^{[\mathrm{Kv2.1}]}C_1^{[\mathrm{Kv2.1}]}-(2k_{-v}^{[\mathrm{Kv2.1}]}+2k_v^{[\mathrm{Kv2.1}]}+k_1^{[\mathrm{Kv2.1}]}f^2)C_2^{[\mathrm{Kv2.1}]} \\
&\quad +3k_{-v}^{[\mathrm{Kv2.1}]}C_3^{[\mathrm{Kv2.1}]}+\frac{k_{-1}^{[\mathrm{Kv2.1}]}}{f^2}I_2^{[\mathrm{Kv2.1}]} \\
\frac{dC_3^{[\mathrm{Kv2.1}]}}{dt} &= 2k_v^{[\mathrm{Kv2.1}]}C_2^{[\mathrm{Kv2.1}]}-(3k_{-v}^{[\mathrm{Kv2.1}]}+k_v^{[\mathrm{Kv2.1}]}+k_1^{[\mathrm{Kv2.1}]}f)C_3^{[\mathrm{Kv2.1}]} \\
&\quad +4k_{-v}^{[\mathrm{Kv2.1}]}C_4^{[\mathrm{Kv2.1}]}+\frac{k_{-1}^{[\mathrm{Kv2.1}]}}{f}I_3^{[\mathrm{Kv2.1}]} \\
\frac{dC_4^{[\mathrm{Kv2.1}]}}{dt} &= k_v^{[\mathrm{Kv2.1}]}C_3^{[\mathrm{Kv2.1}]}-(4k_{-v}^{[\mathrm{Kv2.1}]}+k_o^{[\mathrm{Kv2.1}]}+k_1^{[Kv21]})C_4^{[\mathrm{Kv2.1}]} \\
&\quad +k_{-o}^{[\mathrm{Kv2.1}]}O_4^{[\mathrm{Kv2.1}]}+k_{-1}^{[Kv21]}I_4^{[Kv21]} \\
\frac{dI_0^{[\mathrm{Kv2.1}]}}{dt} &= k_1^{[\mathrm{Kv2.1}]}f^4C_0^{[\mathrm{Kv2.1}]}-(\frac{k_{-1}^{[\mathrm{Kv2.1}]}}{f^4}+4\frac{k_v^{[Kv21]}}{f})I_0^{[\mathrm{Kv2.1}]} \\
&\quad +k_{-v}^{[\mathrm{Kv2.1}]}fI_1^{[\mathrm{Kv2.1}]} \\
\frac{dI_1^{[\mathrm{Kv2.1}]}}{dt} &= 4\frac{k_v^{[\mathrm{Kv2.1}]}}{f}I_0^{[\mathrm{Kv2.1}]}+k_1^{[\mathrm{Kv2.1}]}f^3C_1^{[\mathrm{Kv2.1}]}-(fk_{-v}^{[\mathrm{Kv2.1}]}+\frac{k_{-1}^{[\mathrm{Kv2.1}]}}{f^3} \\
&\quad +3\frac{k_v^{[\mathrm{Kv2.1}]}}{f})I_1^{[\mathrm{Kv2.1}]}+2k_{-v}^{[\mathrm{Kv2.1}]}fI_2^{[Kv21]} \\
\frac{dI_2^{[\mathrm{Kv2.1}]}}{dt} &= 3\frac{k_v^{[\mathrm{Kv2.1}]}}{f}I_1^{[\mathrm{Kv2.1}]}+k_1^{[\mathrm{Kv2.1}]}f^2C_2^{[\mathrm{Kv2.1}]}-(2fk_{-v}^{[\mathrm{Kv2.1}]}+\frac{k_{-1}^{[\mathrm{Kv2.1}]}}{f^2} \\
&\quad +2\frac{k_v^{[\mathrm{Kv2.1}]}}{f})I_2^{[\mathrm{Kv2.1}]}+3k_{-v}^{[\mathrm{Kv2.1}]}fI_3^{[Kv21]} \\
\frac{dI_3^{[\mathrm{Kv2.1}]}}{dt} &= 2\frac{k_v^{[\mathrm{Kv2.1}]}}{f}I_2^{[\mathrm{Kv2.1}]}+k_1^{[\mathrm{Kv2.1}]}fC_3^{[\mathrm{Kv2.1}]}-(3fk_{-v}^{[\mathrm{Kv2.1}]}+\frac{k_{-1}^{[\mathrm{Kv2.1}]}}{f} \\
&\quad +\frac{k_v^{[\mathrm{Kv2.1}]}}{f})I_3^{[\mathrm{Kv2.1}]}+4k_{-v}^{[\mathrm{Kv2.1}]}fI_4^{[Kv21]} \\
\frac{dI_4^{[\mathrm{Kv2.1}]}}{dt} &= \frac{k_v^{[\mathrm{Kv2.1}]}}{f}I_3^{[\mathrm{Kv2.1}]}+k_1^{[\mathrm{Kv2.1}]}C_4^{[\mathrm{Kv2.1}]}-(4fk_{-v}^{[\mathrm{Kv2.1}]}+k_{-1}^{[\mathrm{Kv2.1}]}+ \\
&\quad k_o^{[\mathrm{Kv2.1}]}g)I_4^{[\mathrm{Kv2.1}]}+\frac{k_{-o}^{[\mathrm{Kv2.1}]}}{g}I_5^{[\mathrm{Kv2.1}]} \\
\frac{dI_5^{[\mathrm{Kv2.1}]}}{dt} &= k_o^{[\mathrm{Kv2.1}]}gI_4^{[\mathrm{Kv2.1}]}-(\frac{(k_{-o}^{[\mathrm{Kv2.1}]}+k_{-1}^{[\mathrm{Kv2.1}]})}{g})I_5^{[\mathrm{Kv2.1}]}+k_1^{[\mathrm{Kv2.1}]}gO^{[\mathrm{Kv2.1}]} \\
\frac{dO^{[\mathrm{Kv2.1}]}}{dt} &= k_o^{[\mathrm{Kv2.1}]}C_4^{[\mathrm{Kv2.1}]}-(k_{-o}^{[\mathrm{Kv2.1}]}+k_1^{[\mathrm{Kv2.1}]}g)O^{[\mathrm{Kv2.1}]}+\frac{k_{-1}^{[\mathrm{Kv2.1}]}}{g}I_5^{[\mathrm{Kv2.1}]}
\end{aligned}
$$

Kv2.1/Kv9.3

$$I_{\text{Kv9.3}} = \kappa_{\text{Kv9.3}}\, G_{\text{Kv9.3}}\, g_1\, (0.7 g_{2_{\text{fast}}} + 0.3 g_{2_{\text{slow}}})\, (V - E_{\text{K}})$$

$$\begin{aligned}
g_{1_\infty} &= \frac{1}{1 + \exp\left[\frac{3.2 - V}{21.8}\right]} \\
g_{2_\infty} &= \frac{1}{1 + \exp\left[\frac{44.9 + V}{10.4}\right]} \\
\tau_{g_1} &= \frac{1}{\exp\left[\frac{-80.3 - V}{10}\right] + \exp\left[\frac{-137.5 + V}{55}\right]}
\end{aligned}$$

Kv2.1/Kv6.1

$$I_{\text{Kv6.1}} = \kappa_{\text{Kv6.1}} G_{\text{Kv6.1}} l_1\, l_2 (V - E_{\text{K}})$$

$$\begin{aligned}
l_{1_\infty} &= \frac{1}{1 + \exp\left[\frac{-9.4 - V}{11.8}\right]} \\
l_{2_\infty} &= \frac{1}{1 + \exp\left[\frac{65.9 + V}{6.4}\right]} \\
\tau_{l_1} &= \frac{1}{\exp\left[\frac{-18.2 - V}{2}\right] + \exp\left[\frac{-107 + V}{30}\right]}
\end{aligned}$$

Kv3.4

$$I_{\text{Kv3.4}} = \kappa_{\text{Kv3.4}}\, G_{\text{Kv3.4}}\, a_1\, a_2\, (V - E_{\text{K}})$$

$$\begin{aligned}
a_{1\infty} &= \frac{1}{1 + \exp\left[\frac{19.1 - V}{11.3}\right]} \\
a_{2\infty} &= \frac{1}{1 + \exp\left[\frac{15 + V}{7.4}\right]} \\
\tau_{a_1} &= 40 - \frac{37.7}{1 + \exp\left[-V/28\right]} \\
\tau_{a_2} &= 12 + 165.6\ \exp\left[-V/11.2\right]
\end{aligned}$$

Kv4.1

$$I_{\text{Kv4.1}} = \kappa_{\text{Kv4.1}}\, G_{\text{Kv4.1}}\, {b_1}^4\, (0.18\, b_{2_{\text{fast}}} + 0.42\, b_{2_{\text{inter}}} + 0.4\, b_{2_{\text{slow}}})\, (V - E_{\text{K}})$$

$$
\begin{aligned}
b_{1\infty} &= \frac{1}{1+\exp\left[\frac{-49-V}{22.3}\right]} \\
b_{2\infty} &= \frac{1}{1+\exp\left[\frac{69+V}{5}\right]} \\
\tau_{b_1} &= 1.96 + \frac{5.5}{1+\exp\left[V/12.5\right]} \\
\tau_{b_{2_{\text{fast}}}} &= 16 + 11.4\exp\left[-V/15.9\right] \\
\tau_{b_{2_{\text{inter}}}} &= 73.6 + 18\exp\left[-V/19\right] \\
\tau_{b_{2_{\text{slow}}}} &= 252.2 + 0.74V
\end{aligned}
$$

Kv4.3

$$
I_{\text{Kv4.3}} = \kappa_{\text{Kv4.3}}\, G_{\text{Kv4.3}}\, O^{[\text{Kv4.3}]}\,(V - E_{\text{K}})
$$

$$
\begin{aligned}
\alpha^{[\text{Kv4.3}]} &= 12\exp\left[0.77\frac{VF}{RT}\right] \\
\beta^{[\text{Kv4.3}]} &= 42\exp\left[-0.54\frac{VF}{RT}\right] \\
k_{co}^{[\text{Kv4.3}]} &= 100\exp\left[0.25\frac{VF}{RT}\right] \\
k_{oc}^{[\text{Kv4.3}]} &= 300\exp\left[-0.05\frac{VF}{RT}\right]
\end{aligned}
$$

$$
\begin{aligned}
\frac{dC_0^{[\mathrm{Kv4.3}]}}{dt} &= 1-(O^{[\mathrm{Kv4.3}]}+\sum_{i=1}^{4}C_i^{[\mathrm{Kv4.3}]}+\sum_{i=0}^{6}I_i^{[\mathrm{Kv4.3}]}) \\
\frac{dC_1^{[\mathrm{Kv4.3}]}}{dt} &= 4\alpha^{[\mathrm{Kv4.3}]}C_0^{[\mathrm{Kv4.3}]}-(\beta^{[\mathrm{Kv4.3}]}+3\alpha^{[\mathrm{Kv4.3}]}+k_{ci}^{[\mathrm{Kv4.3}]}f^3)C_1^{[\mathrm{Kv4.3}]}+2\beta^{[\mathrm{Kv4.3}]}C_2^{[\mathrm{Kv4.3}]}+\frac{k_{ic}^{[\mathrm{Kv4.3}]}}{f^3}I_1^{[\mathrm{Kv4.3}]} \\
\frac{dC_2^{[\mathrm{Kv4.3}]}}{dt} &= 3\alpha^{[\mathrm{Kv4.3}]}C_1^{[\mathrm{Kv4.3}]}-(2\beta^{[\mathrm{Kv4.3}]}+2\alpha^{[\mathrm{Kv4.3}]}+k_{ci}^{[\mathrm{Kv4.3}]}f^2)C_2^{[\mathrm{Kv4.3}]}+3\beta^{[\mathrm{Kv4.3}]}C_3^{[\mathrm{Kv4.3}]}+\frac{k_{ic}^{[\mathrm{Kv4.3}]}}{f^2}I_2^{[\mathrm{Kv4.3}]} \\
\frac{dC_3^{[\mathrm{Kv4.3}]}}{dt} &= 2\alpha^{[\mathrm{Kv4.3}]}C_2^{[\mathrm{Kv4.3}]}-(3\beta^{[\mathrm{Kv4.3}]}+\alpha^{[\mathrm{Kv4.3}]}+k_{ci}^{[\mathrm{Kv4.3}]}f)C_3^{[\mathrm{Kv4.3}]}+4\beta^{[\mathrm{Kv4.3}]}C_4^{[\mathrm{Kv4.3}]}+\frac{k_{ic}^{[\mathrm{Kv4.3}]}}{f}I_3^{[\mathrm{Kv4.3}]} \\
\frac{dC_4^{[\mathrm{Kv4.3}]}}{dt} &= \alpha^{[\mathrm{Kv4.3}]}C_3^{[\mathrm{Kv4.3}]}-(4\beta^{[\mathrm{Kv4.3}]}+k_o^{[\mathrm{Kv4.3}]}+k_{ci}^{[\mathrm{Kv4.3}]})C_4^{[\mathrm{Kv4.3}]}+k_{-o}^{[\mathrm{Kv4.3}]}O_4^{[\mathrm{Kv4.3}]}+k_{ic}^{[\mathrm{Kv4.3}]}I_4^{[\mathrm{Kv4.3}]} \\
\frac{dI_0^{[\mathrm{Kv4.3}]}}{dt} &= k_1^{[\mathrm{Kv4.3}]}f^4C_0^{[\mathrm{Kv4.3}]}-(\frac{k_{-1}^{[\mathrm{Kv4.3}]}}{f^4}+4\frac{\alpha^{[\mathrm{Kv4.3}]}}{f})I_0^{[\mathrm{Kv4.3}]}+\beta^{[\mathrm{Kv4.3}]}fI_1^{[\mathrm{Kv4.3}]} \\
\frac{dI_1^{[\mathrm{Kv4.3}]}}{dt} &= 4\frac{\alpha^{[\mathrm{Kv4.3}]}}{f}I_0^{[\mathrm{Kv4.3}]}+k_1^{[\mathrm{Kv4.3}]}f^3C_1^{[\mathrm{Kv4.3}]}-(f\beta^{[\mathrm{Kv4.3}]}+\frac{k_{ic}^{[\mathrm{Kv4.3}]}}{f^3}+3\frac{\alpha^{[\mathrm{Kv4.3}]}}{f})I_1^{[\mathrm{Kv4.3}]} \\
&\quad +2\beta^{[\mathrm{Kv4.3}]}fI_2^{[\mathrm{Kv4.3}]} \\
\frac{dI_2^{[\mathrm{Kv4.3}]}}{dt} &= 3\frac{\alpha^{[\mathrm{Kv4.3}]}}{f}I_1^{[\mathrm{Kv4.3}]}+k_1^{[\mathrm{Kv4.3}]}f^2C_2^{[\mathrm{Kv4.3}]}-(2f\beta^{[\mathrm{Kv4.3}]}+\frac{k_{ic}^{[\mathrm{Kv4.3}]}}{f^2}+2\frac{\alpha^{[\mathrm{Kv4.3}]}}{f})I_2^{[\mathrm{Kv4.3}]} \\
&\quad +3\beta^{[\mathrm{Kv4.3}]}fI_3^{[Kv43]} \\
\frac{dI_3^{[\mathrm{Kv4.3}]}}{dt} &= 2\frac{\alpha^{[\mathrm{Kv4.3}]}}{f}I_2^{[\mathrm{Kv4.3}]}+k_1^{[\mathrm{Kv4.3}]}fC_3^{[\mathrm{Kv4.3}]}-(3f\beta^{[\mathrm{Kv4.3}]}+\frac{k_{ic}^{[\mathrm{Kv4.3}]}}{f}+\frac{\alpha^{[\mathrm{Kv4.3}]}}{f})I_3^{[\mathrm{Kv4.3}]} \\
&\quad +4\beta^{[\mathrm{Kv4.3}]}fI_4^{[Kv43]} \\
\frac{dI_4^{[\mathrm{Kv4.3}]}}{dt} &= \frac{\alpha^{[\mathrm{Kv4.3}]}}{f}I_3^{[\mathrm{Kv4.3}]}+k_{ci}^{[\mathrm{Kv4.3}]}C_4^{[\mathrm{Kv4.3}]}-(4f\beta^{[\mathrm{Kv4.3}]}+k_{ic}^{[\mathrm{Kv4.3}]})I_4^{[\mathrm{Kv4.3}]} \\
\frac{dI_5^{[\mathrm{Kv4.3}]}}{dt} &= -(k_{io}^{[\mathrm{Kv4.3}]}+k_{56}^{[\mathrm{Kv4.3}]})I_5^{[\mathrm{Kv4.3}]}+k_{io}^{[\mathrm{Kv4.3}]}O^{[\mathrm{Kv4.3}]}+k_{65}^{[\mathrm{Kv4.3}]}I_6^{[\mathrm{Kv4.3}]} \\
\frac{dI_6^{[\mathrm{Kv4.3}]}}{dt} &= k_{56}^{[\mathrm{Kv4.3}]}I_5^{[\mathrm{Kv4.3}]}-k_{65}^{[\mathrm{Kv4.3}]}I_6^{[\mathrm{Kv4.3}]} \\
\frac{dO^{[\mathrm{Kv4.3}]}}{dt} &= k_{co}^{[\mathrm{Kv4.3}]}C_4^{[\mathrm{Kv4.3}]}-(k_{oc}^{[\mathrm{Kv4.3}]}+k_{oi}^{[\mathrm{Kv4.3}]})O^{[\mathrm{Kv4.3}]}+k_{io}^{[\mathrm{Kv4.3}]}I_5^{[\mathrm{Kv4.3}]}
\end{aligned}
$$

Kv4.3/KChIP2(b-d)

$$I_{\square} = \kappa_{\square}\, G_{\square}\, k_{1,\square}{}^4\, (A_{\text{fast},\square}\, k_{2_{\text{fast}},\square} + A_{\text{slow},\square}\, k_{2_{\text{slow}},\square})\, (V - E_{\text{K}})$$

Kv4.3/KChIP2b:

$$
\begin{aligned}
k_{1_\infty} &= \frac{1}{1+\exp\left[\frac{-2.97-V}{12.7}\right]} \\
k_{2_\infty} &= \frac{1}{1+\exp\left[\frac{57.4+V}{4.8}\right]} \\
\alpha &= 819 + \frac{-819}{1+\exp\left[\frac{V-14.6}{23.4}\right]} \quad \text{s}^{-1} \\
\beta &= \frac{185}{4(1+\exp\left[\frac{V+74}{13.2}\right]} \quad \text{s}^{-1} \\
\tau_{k_1} &= \frac{1000}{\alpha+4\beta} \\
\tau_{k_{2_{\text{fast}}}} &= 54.5 + 58.8\frac{\exp\left[-(30+V)\right]}{34.3} \\
\tau_{k_{2_{\text{slow}}}} &= 92 + 710.3\frac{\exp\left[-(30+V)\right]}{28.3} \\
\frac{A_{\text{fast}}}{A_{\text{fast}}+A_{\text{slow}}} &= 0.63 + \frac{0.63}{1+\exp\left[\frac{V-19.3}{8.5}\right]}
\end{aligned}
$$

Kv4.3/KChIP2d:

$$
\begin{aligned}
k_{1_\infty} &= \frac{1}{1+\exp\left[\frac{-2.3-V}{12.49}\right]} \\
k_{2_\infty} &= \frac{1}{1+\exp\left[\frac{61.1+V}{5}\right]} \\
\alpha &= 1044.6 + \frac{-1044.6}{1+\exp\left[\frac{V-17.3}{24}\right]} \quad \text{s}^{-1} \\
\beta &= \frac{240.9}{4(1+\exp\left[\frac{V+87.7}{16.3}\right])} \quad \text{s}^{-1} \\
\tau_{k_1} &= \frac{1000}{\alpha+4\beta} \\
\tau_{k_{2_{\text{fast}}}} &= 54.6 + 40\frac{\exp\left[-(30+V)\right]}{17.7} \\
\tau_{k_{2_{\text{slow}}}} &= 114.3 + 30.1\,\frac{\exp\left[-(V-10)\right]}{5.9} \\
\frac{A_{\text{fast}}}{A_{\text{fast}}+A_{\text{slow}}} &= 0.8 + \frac{0.08}{1+\exp\left[\frac{V-34}{4.3}\right]}
\end{aligned}
$$

Kv4.3/KCNE3 and Kv4.3/KCNE3/KChIP2

$$I_{\square} = \kappa_{\square}\, G_{\square}\, m_{1,\square}\, m_{2,\square}\, m_{3,\square}\, (V - E_{\mathrm{K}})$$

Kv4.3/KCNE3:

$$\begin{aligned}
m_{1_\infty} &= \frac{1}{1+\exp\left[\frac{6-V}{17.5}\right]}\\
m_{2_\infty} &= \frac{1}{1+\exp\left[\frac{72+V}{11.1}\right]}\\
\tau_{m_1} &= 6.1 \text{ ms}\\
\tau_{m_2} &= 220 \text{ ms}\\
\tau_{m_3} &= 979 \text{ ms}
\end{aligned}$$

Kv4.3/KCNE3/KChIP2:

$$\begin{aligned}
m_{1_\infty} &= \frac{1}{1+\exp\left[\frac{5-V}{17.5}\right]}\\
m_{2_\infty} &= \frac{1}{1+\exp\left[\frac{56+V}{11.1}\right]}\\
\tau_{m_1} &= 13.7\\
\tau_{m_2} &= 251\\
\tau_{m_3} &= 118
\end{aligned}$$

hERG

$$I_{\mathrm{hERG}} = \kappa_{\mathrm{hERG}}\, G_{\mathrm{hERG}}\, O^{[\mathrm{hERG}]}\, (V - E_{\mathrm{K}})$$

$$\begin{aligned}
\alpha_1^{[\mathrm{hERG}]} &= 0.022348 \exp[0.01176\, V]\\
\beta_1^{[\mathrm{hERG}]} &= 0.047002 \exp[-0.0631\, V]\\
\alpha_2^{[\mathrm{hERG}]} &= 0.013733 \exp[0.038198\, V]\\
\beta_2^{[\mathrm{hERG}]} &= 0.0000689 \exp[-0.04178\, V]\\
\alpha_i^{[\mathrm{hERG}]} &= 0.090821 \exp[0.023391\, V]\\
\beta_i^{[\mathrm{hERG}]} &= 0.006497 \exp[-0.03268\, V]
\end{aligned}$$

$$
\begin{aligned}
\frac{dC_1^{\text{[hERG]}}}{dt} &= -\alpha_1^{\text{[hERG]}} C_1^{\text{[hERG]}} + \beta_1^{\text{[hERG]}} C_2^{\text{[hERG]}} \\
\frac{dC_2^{\text{[hERG]}}}{dt} &= \alpha_1^{\text{[hERG]}} C_1^{\text{[hERG]}} - (\beta_1^{\text{[hERG]}} + k_f^{\text{[hERG]}}) C_2^{\text{[hERG]}} + k_b^{\text{[hERG]}} C_3^{\text{[hERG]}} \\
\frac{dC_3^{\text{[hERG]}}}{dt} &= k_f^{\text{[hERG]}} C_2^{\text{[hERG]}} - (\alpha_2^{\text{[hERG]}} + k_b^{\text{[hERG]}}) C_3^{\text{[hERG]}} + \beta_2^{\text{[hERG]}} O_1^{\text{[hERG]}} \\
\frac{dO_1^{\text{[hERG]}}}{dt} &= \alpha_2^{\text{[hERG]}} C_3^{\text{[hERG]}} - (\beta_2^{\text{[hERG]}} + \alpha_i^{\text{[hERG]}}) O_1^{\text{[hERG]}} + \beta_i^{\text{[hERG]}} I^{\text{[hERG]}} \\
\frac{dI^{[hERG]}}{dt} &= \alpha_i^{\text{[hERG]}} O_2^{\text{[hERG]}} - \beta_i^{\text{[hERG]}} I^{\text{[hERG]}}
\end{aligned}
$$

Kv7.1

$$
I_{\text{Kv7.1}} = \kappa_{\text{Kv7.1}}\, G_{\text{Kv7.1}}\, P_o^{[Kv7.1]}\, (V - E_{\text{K}})
$$

$$
\begin{aligned}
\alpha_1^{\text{[Kv7.1]}} &= 4.6 \exp\left[\frac{0.47\, VF}{RT}\right] \\
\beta_1^{\text{[Kv7.1]}} &= 33 \exp\left[\frac{-0.35\, VF}{RT}\right] \\
\alpha_2^{\text{[Kv7.1]}} &= 24 \exp\left[\frac{0.006\, VF}{RT}\right] \\
\beta_2^{\text{[Kv7.1]}} &= 19 \exp\left[\frac{-0.007\, VF}{RT}\right] \\
\epsilon^{\text{[Kv7.1]}} &= 4.6 \exp\left[\frac{0.8\, VF}{RT}\right] \\
\delta^{\text{[Kv7.1]}} &= 1.4 \exp\left[\frac{-0.7\, VF}{RT}\right]
\end{aligned}
$$

$$
\begin{aligned}
\frac{dC_1^{\text{[Kv7.1]}}}{dt} &= -\alpha_1^{\text{[Kv7.1]}} C_1^{\text{[Kv7.1]}} + \beta_1^{\text{[Kv7.1]}} C_2^{\text{[Kv7.1]}} \\
\frac{dC_2^{\text{[Kv7.1]}}}{dt} &= \alpha_1^{\text{[Kv7.1]}} C_1^{\text{[Kv7.1]}} - (\beta_1^{Kv7.1} + \alpha_2^{\text{[Kv7.1]}}) C_2^{\text{[Kv7.1]}} + \beta_2^{\text{[Kv7.1]}} O_1^{\text{[Kv7.1]}} \\
\frac{dO_1^{\text{[Kv7.1]}}}{dt} &= \alpha_2^{\text{[Kv7.1]}} C_2^{\text{[Kv7.1]}} - (\epsilon^{\text{[Kv7.1]}} + \beta_2^{\text{[Kv7.1]}}) O_1^{\text{[Kv7.1]}} + \delta^{\text{[Kv7.1]}} O_2^{\text{[Kv7.1]}} \\
\frac{dO_2^{\text{[Kv7.1]}}}{dt} &= \epsilon^{\text{[Kv7.1]}} O_1^{\text{[Kv7.1]}} - (\delta^{\text{Kv7.1}} + \lambda^{\text{[Kv7.1]}}) O_2^{\text{[Kv7.1]}} + \mu^{\text{[Kv7.1]}} I^{\text{[Kv7.1]}} \\
\frac{dI^{\text{[Kv7.1]}}}{dt} &= \lambda^{\text{[Kv7.1]}} O_2^{\text{[Kv7.1]}} - \mu^{\text{[Kv7.1]}} I^{\text{[Kv7.1]}}
\end{aligned}
$$

Kv7.4

$$I_{\text{Kv7.4}} = \kappa_{\text{Kv7.4}}\, G_{\text{Kv7.4}}\, d_1\, d_2\, (V - E_{\text{K}})$$

$$\begin{aligned} d_\infty &= \frac{1}{1 + \exp\left[\frac{-32-V}{17.4}\right]} \\ \tau_{d_{\text{fast}}} &= \frac{1}{\exp\left[9(-65 - V)\right] + \exp\left[0.03(-132.4 + V)\right]} \\ \tau_d &= 0.35\, \tau_{d_{\text{fast}}} + 0.65\, \tau_{d_{\text{slow}}} \end{aligned}$$

BK$_\alpha$ and BK$_{\alpha+\beta 1}$

$$I_{\text{BK}} = \kappa_{\text{BK}} G_{\text{BK}} P_{\text{BK}} (V - E_{\text{K}})$$

$$\begin{aligned} \delta_{xi} &= \delta_{o_i} \exp\left[\frac{z_\delta F V}{RT}\right] \\ \gamma_{xi} &= \gamma_{o_i} \exp\left[\frac{z_\gamma F V}{RT}\right] \quad \text{where } \ i = 0 \ldots 4 \end{aligned}$$

$$\begin{aligned} J_c &= \exp\left[\frac{z_J F (V - V_{hc})}{RT}\right] \\ J_o &= \exp\left[\frac{z_J F (V - V_{ho})}{RT}\right] \end{aligned}$$

$$\begin{aligned} f_{co} &= \frac{1}{1 + 4j_c + 6{j_c}^2 + 4{j_c}^3 + {j_c}^4} \\ f_{c1} &= \frac{4j_c}{1 + 4j_c + 6{j_c}^2 + 4{j_c}^3 + {j_c}^4} \\ f_{c2} &= \frac{6{j_c}^2}{1 + 4j_c + 6{j_c}^2 + 4{j_c}^3 + {j_c}^4} \\ f_{c3} &= \frac{4{j_c}^3}{1 + 4j_c + 6{j_c}^2 + 4{j_c}^3 + {j_c}^4} \\ f_{c4} &= \frac{{j_c}^4}{1 + 4j_c + 6{j_c}^2 + 4{j_c}^3 + {j_c}^4} \end{aligned}$$

$$
\begin{aligned}
f_{oo} &= \frac{1}{1+4j_o+6{j_o}^2+4{j_o}^3+{j_o}^4} \\
f_{o1} &= \frac{4j_o}{1+4j_o+6{j_o}^2+4{j_o}^3+{j_o}^4} \\
f_{o2} &= \frac{6{j_o}^2}{1+4j_o+6{j_o}^2+4{j_o}^3+{j_o}^4} \\
f_{o3} &= \frac{4{j_o}^3}{1+4j_o+6{j_o}^2+4{j_o}^3+{j_o}^4} \\
f_{o4} &= \frac{{j_o}^4}{1+4j_o+6{j_o}^2+4{j_o}^3+{j_o}^4}
\end{aligned}
$$

$$
\begin{aligned}
\delta &= (\delta_{x1} * f_{co}) + (\delta_{x2} * f_{c1}) + (\delta_{x3} * f_{c2}) + (\delta_{x4} * f_{c3}) + (\delta_{x5} * f_{c4}) \\
\gamma &= (\gamma_{x1} * f_{oo}) + (\gamma_{x2} * f_{o1}) + (\gamma_{x3} * f_{o2}) + (\gamma_{x4} * f_{o3}) + (\gamma_{x5} * f_{o4})
\end{aligned}
$$

$$
\tau_{\mathrm{BK}_{\alpha/\alpha+\beta1}} = \frac{1}{\gamma+\delta}
$$

$$
P_{\mathrm{BK}_{\alpha/\alpha+\beta1_{ss}}} = \frac{1}{1+\left(\frac{1+\exp[z_J F(V-V_{hc})/RT]}{1+\exp[z_J F(V-V_{ho})/RT]}\right)^4 \left(\frac{1+\mathrm{Ca}_i/K_c}{1+\mathrm{Ca}_i/K_o}\right)^8 \frac{\exp[-z_L FV/RT]}{L}}
$$

$\mathbf{BK}_{\alpha+\beta3}$

$$
I_{\mathrm{BK}_{\alpha+\beta3}} = \kappa_{\mathrm{BK}_{\alpha+\beta3}} G_{\mathrm{BK}_{\alpha+\beta3}} P_{o\mathrm{BK}_{\alpha+\beta3}} (V - E_{\mathrm{K}})
$$

$$
P_{\mathrm{BK}_{\alpha+\beta3_o}} = \frac{(O_n^{[\alpha+\beta3]} + I_n^{[\alpha+\beta3]})}{(C_n^{[\alpha+\beta3]} + O_n^{[\alpha+\beta3]} + I_n^{[\alpha+\beta3]})}
$$

$$
\begin{aligned}
k_f &= k_{f_i} \exp\left[\frac{z_f VF}{RT}\right] \\
k_r &= k_{r_i} \exp\left[\frac{z_r VF}{RT}\right] \\
k_b &= k_{b_i} \exp\left[\frac{z_b VF}{RT}\right] \\
k_u &= k_{u_i} \exp\left[\frac{z_u VF}{RT}\right] \qquad \text{where } i = 0,1,2,3,4
\end{aligned}
$$

$$
\begin{aligned}
\frac{dC_0^{[\alpha+\beta 3]}}{dt} &= k_c\, C_1^{[\alpha+\beta 3]} - (4\,[\mathrm{Ca}]_i + k_{f_0})\, C_0^{[\alpha+\beta 3]} + k_{r_0}\, O_0^{[\alpha+\beta 3]} \\
\frac{dI_0^{[\alpha+\beta 3]}}{dt} &= k_i\, I_1^{[\alpha+\beta 3]} - (4\,[\mathrm{Ca}]_i + k_{b_0})\, I_0^{[\alpha+\beta 3]} + k_{b_0}\, O_0^{[\alpha+\beta 3]} \\
\frac{dO_0^{[\alpha+\beta 3]}}{dt} &= k_o\, O_1^{[\alpha+\beta 3]} - (4\,[\mathrm{Ca}]_i + k_{r_0} + k_{b_0})\, O_0^{[\alpha+\beta 3]} + k_{f_0}\, C_0^{[\alpha+\beta 3]} + k_{u_0}\, I_0^{[\alpha+\beta 3]} \\
\frac{dC_4^{[\alpha+\beta 3]}}{dt} &= [\mathrm{Ca}]_i\, C_3^{[\alpha+\beta 3]} - (4\,k_c + k_{f_4}) C_4^{[\alpha+\beta 3]} + k_{r_4}\, O_4^{[\alpha+\beta 3]} \\
\frac{dO_4^{[\alpha+\beta 3]}}{dt} &= [\mathrm{Ca}]_i\, O_3^{[\alpha+\beta 3]} - (4\,k_o + k_{r_4} + k_{b_4}) O_4^{[\alpha+\beta 3]} + k_{f_4}\, C_4^{[\alpha+\beta 3]} + k_{u_4}\, I_4^{[\alpha+\beta 3]} \\
\frac{dI_4^{[\alpha+\beta 3]}}{dt} &= [\mathrm{Ca}]_i\, I_3^{[\alpha+\beta 3]} - (4\,k_i + k_{u_4}) I_4^{[\alpha+\beta 3]} + k_{b_4}\, O_4^{[\alpha+\beta 3]} \\
\frac{dC_n^{[\alpha+\beta 3]}}{dt} &= (5-n)[\mathrm{Ca}]_i\, C_{n-1}^{[\alpha+\beta 3]} + (n+1)k_c\, C_{n+1}^{[\alpha+\beta 3]} - (n\,k_c + (5-n-1)[\mathrm{Ca}]_i - k_{f_n}) C_n^{[\alpha+\beta 3]} \\
&\quad + k_{r_n}\, O_n^{[\alpha+\beta 3]} \\
\frac{dO_n^{[\alpha+\beta 3]}}{dt} &= (5-n)[\mathrm{Ca}]_i\, O_{n-1}^{[\alpha+\beta 3]} + (n+1)k_o\, O_{n+1}^{[\alpha+\beta 3]} - (n\,k_o + (5-n-1)[\mathrm{Ca}]_i - k_{r_n} + k_{b_n}) O_n^{[\alpha+\beta 3]} \\
&\quad + k_{f_n}\, C_n^{[\alpha+\beta 3]} + k_{u_n}\, I_n^{[\alpha+\beta 3]} \\
\frac{dI_n^{[\alpha+\beta 3]}}{dt} &= (5-n)[\mathrm{Ca}]_i\, I_{n-1}^{[\alpha+\beta 3]} + (n+1)k_i\, I_{n+1}^{[\alpha+\beta 3]} - (n\,k_i + (5-n-1)[\mathrm{Ca}]_i \\
&\quad - k_{u_n}) I_n^{[\alpha+\beta 3]} + k_{b_n}\, O_n^{[\alpha+\beta 3]} \qquad \text{where } n = 1, 2, 3
\end{aligned}
$$

$\mathbf{BK}_{\alpha+\beta 4}$

$$
I_{\mathrm{BK}_{\alpha+\beta 4}} = \kappa_{\mathrm{BK}_{\alpha+\beta 4}} G_{\mathrm{BK}_{\alpha+\beta 4}} P_{\mathrm{BK}_{\alpha+\beta 4}} (V - E_{\mathrm{K}})
$$

$$
\begin{aligned}
P_{\mathrm{BK}_{\alpha+\beta 4_{ss}}} &= \frac{1}{1 + \frac{(1+J+K+JKE)^4}{L(1+KC+JKCDE)^4}} \\
L &= L_0 \exp\left[\frac{-z_l F V}{RT}\right] \\
J &= \exp\left[\frac{-z_j F V}{RT}\right] \\
K &= \frac{[\mathrm{Ca}^{2+}]}{k_c} \\
D &= \exp\left[\frac{-z_j F (V_{ho} - V_{hc)}}{RT}\right] \\
C &= \frac{k_c}{k_o} \\
\tau_{\mathrm{BK}_{\alpha+\beta_4}} &= \tau_{\mathrm{BK}_\alpha} + 30
\end{aligned}
$$

$\mathbf{SK_2}$

$$I_{\text{SK2}} = \kappa_{\text{SK}_2} G_{\text{SK}_2} P_{o\text{SK}_2}(V - E_{\text{K}})$$

$$P_{o\text{SK}_{2ss}} = P_{0,max} \frac{[\text{Ca}^{2+}]_i^{2.2}}{(0.74)^{2.2} + [\text{Ca}^{2+}]_i^{2.2}}$$

$$\tau_{\text{SK}_2}^{-1} = -1.3 + 45.5[\text{Ca}^{2+}]_i$$

$\mathbf{SK_3}$

$$I_{\text{SK}_3} = \kappa_{\text{SK}_3} G_{\text{SK}_3} P_{\text{SK}_3}(V - E_{\text{K}})$$

$$\begin{aligned} P_{\text{SK}_{3ss}} &= \frac{[\text{Ca}^{2+}]_i^5}{(0.6)^5 + [\text{Ca}^{2+}]_i^5} \\ \tau_{\text{SK}_3} &= 12.95 \text{ ms} \end{aligned}$$

$\mathbf{SK_4}$

$$I_{\text{SK}_4} = \kappa_{\text{SK}_4} G_{\text{SK}_4} P_{o\text{SK}_4}(V - E_{\text{K}})$$

$$\begin{aligned} P_{o\text{SK}_{4ss}} &= \frac{[\text{Ca}^{2+}]_i^{3.2}}{(0.095)^{3.2} + [\text{Ca}^{2+}]_i^{3.2}} \\ \tau_{\text{SK}_4} &= 5.8 \text{ ms} \end{aligned}$$

Kir7.1

$$I_{\text{Kir7.1}} = \kappa_{\text{Kir7.1}} G_{\text{Kir7.1}} P_{o\text{Kir7.1}} Y (V - E_{\text{K}})$$

$$\begin{aligned} P_{o\text{Kir7.1}_{ss}} &= 0.083 \ \exp\left[-0.018 \ V\right] \\ Y &= 0.65 K_o^{0.095} \\ \tau_{\text{Kir7.1}} &= 2 + 0.01 \ V \end{aligned}$$

T-type

$$I_{T\text{-}type} = \kappa_{\text{T-type}} \ G_{\text{T-type}} \ a \ c \ (V - E_{\text{Ca}})$$

$$
\begin{aligned}
a_{\infty} &= \frac{1}{1+\exp\left[\frac{-28.6-V}{8.9}\right]} \\
c_{\infty} &= \frac{1}{1+\exp\left[\frac{72.4+V}{4.8}\right]}
\end{aligned}
$$

$$
\begin{aligned}
\tau_{\mathrm{a}} &= 1.7+\frac{9.87}{1+\exp\left[\frac{V+39}{7.6}\right]} \\
\tau_{\mathrm{c}} &= 13.7+\frac{5369.7}{1+\exp\left[\frac{V+108.5}{11.24}\right]}
\end{aligned}
$$

L-type

$$
I_{\textit{L-type}} = \kappa_{\text{L-type}}\, G_{\text{L-type}}\; d\; f\; f_{\mathrm{Ca}}(V-E_{\mathrm{Ca}})
$$

$$
\begin{aligned}
d_{\infty} &= \frac{1}{(1+\exp\left[\frac{-(V+18.9)}{8.8}\right])} \\
f_{\infty} &= \frac{1}{(1+\exp\left[\frac{(V+53)}{11}\right])} \\
\tau_{d} &= \frac{28.57(1-\exp\left[\frac{-(V+10)}{6.24}\right])}{(1+\exp\left[\frac{-(V+10)}{6.24}\right])(V+10)} \\
\tau_{f} &= \frac{50}{1+\exp\left[\frac{-(V+10)^2}{881}\right]} \\
f_{\mathrm{Ca}} &= \frac{1}{(1+\frac{[\mathrm{Ca}^{2+}]}{0.006})}
\end{aligned}
$$

CaCC ANNO1

$$
I_{\mathrm{CaCC}} = \kappa_{\mathrm{CaCC}}\; G_{\mathrm{CaCC}}\; c_c\; (V-E_{\mathrm{Cl}})
$$

$$
c_{c_{\infty}} = \frac{1}{1+K_2\,(\frac{K_1^2}{[\mathrm{Ca}^{2+}]^2}+\frac{K_1}{[\mathrm{Ca}^{2+}]}+1)}
$$

$$
\begin{aligned}
K_1 &= 214\;\exp\left[0.13\,FV/RT\right] \quad \text{nM} \\
K_2 &= 0.58\;\exp\left[-0.24\,FV/RT\right] \quad \text{nM}
\end{aligned}
$$

$$
\frac{1}{\tau_{c_c}} = \frac{0.38}{\frac{K_1^2}{[\mathrm{Ca}^{2+}]^2}+\frac{K_1}{[\mathrm{Ca}^{2+}]}+1} + 0.38\;K_2 \quad s^{-1}
$$

P2X4R

$$I_{\text{P2X4}} = \kappa_{\text{P2X4}} G_{\text{P2X4}} P_{\text{act}} P_{\text{des}} (V - E_{\text{P2X4}})$$

$$E_{\text{P2X4}} = \frac{RT}{F} \ln \frac{[\text{Na}^+]_o + \frac{P_\text{K}}{P_\text{Na}}[\text{K}^+]_o + 4\frac{P'_\text{Ca}}{P_\text{Na}}[\text{Ca}^+]_o}{[\text{Na}^+]_i + \frac{P_\text{K}}{P_\text{Na}}[\text{K}^+]_i + 4\frac{P'_\text{Ca}}{P_\text{Na}}[\text{Ca}^+]_i e^{(\frac{FV}{RT})}}$$

$$P'_\text{Ca} = \frac{P_\text{Ca}}{1 + e^{FVRT}}$$

$$P_{\text{act}_{ss}} = \frac{[\text{ATP}]_o^{1.6}}{32^{1.6} + [\text{ATP}]_o^{1.6}}$$

$$\begin{aligned} \tau_\text{des} &= 1000 + \frac{20000}{1 + e^{0.09(-47.32 + [\text{ATP}]_o)}} \\ \tau_\text{deac} &= \frac{540}{(1 + e^{0.02(-10 - [\text{ATP}]_o)})(1 + e^{0.00065(-80 + [\text{ATP}]_o)})} \end{aligned}$$

Gap junctions

$$I_\text{GJ} = \kappa_\text{GJ}\, G_\text{GJ}\, P_\text{GJ}\, (V_j)$$

$$G_j = \frac{G_\text{max} - G_{\text{min}_{n/p}}}{1 + \exp[-A(V_j - V_{h_{n/p}})]} + G_{\text{min}_{n/p}}$$

$$\begin{aligned} G_j(\text{Type I}) &= 0.35 + \frac{90}{\sqrt{2\pi\sigma^2}\ \exp\left[\frac{-V_j^2}{2\sigma^2}\right]} \\ G_j(\text{Type II}) &= 0.25 + \frac{55}{\sqrt{2\pi\sigma^2}\ \exp\left[\frac{-V_j^2}{2\sigma^2}\right]} \end{aligned}$$

$$\begin{aligned} \tau_j(\text{Type I}) &= 9 + \frac{40 \times 10^3}{1 + 0.0033\, V_j^2} \\ \tau_j(\text{Type II}) &= 120 + \frac{7.4 \times 10^3}{1 + 0.0026\, V_j^2} \end{aligned}$$

NCX

$$I_\text{NCX} = \kappa_\text{NCX}(0.00025 e^{\frac{-0.65VF}{RT}} \frac{e^{\frac{VF}{RT}}[\text{Na}^+]_i^3[\text{Ca}^{2+}]_o - [\text{Na}^+]_o^3[\text{Ca}^{2+}]_i}{1 + 0.0001[\text{Na}^+]_i^3[\text{Ca}^{2+}]_o + [\text{Na}^+]_o^3[\text{Ca}^{2+}]_i})$$

PMCA

$$I_{\text{PMCA}} = \kappa_{\text{PMCA}} \frac{1.15}{1 + \frac{0.0005}{[\text{Ca}^{2+}]_i}}$$

Na^+/K^+ pump

$$\begin{aligned}
I_{\text{NaK}} &= \kappa_{\text{NaK}} I_{\text{NaK}_{\text{max}}} f_{\text{NaK}} I_{\text{NaK,nai}} I_{\text{NaK,ko}} \\
I_{NaK,nai} &= \frac{1}{1 + (\frac{10}{[\text{Na}^+]_i})^2} \\
I_{NaK,ko} &= \frac{1}{1 + (\frac{1.5}{[\text{K}^+]_o})} \\
f_{\text{NaK}} &= \frac{1}{1 + 0.12\, e^{\frac{-0.1VF}{RT}} + 0.037\, \sigma\, e^{\frac{VF}{RT}}} \\
\sigma &= \frac{1}{7(e^{\frac{[\text{Na}^+]_o}{67.3}} - 1)}
\end{aligned}$$

K^+ and Cl^- background current

$$I_{\text{bgK}} = \kappa_{\text{bgK}} G_{\text{bgK}} (V - E_{\text{K}})$$
$$I_{\text{bgCl}} = \kappa_{\text{bgCl}} G_{\text{bgCl}} (V - E_{\text{Cl}})$$

Effect of PIP_2 on hERG

$$\begin{aligned}
\alpha_1^{\text{[hERG]}} &= 1.083 \exp[3.14 \times 10^{-4}\, V] \\
\beta_1^{\text{[hERG]}} &= 2.45 \times 10^{-3} \exp[-10.2 \times 10^{-3}\, V] \\
\alpha_2^{\text{[hERG]}} &= 7.76 \times 10^{-3} \exp[3.49 \times 10^{-2}\, V] \\
\beta_2^{\text{[hERG]}} &= 2.31 \times 10^{-4} \exp[-4.84 \times 10^{-2}\, V] \\
\alpha_3^{\text{[hERG]}} &= 7.63 \times 10^{-2} \exp[0.513 \times 10^{-3}\, V] \\
\beta_3^{\text{[hERG]}} &= 1.41 \times 10^{-2} \exp[-3.84 \times 10^{-3}\, V] \\
\alpha_i^{\text{[hERG]}} &= 5.68 \times 10^{-2} \exp[-2.7 \times 10^{-2}\, V] \\
\beta_i^{\text{[hERG]}} &= 8.66 \times 10^{-2} \exp[2.58 \times 10^{-2}\, V] \\
\alpha_{ic}^{\text{[hERG]}} &= 5.37 \times 10^{-2} \exp[2.32 \times 10^{-2}\, V] \\
\beta_{ic}^{\text{[hERG]}} &= (\beta_3^{\text{[hERG]}} \alpha_i^{\text{[hERG]}} \alpha_{ic}^{\text{[hERG]}})/(\alpha_3^{\text{[hERG]}} \beta_3^{\text{[hERG]}})
\end{aligned}$$

$$
\begin{aligned}
\frac{dC_1^{\text{[hERG]}}}{dt} &= -\alpha_1^{\text{[hERG]}} C_1^{\text{[hERG]}} + \beta_1^{\text{[hERG]}} C_2^{\text{[hERG]}} \\
\frac{dC_2^{\text{[hERG]}}}{dt} &= \alpha_1^{\text{[hERG]}} C_1^{\text{[hERG]}} - (\beta_1^{\text{[hERG]}} + \alpha_2^{\text{[hERG]}}) C_2^{\text{[hERG]}} + \beta_2^{\text{[hERG]}} C_3^{\text{[hERG]}} \\
\frac{dC_3^{\text{[hERG]}}}{dt} &= \alpha_2^{\text{[hERG]}} C_2^{\text{[hERG]}} - (\alpha_3^{\text{[hERG]}} + \beta_2^{\text{[hERG]}} + \alpha_{ic}^{\text{[hERG]}}) C_3^{\text{[hERG]}} + \beta_3^{\text{[hERG]}} O^{\text{[hERG]}} + \beta_{ic}^{\text{[hERG]}} I^{\text{[hERG]}} \\
\frac{dO^{\text{[hERG]}}}{dt} &= \alpha_3^{\text{[hERG]}} C_3^{\text{[hERG]}} - (\beta_3^{\text{[hERG]}} + \beta_i^{\text{[hERG]}}) O^{\text{[hERG]}} + \alpha_i^{\text{[hERG]}} I^{\text{[hERG]}} \\
\frac{dI^{[hERG]}}{dt} &= \beta_i^{\text{[hERG]}} O^{\text{[hERG]}} - (\alpha_i^{\text{[hERG]}} + \beta_{ic}^{\text{[hERG]}}) I^{\text{[hERG]}} + \alpha_{ic}^{\text{[hERG]}} C_3^{\text{[hERG]}}
\end{aligned}
$$

Effect of PIP_2 on Kir7.1

$$P_{\text{Kir7.1}_{ss}} = 0.17\ e^{-0.01144\ V}$$
