# Supplementary figures and images for: Reconstruction of Cell Surface Densities of Ion Pumps, Exchangers, and Channels from mRNA Expression, Conductance Kinetics, Whole-Cell Calcium, and Current-Clamp Voltage Recordings, with an Application to Human Uterine Smooth Muscle Cells
Source: PLoS Comput Biol. 2016 Apr 22;12(4):e1004828. doi: 10.1371/journal.pcbi.1004828 (PMC4841602; doi:10.1371/journal.pcbi.1004828)

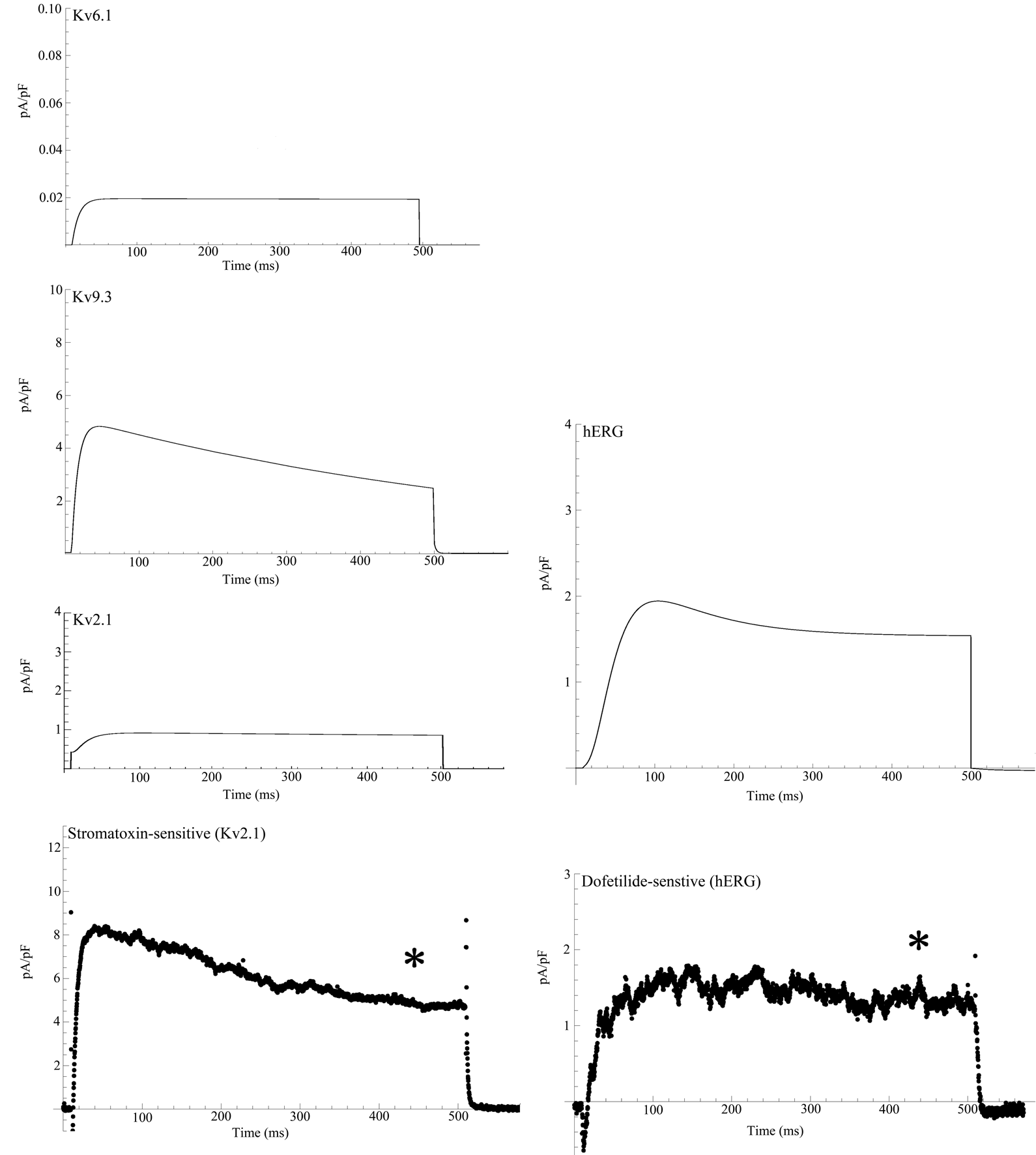

Supplement: S1 Fig — (TIF) [file pcbi.1004828.s006.tif]
